# Robust Estimation of Polychoric Correlation

**DOI:** 10.1017/psy.2025.10066

**Published:** 2025-12-17

**Authors:** Max Welz, Patrick Mair, Andreas Alfons

**Affiliations:** 1 Department of Psychology, University of Zurich, Switzerland; 2 Department of Econometrics, Erasmus University Rotterdam, The Netherlands; 3 Department of Psychology, Harvard University, USA

**Keywords:** careless responding, model misspecification, polychoric correlation, robust estimation

## Abstract

Polychoric correlation is often an important building block in the analysis of rating data, particularly for structural equation models. However, the commonly employed maximum likelihood (ML) estimator is highly susceptible to misspecification of the polychoric correlation model, for instance, through violations of latent normality assumptions. We propose a novel estimator that is designed to be robust against partial misspecification of the polychoric model, that is, when the model is misspecified for an unknown fraction of observations, such as careless respondents. To this end, the estimator minimizes a robust loss function based on the divergence between observed frequencies and theoretical frequencies implied by the polychoric model. In contrast to existing literature, our estimator makes no assumption on the type or degree of model misspecification. It furthermore generalizes ML estimation, is consistent as well as asymptotically normally distributed, and comes at no additional computational cost. We demonstrate the robustness and practical usefulness of our estimator in simulation studies and an empirical application on a Big Five administration. In the latter, the polychoric correlation estimates of our estimator and ML differ substantially, which, after further inspection, is likely due to the presence of careless respondents that the estimator helps identify.

## 1 Introduction

Ordinal data are ubiquitous in psychology and related fields. With such data, e.g., arising from responses to rating scales, it is often recommended to estimate correlation matrices through polychoric correlation coefficients (e.g., Foldnes & Grønneberg, 2022; Garrido et al., 2013; Holgado–Tello et al., 2010). The resulting polychoric correlation matrix is an important building block in subsequent multivariate models like factor analysis models and structural equation models (SEMs), as well as in exploratory methods like principal component analysis, multidimensional scaling, and clustering techniques (see, e.g., Mair (2018) for an overview). An individual polychoric correlation coefficient is the population correlation between two underlying latent variables that are postulated to have generated the observed categorical data through an unobserved discretization process. Traditionally, it is assumed that the two latent variables are standard bivariate normally distributed (Pearson, 1901) to estimate the polychoric correlation coefficient from observed ordinal data. Estimation of this latent normality model, called the *polychoric model*, is commonly carried out via maximum likelihood (ML) (Olsson, 1979). However, recent work has demonstrated that ML estimation of polychoric correlation is highly sensitive to violations of the assumption of underlying normality. Violations of this assumption result in a misspecified polychoric model, which can lead to substantially biased estimates of its parameters and those of subsequent multivariate models (Foldnes & Grønneberg, 2019, 2020; Grønneberg & Foldnes, 2022; Jin & Yang-Wallentin, 2017), particularly SEMs using *diagonally weighted least squares* (Foldnes & Grønneberg, 2022), where the latter is based on weights derived under latent normality.

Motivated by the recent interest in non-robustness of ML, we study the estimation of the polychoric model under a misspecification framework stemming from the robust statistics literature (e.g., Huber & Ronchetti, 2009). In this setup, which we call *partial* misspecification here, the polychoric model is potentially misspecified for an unknown (and possibly zero-valued) fraction of observations. Heuristically, the model is misspecified such that the affected subset of observations contains little to no relevant information for the parameter of interest, the polychoric correlation coefficient. Examples of such uninformative observations include careless responses, misresponses, or responses due to item misunderstanding. Especially careless responding has been identified as a major threat to the validity of questionnaire-based research findings (e.g., Credé, 2010; Huang, Liu, et al., 2015; Meade & Craig, 2012; Welz et al., 2024; Woods, 2006). We demonstrate that already a small fraction of uninformative observations (such as careless respondents) can result in considerably biased ML estimates.

As a remedy and our main contribution, we propose a novel way to estimate the polychoric model that is robust to partial model misspecification. Essentially, the estimator poses the question “What is the best fit that can be achieved with the polychoric model for (the majority of) the data at hand?” The estimator compares the observed frequency of each contingency table cell with its expected frequency under the polychoric model, and automatically downweights cells whose observed frequencies cannot be fitted sufficiently well. As such, our estimator generalizes the ML estimator, but, in contrast to ML, does not crucially rely on the correct specification of the model. Specifically, our estimator allows the model to be misspecified for an unknown fraction of uninformative responses in a sample, but makes *no assumption* on the type, magnitude, or location of potential misspecification. The estimator is designed to identify such responses and to simultaneously reduce their influence so that the polychoric model can be accurately estimated from the remaining responses generated by latent normality. Conversely, if the polychoric model is correctly specified, that is, latent normality holds true for all observations, our estimator and ML estimation are asymptotically equivalent. As such, our proposed estimator can be thought of as a generalized ML estimator that is robust to potential partial model misspecification, due to, for instance (but not limited to), careless responding. We show that our robust estimator is consistent, asymptotically normal, and fully efficient under the polychoric model, while possessing similar asymptotic properties under misspecification, and it comes at no additional computational cost compared to ML.

The partial misspecification framework in this article is fundamentally different from that considered in recent literature on misspecified polychoric models. In this literature (e.g., Foldnes & Grønneberg, 2019, 2020, 2022; Grønneberg & Foldnes, 2022; Jin & Yang-Wallentin, 2017; Lyhagen & Ornstein, 2023), the polychoric model is misspecified in the sense that *all* (unobserved) realizations of the latent continuous variables come from a distribution that is nonnormal. Under this framework, which is also known as *distributional misspecification*, the parameter of interest is the correlation coefficient of the latent nonnormal distribution, and all observations are informative for this parameter. While the distributional misspecification framework led to novel insights regarding (the lack of) robustness in ML estimation of polychoric correlation, the partial misspecification framework of this article can provide complimentary insights regarding the effects of a fraction of uninformative observations in a sample (such as careless responses), which is our primary objective.

Nevertheless, while our estimator is designed to be robust to partial misspecification caused by some uninformative responses, it can in some situations also provide a robustness gain under distributional misspecification. It turns out that if a nonnormal latent distribution differs from a normal distribution mostly in the tails, our estimator produces less biased estimates than ML because it can downweigh observations that are farther from the center.

To enhance accessibility and adoption by empirical researchers, an implementation of our proposed methodology in R (R Core Team, 2024) is freely available in the package robcat (for “ROBust CATegorical data analysis”; Welz et al., 2025) on CRAN (the Comprehensive R Archive Network) at https://CRAN.R-project.org/package=robcat. Replication materials for all numerical results in this article are provided on GitHub at https://github.com/mwelz/robust-polycor-replication. Proofs, derivations, and additional simulations can be found in the Supplementary Material.

This article is structured as follows. We start with reviewing related literature (Section 2) followed by the polychoric correlation model and ML estimation thereof (Section 3). Afterward, we elaborate on the partial misspecification framework (Section 4) and introduce our robust generalized ML estimator, including its statistical properties (Section 5). These properties are then examined by a simulation study in which we vary the misspecification fraction systematically, and compare the result to the commonly employed standard ML estimator (Section 6). Subsequently, we demonstrate the practical usefulness in an empirical application on a Big Five administration (Goldberg, 1992) by Arias et al. (2020), where we find evidence of careless responding, manifesting in differences in polychoric correlation estimates of as much as 0.3 between our robust estimator and ML (Section 7). We then investigate the performance of the estimator under distributional misspecification (Section 8) and conclude with a discussion of the results and avenues for further research (Section 9).

## 2 Related literature

ML estimation of polychoric correlations was originally believed to be fairly robust to slight to moderate distributional misspecification (Coenders et al., 1997; Flora & Curran, 2004; Li, 2016; Maydeu-Olivares, 2006). This belief was based on simulations that generated data for nonnormal latent variables via the Vale–Maurelli (VM) method (Vale & Maurelli, 1983), which were then discretized to ordinal data. However, Grønneberg & Foldnes (2019) show that the distribution of ordinal data generated in this way is indistinguishable from that of ordinal data stemming from discretizing normally distributed latent variables.^1^ In other words, simulation studies that ostensibly modeled nonnormality did in fact model normality. Simulating ordinal data in a way that ensures proper violations of latent normality (Grønneberg & Foldnes, 2017) reveals that polychoric correlation is in fact highly susceptible to distributional misspecification, resulting in possibly large biases (Foldnes & Grønneberg, 2020, 2022; Grønneberg & Foldnes, 2022; Jin & Yang-Wallentin, 2017). Consequently, it is recommended to test for the validity of the latent normality assumption, for instance, by using the bootstrap test of Foldnes & Grønneberg (2020).

Another source of model misspecification occurs when the polychoric model is only misspecified for an uninformative subset of a sample (partial misspecification), where, in the context of this article, the term “uninformative” refers to an absence of relevant information for polychoric correlation, for instance, in careless responses. Careless responding *“occurs when participants are not basing their response on the item content,”* for instance, when a participant is *“unmotivated to think about what the item is asking”* (Ward & Meade, 2023). It has been shown to be a major threat to the validity of research results through a variety of psychometric issues, such as reduced scale reliability (Arias et al., 2020) and construct validity (Kam & Meyer, 2015), attenuated factor loadings, improper factor structure, and deteriorated model fit in factor analyses (Arias et al., 2020; Huang, Bowling, et al., 2015; Woods, 2006), as well as inflated type I or type II errors in hypothesis testing (Arias et al., 2020; Huang, Liu, et al., 2015; Maniaci & Rogge, 2014; McGrath et al., 2010; Woods, 2006). Careless responding is widely prevalent (Bowling et al., 2016; Meade & Craig, 2012; Ward & Meade, 2023) with most estimates on its prevalence ranging from 10% to 15% of study participants (Curran, 2016; Huang et al., 2012; Huang, Liu, et al., 2015; Meade & Craig, 2012), while already a prevalence 5%–10% can jeopardize the validity of research findings (Arias et al., 2020; Credé, 2010; Welz et al., 2024; Woods, 2006). In fact, Ward & Meade (2023) conjecture that careless responding is likely present in all survey data. However, to the best of our knowledge, the effects of careless responding on estimates of the polychoric model have not yet been studied.

Existing model-based approaches to account for careless responding in various models typically explicitly model carelessness through mixture models (e.g., Arias et al., 2020; Steinmann et al., 2022; Ulitzsch, Pohl, et al., 2022; Ulitzsch, Yildirim-Erbasli, et al., 2022; Van Laar & Braeken, 2022). In contrast, our method does not model carelessness since we refrain from making assumptions on how the polychoric model might be misspecified. Another way to address careless responding is to directly detect them through person-fit indices and subsequently remove them from the sample (e.g., Patton et al., 2019). As a primary difference, our method simultaneously downweights aberrant observations during estimation rather than removing them. We refer to Alfons & Welz (2024) for a detailed overview of methods addressing careless responding in various settings.

Conceptually related to our approach, Itaya & Hayashi (2025) propose a way to robustly estimate parameters in item response theory (IRT) models. Their approach is conceptually similar to ours in the sense that it is based on minimizing a notion of divergence between an empirical density (from observed data) and a theoretical density of the IRT model. Like our approach, they achieve robustness by implicitly downweighting responses that the postulated model cannot fit well. Methodologically, our approach is different from Itaya & Hayashi (2025) because our method is based on *C*-estimation (Welz, 2024), which is designed specifically for categorical data, while they use *density power divergence estimation* (DPD) theory (Basu et al., 1998), which is not restricted to categorical data.^2^ A relevant consequence is that our estimator is fully efficient, whereas DPD estimators lose efficiency as a price for gaining robustness. To the best of our knowledge, DPD estimators have not yet been studied for estimating polychoric correlation.

Another related branch of literature is that of outlier detection in contingency tables (see Sripriya et al. (2020) for a recent overview). In this literature, an outlier is a contingency table cell whose observed frequency is *“markedly deviant”* from those of the remaining cells (Sripriya et al., 2020). This literature is agnostic with respect to the observed contingency table and therefore does not impose a parameterization on each cell’s probability. In contrast, the polychoric correlation model imposes such a parametrization through the assumption of latent bivariate normality. Another difference is that we are not primarily interested in outlier detection, but robust estimation of model parameters.

An alternative way to gain robustness against violations of latent nonnormality is to assume a different latent distribution, for instance, one with heavier tails. Examples from the SEM literature use elliptical distributions (Yuan et al., 2004) or skew-elliptical distributions (Asparouhov & Muthén, 2016), while Lyhagen & Ornstein (2023), Jin & Yang-Wallentin (2017), and Roscino & Pollice (2006) consider nonnormal distributions specifically in the context of polychoric correlation. Furthermore, it is worth pointing out that the term “robustness” is used in different ways in the methodological literature. Here, it refers to robustness against model misspecification. A popular but different meaning is robustness against heteroskedastic standard errors and corrected goodness-of-fit test statistics (e.g., Li, 2016; Satorra & Bentler, 1994, 2001, and the references therein), which is, for instance, how the popular software package lavaan (Rosseel, 2012) uses the term. We refer to Alfons & Schley (2025) for an overview of the different meanings of “robustness” and a more detailed discussion.

## 3 Polychoric correlation

The polychoric correlation model (Pearson & Pearson, 1922) models the association between two discrete ordinal variables by assuming that an observed pair of responses to two polytomous items is governed by an unobserved discretization process of latent variables that jointly follow a bivariate standard normal distribution. If both items are dichotomous, the polychoric correlation model reduces to the tetrachoric correlation model of Pearson (1901). In the following, we first define the model and review ML estimation thereof and then introduce a robust estimator in the next section.

### 3.1 The polychoric model

For ease of exposition, we restrict our presentation to the bivariate polychoric model. The model naturally generalizes to higher dimensions (see, e.g., Muthén (1984)).

Let there be two ordinal random variables, *X* and *Y*, that take values in the sets 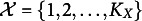 and 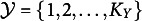 , respectively. The assumption that the sets contain adjacent integers is without loss of generality. Suppose there exist two continuous latent random variables, 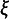 and 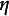 , that govern the ordinal variables through the discretization model 
(3.1)

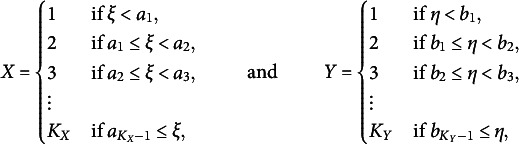

where the fixed but unobserved parameters 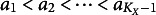 and 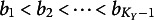 are called *thresholds*.

The primary object of interest is the population correlation between the two latent variables. To identify this quantity from the ordinal variables 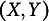 , one assumes that the continuous latent variables follow a standard bivariate normal distribution with unobserved pairwise correlation coefficient 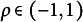 , that is, 
(3.2)

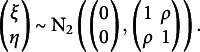

Combining the discretization model (3.1) with the latent normality model (3.2) yields the *polychoric model*. In this model, one refers to the correlation parameter 
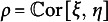
 as the *polychoric correlation coefficient* of the ordinal *X* and *Y*. The polychoric model is subject to 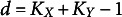 parameters, namely, the polychoric correlation coefficient from the latent normality model (3.2) and the two sets of thresholds from the discretization model (3.1). These parameters are jointly collected in a *d*-dimensional vector 

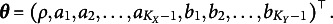



Under the polychoric model, the probability of observing an ordinal response 
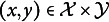
 at a parameter vector 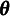 is given by 
(3.3)

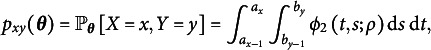

where we use the conventions 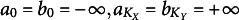 , and 

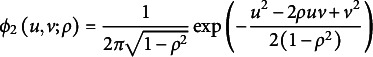

denotes the density of the standard bivariate normal distribution function with correlation parameter 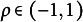 at some 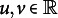 , with corresponding distribution function 

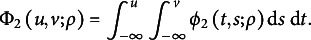



Regarding identification, it is worth mentioning that in the case where both *X* and *Y* are dichotomous, the polychoric model is exactly identified by the standard bivariate normal distribution. If at least one of the ordinal variables has more than two response categories, the polychoric model is over-identified, so it could identify more parameters than those in 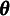 .^3^ We refer to Olsson (1979, Section 2) for a related discussion.

To distinguish arbitrary parameter values 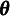 from a specific value under which the polychoric model generates ordinal data, denote the latter by 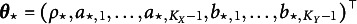 . Given a random sample of ordinal data generated by a polychoric model under parameter value 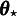 , the statistical problem is to estimate the true 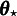 , which is traditionally achieved by the ML estimator (MLE) of Olsson (1979).^4^

### 3.2 Maximum likelihood estimation

Suppose we observe a sample 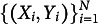 of *N* independent copies of 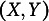 generated by the polychoric model under the true parameter 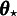 . The sample may be observed directly or as a 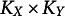 contingency table that cross-tabulates the observed frequencies. Denote by 

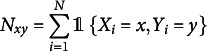

the observed empirical frequency of a response 
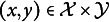
 , where the indicator function 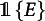 takes value 1 if an event *E* is true, and 0 otherwise. The MLE of 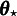 can be expressed as 
(3.4)

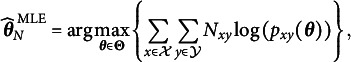

where the 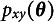 are the response probabilities in (3.3), and 
(3.5)

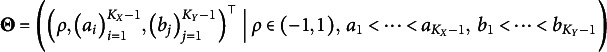

is a set of parameters 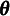 that rules out degenerate cases, such as 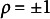 or thresholds that are not strictly monotonically increasing. This estimator, its computational details, as well as its statistical properties are derived in Olsson (1979). In essence, if the polychoric model (3.1) is correctly specified—that is, the underlying latent variables 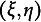 are indeed standard bivariate normal—then the estimator 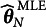 is consistent for the true 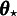 . In addition, 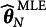 is asymptotically normally distributed with mean zero and covariance matrix being equal to the model’s inverse Fisher information matrix, which makes it fully efficient.

As a computationally attractive alternative to estimating all parameters in 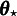 simultaneously in problem (3.4), one may consider a “2-step approach” where only the correlation coefficient 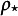 is estimated via ML, but not the thresholds. In this approach, one estimates in a first step the thresholds as quantiles of the univariate standard normal distribution, evaluated at the observed cumulative marginal proportion of each contingency table cell. Formally, in the 2-step approach, thresholds 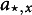 and 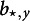 are, respectively, estimated via 
(3.6)

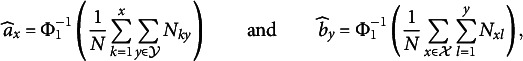

for 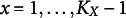 and 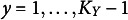 , where 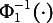 denotes the quantile function of the univariate standard normal distribution. Then, taking these threshold estimates as fixed in the polychoric model, one estimates in a second step the only remaining parameter, correlation coefficient 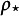 , via ML. The main advantage of the 2-step approach is reduced computational time, while it comes at the cost of being theoretically non-optimal because ML standard errors do not apply to the threshold estimators in (3.6) (Olsson, 1979). Using simulation studies, Olsson (1979) finds that the two approaches tend to yield similar results in practice—both in terms of correlation and variance estimation—for small to moderate true correlations, while there can be small differences for larger true correlations.

Software implementations of polychoric correlation vary with respect to their estimation strategy. For instance, the popular R packages lavaan (Rosseel, 2012) and psych (Revelle, 2024) only support the 2-step approach, while the package polycor (Fox, 2022) supports both the 2-step approach and simultaneous estimation of all model parameters, with the former being the default. Our implementation of ML estimation in package robcat also supports both strategies.

## 4 Conceptualizing model misspecification

To study the effects of partial model misspecification from a theoretical perspective, we first rigorously define this concept and explain how it differs from distributional misspecification.

### 4.1 Partial misspecification of the polychoric model

The polychoric model is partially misspecified when not all unobserved realizations of the latent variables 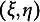 come from a standard bivariate normal distribution. Specifically, we consider a situation where only a fraction 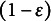 of those realizations are generated by a standard bivariate normal distribution with true correlation parameter 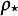 , whereas a fixed but unknown fraction 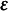 are generated by some different but unspecified distribution *H*. Note that *H* being unspecified allows its correlation coefficient to differ from 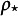 so that realizations generated by *H* may be uninformative for the true polychoric correlation coefficient 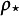 , such as, after discretization, careless responses, misresponses, or responses stemming from item misunderstanding.

Formally, we say that the polychoric model is partially misspecified if the latent variables 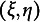 are jointly distributed according to 
(4.1)

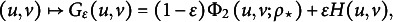

for 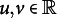 . Conceptualizing model misspecification in such a manner is standard in the robust statistics literature, going back to the seminal work of Huber (1964).^5^ We therefore adopt terminology from robust statistics and call 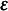 the *contamination fraction*, the uninformative *H* the *contamination distribution* (or simply *contamination*), and 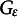 the *contaminated distribution*. Observe that when the contamination fraction is zero, that is, 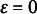 , there is no misspecification so that the polychoric model is correctly specified for all observations. However, neither the contamination fraction 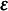 nor the contamination distribution *H* is assumed to be known. Thus, both quantities are left completely unspecified in practice and 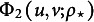 remains the distribution of interest. That is, we only aim to estimate the model parameters 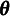 of the polychoric model, while reducing the adverse effects of potential contamination in the observed ordinal data. The contaminated distribution 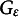 , on the other hand, is never estimated. It serves as a purely theoretical construct that we use to study the theoretical properties of estimators of the polychoric model when that model is partially misspecified due to contamination.

Leaving the contamination distribution *H* and contamination fraction 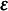 unspecified in the partial misspecification model (4.1) means that we are not making any assumptions on the degree, magnitude, or type of contamination (which is possibly absent altogether). Hence, in our context of responses to rating items, the polychoric model can be misspecified due to an unlimited variety of reasons, for instance, but not limited to careless/inattentive responding (e.g., straightlining, pattern responding, and random-like responding), misresponses, or item misunderstanding.

Although we make no assumption on the specific value of the contamination fraction 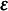 in the partial misspecification model (4.1), we require the identification restriction 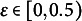 . That is, we require that the polychoric model is correctly specified for the majority of observations, which is standard in the robust statistics literature (e.g., Hampel et al., 1986, p. 67). While it is in principle possible to also consider a contamination fraction between 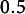 and 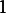 , one would need to impose certain additional assumptions on the correct model to distinguish it from incorrect ones when the majority of observations are not generated by the correct model. Since we prefer refraining from imposing additional assumptions, we only consider 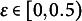 . We discuss the link between identification and contamination fractions beyond 0.5 in more detail in Section E.1 of the Supplementary Material.

Furthermore, as another, more practical reason for considering 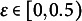 , having more than half of all observations in a sample being not informative for the quantity of interest would be indicative of serious data quality issues. When data quality is unreasonably low, it is doubtful whether the data are suitable for modeling analyses in the first place.

### 4.2 Response probabilities under partial misspecification

Under contaminated distribution 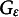 with contamination fraction 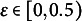 , the probability of observing an ordinal response 
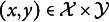
 is given by 
(4.2)

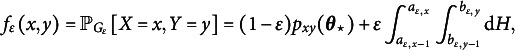

where the unobserved thresholds 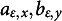 discretize the fraction 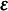 of latent variables for which the polychoric model is misspecified. The thresholds 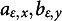 may be different from the true 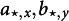 and/or depend on contamination fraction 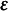 . However, it turns out that from a theoretical perspective, studying the case where the 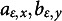 are different from the 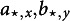 is equivalent to a case where they are equal.^6^

The population response probabilities 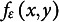 in (4.2) are unknown in practice because they depend on unspecified and unmodeled quantities, namely, the contamination fraction 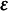 , the contamination distribution *H*, and the discretization thresholds of the latter. Consequently, we do not attempt to estimate the population response probabilities 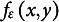 . We instead focus on estimating the true polychoric model probabilities 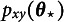 while reducing bias stemming from potential contamination in the observed data.

**Figure 1:**
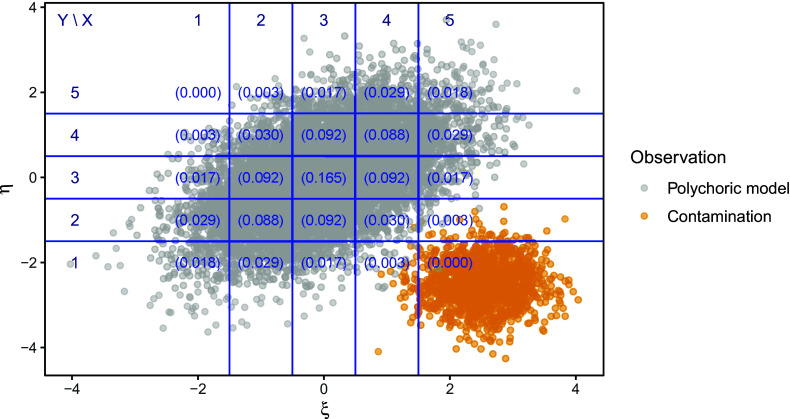
Simulated data with 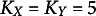 response options where the polychoric model is misspecified with contamination fraction 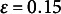 . *Note*: The gray dots represent random draws of 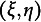 from the polychoric model with 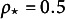 , whereas the orange dots represent draws from a contamination distribution that primarily inflates the cell 
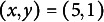
 . The contamination distribution is bivariate normal with a mean 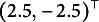 , variances 
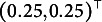
 , and zero correlation. The blue lines indicate the locations of the thresholds. In each cell, the numbers in parentheses denote the population probability of that cell under the true polychoric model.

Figure 1 visualizes a simulated example of bivariate data drawn from contaminated distribution 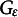 , where a fraction of 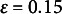 of the data follow a bivariate normal contamination distribution *H* (orange dots) with mean 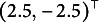 , variance 
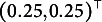
 , and zero correlation, whereas the remaining data are generated by a standard bivariate normal distribution with correlation 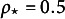 (gray dots). In this example, the data from the contamination distribution *H* primarily inflate the cell 
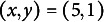
 after discretization. That is, this cell will have a larger empirical frequency than the polychoric model allows for, since the probability of this cell is nearly zero at the polychoric model, yet many realized responses will populate it. Consequently, due to (partial) misspecification of the polychoric model, an ML estimate of 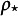 on these data might be substantially biased for 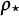 . Indeed, calculating the MLE using the data plotted in Figure 1 yields an estimate of 
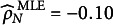
 , which is far off from the true 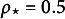 . In contrast, our proposed robust estimator, which is calculated from the exact same information as the MLE and is defined in Section 5, yields a fairly accurate estimate of 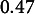 .

It is worth addressing that there exist nonnormal distributions of the latent variables 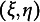 that, after discretization with the same thresholds, result in the same response probabilities as under latent normality (Foldnes & Grønneberg, 2019). This implies that there may exist contamination distributions *H* and contamination fractions 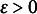 under which the population probabilities 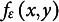 in (4.2) are equal to the true population probabilities of the polychoric model, 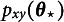 , that is, 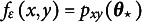 for all 
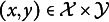
 . In this situation, the polychoric model is misspecified, but the misspecification does not have consequences because the response probabilities remain unaffected. To avoid cumbersome notation in the theoretical analysis of our robust estimator, we assume consequential misspecification throughout this article, that is, 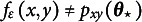 for some 
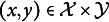
 whenever 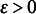 . However, it is silently understood that misspecification need not be consequential, in which case there is no issue and both the MLE and our robust estimator are consistent for the true 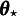 .

### 4.3 Distributional misspecification

A model is distributionally misspecified when all observations in a given sample are generated by a distribution that is different from the model distribution. In the context of the polychoric model, this means that all ordinal observations are generated by a latent distribution that is nonnormal. Let *G* denote the unknown nonnormal distribution that the latent variables 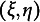 jointly follow under distributional misspecification. The object of interest is the population correlation between latent 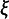 and 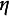 under distribution *G*, for which the normality-based MLE of Olsson (1979) turns out to be substantially biased in many cases (e.g., Foldnes & Grønneberg, 2020, 2022; Jin & Yang-Wallentin, 2017; Lyhagen & Ornstein, 2023). As such, distributional misspecification is fundamentally different from partial misspecification: In the former, one attempts to estimate the population correlation of the nonnormal and unknown distribution that generated a sample, instead of estimating the polychoric correlation coefficient (which is the correlation under standard bivariate normality). In the latter, one attempts to estimate the polychoric correlation coefficient with a contaminated sample that has only been partly generated by latent normality (that is, the polychoric model). The assumption that the polychoric model is only partially misspecified for some uninformative observations enables one to still estimate the polychoric correlation coefficient of that model, which would not be feasible under distributional misspecification (at least not without additional assumptions).

Despite not being designed for distributional misspecification, the robust estimator introduced in the next section can offer a robustness gain in some situations where the polychoric model is distributionally misspecified. We discuss this in more detail in Section 8.

## 5 Robust estimation of polychoric correlation

The behavior of ML estimates of any model crucially depends on the correct specification of that model. Indeed, ML estimation can be severely biased even when the assumed model is only slightly misspecified (e.g., Hampel et al., 1986; Huber, 1964; Huber & Ronchetti, 2009). For instance, in many models of continuous variables like regression models, one single observation from a different distribution can be enough to make the ML estimator converge to an arbitrary value (Huber & Ronchetti, 2009; see also Alfons et al. (2022) for the special case of mediation analysis). The non-robustness of ML estimation of the polychoric model has been demonstrated empirically by Foldnes & Grønneberg (2020, 2022) and Grønneberg & Foldnes (2022) for the case of distributional misspecification. In this section, we introduce an estimator that is designed to be robust to partial misspecification when present, but remains (asymptotically) equivalent to the ML estimator of Olsson (1979) when misspecification is absent. We furthermore derive the statistical properties of the proposed estimator.

Throughout this section, let 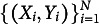 be an observed ordinal sample of size *N* generated by discretizing latent variables 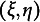 that follow the unknown and unspecified contaminated distribution 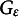 in (4.1). Hence, the polychoric model is possibly misspecified for an unknown fraction 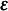 of the sample.

### 5.1 The estimator

The proposed estimator is a special case of a class of robust estimators for general models of categorical data called *C*-estimators (Welz, 2024), and is based on the following idea. The empirical relative frequency of a response 
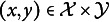
 , denoted 

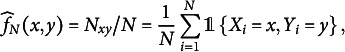

is a consistent nonparametric estimator of the population response probability in (4.2), 

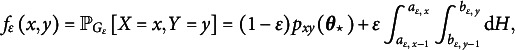

as 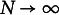 (see, e.g., Chapter 19.2 in Van der Vaart (1998)). If the polychoric model is correctly specified 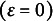 , then 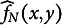 will converge (in probability) to the true model probability 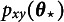 because 

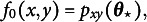

for all 
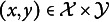
 . Conversely, if the polychoric model is misspecified 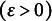 , then 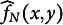 may *not* converge to the true 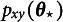 because 

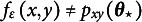

for some 
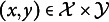
 , since we assume consequential misspecification.

It follows that if the polychoric model is misspecified, there exists no parameter value 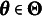 for which the nonparametric estimates 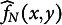 converge pointwise to the associated model probabilities 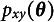 for all 
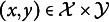
 . Hence, it is indicative of model misspecification if there exists at least one response 
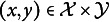
 for which 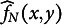 does not converge to any polychoric model probability 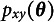 , resulting in a discrepancy between 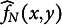 and 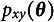 .^7^ This observation can be exploited in model fitting by minimizing the discrepancy between the empirical relative frequencies, 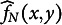 , and theoretical model probabilities, 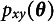 , to find the most accurate fit that can be achieved with the polychoric model for the observed data. Specifically, our estimator minimizes with respect to 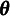 the loss function 
(5.1)

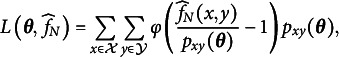

where 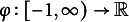 is a prespecified *discrepancy function* that will be defined momentarily. The proposed estimator 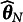 is given by the value minimizing the objective loss over parameter space 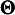 , 
(5.2)

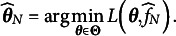

For the choice of discrepancy function 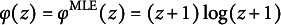 , it can be easily verified that 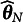 coincides with the MLE 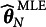 in (3.4). In the following, we motivate a specific choice of discrepancy function 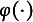 that makes the estimator 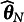 less susceptible to misspecification of the polychoric model while preserving equivalence with ML estimation in the absence of misspecification.

**Figure 2:**
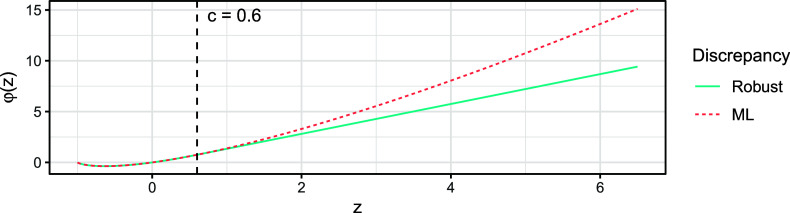
Visualization of the robust discrepancy function 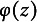 in (5.3) for 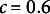 (solid line) and the ML discrepancy function 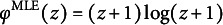 (dotted line).

The fraction between empirical relative frequencies and model probabilities with value 1 deducted, 

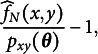

is referred to as *Pearson residual* (PR) (Lindsay, 1994). It takes values in 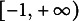 and can be interpreted as a goodness-of-fit measure. PR values close to 0 indicate a good fit between data and polychoric model at 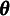 , whereas values toward 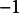 or 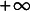 indicate a poor fit because empirical response probabilities disagree with their model counterparts. To achieve robustness to misspecification of the polychoric model, responses that cannot be modeled well by the polychoric model, as indicated by their PR being away from 0, should receive less weight in the estimation procedure such that they do not over-proportionally affect the fit. Downweighting when necessary is achieved through a specific choice of discrepancy function 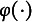 proposed by Welz et al. (2024), which is a special case of a function suggested by Ruckstuhl & Welsh (2001). The discrepancy function reads 
(5.3)

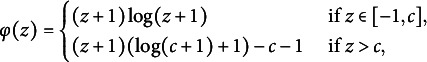

where 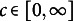 is a prespecified tuning constant that governs the estimator’s behavior at the PR of each possible response. Figure 2 visualizes this function for the example choice 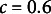 as well as the ML discrepancy function 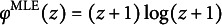 . Note that deducting 1 in (5.1) and adding it again in (5.3) is purely for keeping the interpretation that a PR close to 0 indicates a good fit. We further stress that although the discrepancy function (5.3) can be negative, the loss function (5.1) is always nonnegative (Welz, 2024).

For the choice 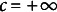 , minimizing the loss (5.1) is equivalent to maximizing the log-likelihood objective in (3.4), meaning that the estimator 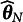 is equal to 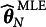 for this choice of *c*. More specifically, if a PR 
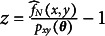
 of a response 
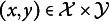
 is such that 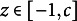 for fixed 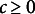 , then the estimation procedure behaves at this response like in classic ML estimation. As argued before, in the absence of misspecification, 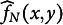 converges to 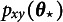 for all responses 
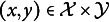
 , therefore, all PRs are asymptotically equal to 0. Hence, if the polychoric model is correctly specified, then estimator 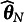 is asymptotically equivalent to the MLE 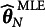 for any tuning constant value 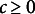 . On the other hand, if a response’s PR *z* is larger than *c*, that is, 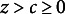 , then the estimation procedure does not behave like in ML, but the response’s contribution to loss (5.1) is linear rather than super-linear like in ML (Figure 2). It follows that the influence of responses that cannot be fitted well by the polychoric model is downweighted to prevent them from dominating the fit. The tuning constant 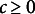 is the threshold beyond which a PR will be downweighted, so the choice thereof determines what is considered an insufficient fit. The closer to 0 the tuning constant *c* is chosen, the more robust the estimator is in theory. In Section C of the Supplementary Material, we explore different values of *c* in simulations and motivate a specific choice that we use for all numerical results in this article, namely, 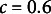 .

Note that the discrepancy function 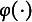 in (5.3) may only downweight *overcounts*, that is, the empirical probability 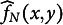 exceeding the theoretical probability 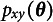 for some cell 
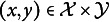
 . One might wonder why *undercounts*— 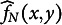 being smaller than 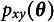 , resulting in negative PRs—are not downweighted as well. Indeed, the discrepancy function in (5.3) does not change its behavior compared to the MLE for PRs below 0. The empirical frequency 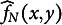 is a *relative* measure, so if a contingency table cell 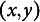 has inflated counts, the other cells will have reduced values of 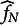 . If the discrepancy function would downweight undercounts, there is a risk of downweighting non-contaminated cells simply because these cells have reduced 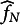 values if at least one cell is inflated due to contamination. Such behavior could result in bias since non-contaminated cells are not supposed to be downweighted. We refer to Ruckstuhl and Welsh (2001, p. 1128) for a related discussion.

With the proposed choice of 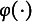 , we stress that our estimator 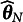 in (5.2) has the same time complexity as ML, namely, 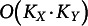 , since one needs to calculate the PR of all 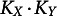 possible responses for every candidate parameter value. Consequently, our proposed estimator does not incur any additional computational cost compared to ML.

### 5.2 Statistical properties

We first address what quantity is estimated by the proposed estimator before discussing its asymptotic behavior.

#### 5.2.1 Estimand

The estimand of the estimator 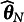 in (5.2) is given by 

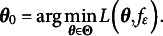

This minimization problem is simply the population analog to the minimization problem in (5.2) that the sample-based 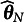 solves because the probabilities 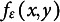 are the population analogs to the empirical probabilities 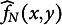 .

If the polychoric model is correctly specified, the estimand 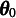 equals the true parameter 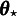 . Indeed, if 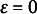 , then 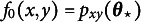 for all 
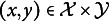
 , so it follows that the loss 

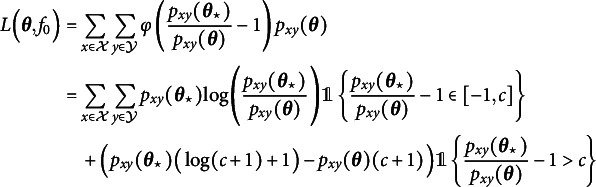

attains its global minimum of zero if and only if 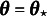 , for any choice of 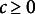 . Thus, in the absence of contamination, our estimator estimates the same quantity as the MLE, namely, the true 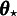 . In other words, it obtains the true 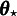 in population when the model is correctly specified, a property known as *Fisher consistency*. We refer to Welz et al. (2024) for details.

However, in the presence of misspecification ( 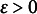 ), the sampling distribution differs from the model distribution such that the estimand 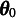 —the parameter that minimizes the loss evaluated at the sampling distribution—is generally different from the true 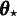 (cf. White, 1982).^8^ The population estimand being different from the true value translates to biased estimates of the latter as a consequence of the misspecification.

**Figure 3:**
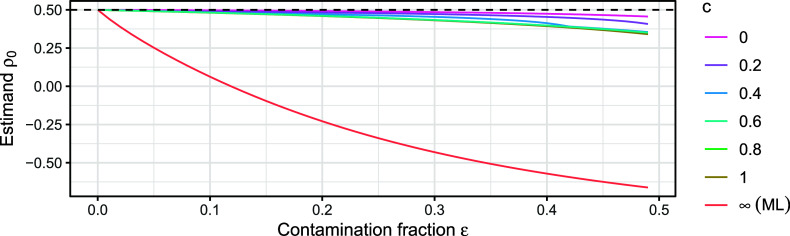
The population estimand 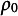 of the polychoric correlation coefficient for various degrees of contamination fractions 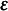 (*x*-axis) and tuning constants *c* (line colors), for the same contamination distribution as in Figure 1. *Note*: The ML estimand corresponds to 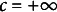 . There are 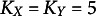 response options and the true value corresponds to 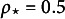 (dashed line).

How much the estimand 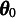 differs from the true 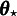 depends on the unknown fraction of contamination 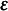 , the unknown type of contamination *H*, as well as the choice of tuning constant *c* in 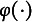 . Mainly, the larger 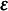 (more severe misspecification) and *c* (less downweighting of hard-to-fit responses), the further 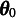 is away from 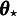 . Figure 3 illustrates this behavior for the polychoric correlation coefficient at an example misspecified distribution that is described in Section 4.2 and in which the true polychoric correlation under the correct model amounts to 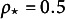 . For increasing contamination fractions, the MLE ( 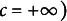 estimates a parameter value that is increasingly farther away from the true 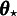 , where already a contamination fraction of less than 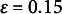 suffices for a sign flip in the correlation coefficient. Conversely, choosing tuning constant *c* to be near 0 results in a much less severe bias. For instance, even at contamination fraction 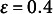 , the difference between the estimand and the true value is approximately 0.1 or less.

Overall, finite choices of *c* lead to an estimator that is at least as accurate as the MLE, and more accurate under misspecification of the polychoric model, thereby gaining robustness to misspecification.

A relevant question is whether the true parameter 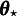 can be recovered when 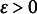 such that it can be estimated using 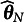 combined with a bias correction term. To derive such a bias correction term, one would need to impose assumptions on the contamination fraction and type of contamination. However, if one has strong prior beliefs about how the polychoric model is misspecified, modeling them explicitly rather than relying on the polychoric model seems more appropriate. Yet, one’s beliefs about misspecification may not be accurate, so attempts to explicitly model the misspecification may themselves result in a misspecified model. Consequently, robust estimation traditionally refrains from making assumptions on how a model may potentially be misspecified by leaving 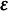 and *H* unspecified in the contaminated distribution (4.1). Instead, one may use a robust estimator to identify data points that cannot be modeled with the model at hand, like the one presented in this article.

#### 5.2.2 Asymptotic analysis

It can be shown that under certain standard regularity conditions that do not restrict the degree or type of possible partial misspecification beyond 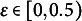 , the robust estimator 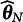 is consistent for estimand 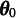 as well as asymptotically normally distributed. Specifically, under said regularity conditions and fixed tuning constant 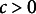 , Theorem A.1 in the Supplementary Material establishes that 

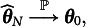

as well as 

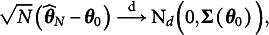

as 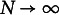 , where “ 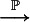 ” and “ 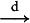 ” denote convergence in probability and distribution, respectively. The asymptotic covariance matrix has a sandwich-type construction 

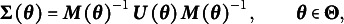

where the 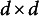 matrices 

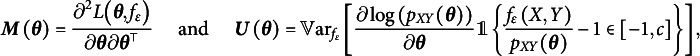

respectively, are the Hessian matrix of the population loss and the covariance matrix (evaluated at 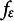 ) of the likelihood score function—that is, the gradient of 
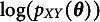
 —weighted by stochastic binary weights whether the PR is smaller than or equal to the tuning constant *c*.^9^ We derive closed-form expressions of the matrices 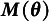 and 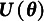 as well as their properties in Section A of the Supplementary Material.

The asymptotic covariance matrix 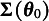 of our estimator is unobserved in practice because it depends on the unknown quantities 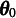 and 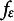 . Yet, 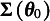 can be consistently estimated by replacing 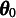 and 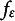 by their corresponding consistent estimators 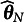 and 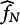 , respectively. Details are provided in Section A of the Supplementary Material.

With this limit theory, one can construct standard errors and confidence intervals for every element in 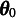 . Importantly, in the absence of contamination (such that 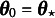 ), the asymptotic covariance matrix 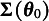 of the robust estimator reduces to that of the fully efficient MLE (i.e., inverse Fisher information matrix) as long as 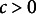 . It follows that there is no loss of statistical efficiency when there is no contamination.^10^ Hence, if the polychoric model is correctly specified, the robust estimator and the MLE are asymptotically first- and second-order equivalent. We refer to Section A of the Supplementary Material for a rigorous exposition and discussion of the robust estimator’s asymptotic properties.

### 5.3 Implementation

We provide a free and open-source implementation of our proposed methodology in a package for the statistical programming environment R (R Core Team, 2024). The package is called robcat (for “ROBust CATegorical data analysis”; Welz et al., 2025), and it is available from CRAN (the Comprehensive R Archive Network) at https://CRAN.R-project.org/package=robcat. To maximize speed and performance, the package is predominantly developed in C++ and integrated to R via Rcpp (Eddelbuettel, 2013). All numerical results in this article were obtained with this package.

The estimator’s minimization problem in (5.2) can be solved with standard algorithms for numerical optimization. In our experience, using an unconstrained version of the quasi-Newton algorithm L-BFGS-B of Byrd et al. (1995) works fine. However, additional stability might be gained from imposing the boundary constraint on the correlation coefficient and the monotonicity constraints on the thresholds, see (3.5), for which the simplex algorithm of Nelder & Mead (1965) for constrained optimization can be used. In our implementation in package robcat, the default behavior is to first try unconstrained optimization via L-BFGS-B. If numerical instability is encountered or a monotonicity constraint is violated, the constrained optimization algorithm of Nelder & Mead (1965) is used instead. While this is the default behavior, the package allows users to freely specify any supported optimization routine.

An important user choice is that of the tuning constant *c* in discrepancy function (5.3). The closer *c* is to 0, the more robust the estimator will be to possible misspecification of the polychoric model (see, e.g., Figure 3). On the other hand, in the presence of model misspecification, the more robust the estimator is made, the larger its estimation variance becomes. Moreover, if the model is correctly specified, then Welz et al. (2024) shows that the most robust choice, 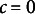 , is associated with two drawbacks, namely, asymptotic nonnormality as well as certain finite sample issues. We therefore suggest choosing a value slightly larger than 0. In simulation experiments (see Section C of the Supplementary Material), we find that the estimator is relatively insensitive to the specific choice of 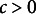 , as long as it is reasonably small (for robustness) yet sufficiently far away from 0 (to avoid the aforementioned issues). The choice 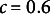 thereby yields a good compromise so that we use this value for all applications in this article. However, we acknowledge that a detailed study, preferably founded in statistical theory, is necessary to provide guidelines on the choice of *c*. We will explore this in future work.

Furthermore, a two-step estimation procedure like in (3.6) is not recommended for robust estimation. The possible presence of responses that have not been generated by the polychoric model can inflate the empirical cumulative marginal proportion of some responses, which may result in a sizable bias of threshold estimates (3.6), possibly translating into biased estimates of polychoric correlation coefficients in the second stage. Our robust estimator therefore estimates all model parameters (thresholds and polychoric correlation) simultaneously.

## 6 Simulation studies on partial misspecification

In this section, we employ two simulation studies to demonstrate the robustness gain of our proposed estimator under partial misspecification of the polychoric model. The first simulation design (Section 6.1) is a simplified setting with respect to the partial misspecification, chosen specifically to illustrate the effects of a particular type of contamination with high leverage affecting only a small number of contingency table cells. The second design (Section 6.2) is more involved and considers estimation of a polychoric correlation matrix with a contamination type that scatters in many directions so that nonnormal data points can occur in every contingency table cell. Section 6.3 summarizes findings from additional simulations in the Supplementary Material. For all simulation designs, we perform 5,000 repetitions.

### 6.1 Individual polychoric correlation coefficient

Let there be 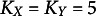 response categories for each of the two rating variables and define the true thresholds in the discretization process (3.1) as 

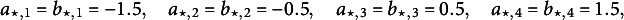

and let the true polychoric correlation coefficient in the latent normality model (3.2) be 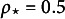 . To simulate partial misspecification of the polychoric model according to (4.1), we let a fraction 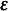 of the data be generated by a particular contamination distribution *H*—which is left unspecified and therefore not explicitly modeled by our estimator—namely, a bivariate normal distribution with mean 
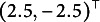
 , variances 
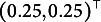
 , and zero covariance (and therefore zero correlation). We discretize the realizations of the contamination distribution according to the same thresholds 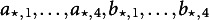 as the uncontaminated realizations. This contamination distribution will inflate the empirical frequency of contingency table cells 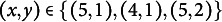 , in the sense that they have a higher realization probability than under the true polychoric model.^11^ In fact, the data plotted in Figure 1 were generated by this process for contamination fraction 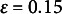 , and one can see in this figure that particularly cell 
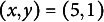
 is sampled frequently although it only has a near-zero probability at the true polychoric model. The data points causing these three cells to be inflated are instances of *negative leverage points*. Here, such leverage points drag correlational estimates away from a positive value toward zero or, if there are sufficiently many of them, even a negative value. For intuition, one may think of such points as the responses of careless or inattentive participants whose responses are not based on item content. Although careless responding is only one special case of the unlimited and unrestricted variety of uninformative responses generated by *H*, we use careless responding as an illustrative running example throughout our simulations.

For contamination fraction 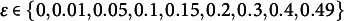 , we sample 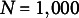 ordinal responses from this data-generating process. We estimate the true parameter 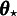 with our proposed estimator with tuning constant set to 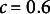 , the MLE (Olsson, 1979), and, for comparison, the Pearson sample correlation calculated on the integer-valued responses.

Let 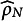 be the estimate on a simulated dataset and 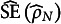 the estimated standard error of 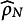 , which is constructed using the limit theory developed in Theorem A.1 in the Supplementary Material. As performance measures, we calculate the average bias of the correlation estimates, the average bias of the standard error estimates (using the standard deviation of the correlation estimates across repetitions as an approximation of the true standard error), as well as coverage and average length of confidence intervals at significance level 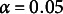 . The coverage is defined as the proportion (across repetitions) of confidence intervals 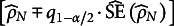 that contain the true 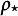 , where 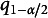 is the 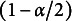 quantile of the standard normal distribution. The length of a confidence interval is given by 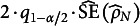 .

**Figure 4:**
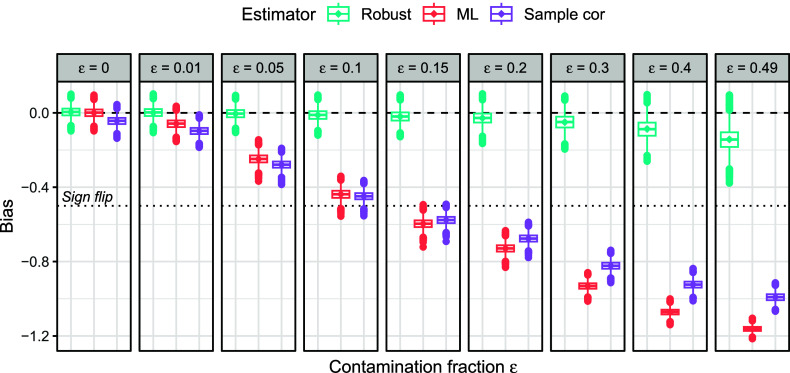
Boxplot visualization of the bias of three estimators of the polychoric correlation coefficient, 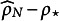 , for various contamination fractions in the misspecified polychoric model across 5,000 repetitions. *Note*: The estimators are the robust estimator with 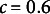 (left), the MLE (center), and the Pearson sample correlation (right). Diamonds represent the respective average bias. The dashed line denotes value 0 and the dotted line 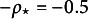 , the latter of which indicating a sign flip in the correlation estimate.

Figure 4 visualizes the bias of each estimator with respect to the true polychoric correlation 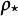 across the 5,000 simulated datasets. An analogous plot for the whole parameter 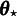 can be found in Section D.1 of the Supplementary Material; the results are similar to those of 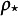 . Additional performance measures are shown in Table 1. For all considered contamination fractions, the estimates of the MLE and sample correlation are somewhat similar, which is expected because these two estimators are known to yield similar results when there are five or more rating options and the discretization thresholds are symmetric (cf. Rhemtulla et al., 2012). In the absence of contamination, the MLE and the robust estimator yield accurate estimates. Both estimators are nearly equivalent to one another in the sense that their point estimates, standard deviation, and coverage at significance level 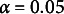 are very similar. However, when contamination is introduced, MLE, sample correlation, and the robust estimator yield noticeably different results. Already at the small contamination fraction 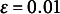 (corresponding to only ten observations), MLE and sample correlation are noticeably biased, resulting in poor coverage of only about 0.45 and 0.04, respectively. Increasing the contamination fraction to the still relatively small value of 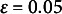 , MLE and sample correlation start to be substantially biased with average biases of 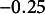 and 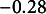 , respectively, leading to zero coverage. The biases of these two methods further deteriorate as the contamination fraction is gradually increased. At 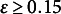 , MLE and sample correlation produce estimates that are not only severely biased but also sign-flipped: while the true correlation is positive (0.5), both estimates are negative. In stark contrast, the proposed robust estimator remains accurate throughout nearly all considered contamination fractions. At the small 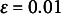 , the robust estimator is nearly unaffected, while at 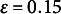 , it only exhibits a minor bias of about 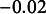 . Even at extreme contamination 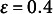 , its bias amounts to less than 0.1. In addition, the coverage of the robust method remains above or close to 0.9 for contamination fractions 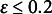 .

**Table 1:** Results for the robust estimator with 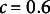 , the MLE, and the Pearson sample correlation, for various contamination fractions across 5,000 simulated datasets

			Point estimate				Standard error			Confidence interval	
Contamination	Estimator		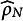	Bias	SE		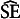	Bias		Coverage	Length
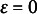	Robust		0.504	0.004	0.027		0.027	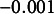		0.931	0.104
	ML		0.500	0.000	0.027		0.026	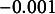		0.939	0.102
	Sample cor		0.456	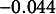	0.025		0.025	0.003		0.690	0.110
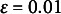	Robust		0.501	0.001	0.028		0.027	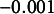		0.938	0.107
	ML		0.441	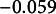	0.027		0.028	0.001		0.446	0.110
	Sample cor		0.402	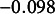	0.025		0.029	0.004		0.043	0.114
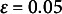	Robust		0.495	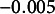	0.029		0.029	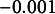		0.939	0.112
	ML		0.252	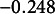	0.028		0.033	0.005		0.000	0.128
	Sample cor		0.221	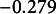	0.025		0.031	0.006		0.000	0.121
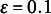	Robust		0.488	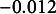	0.031		0.031	0.000		0.933	0.120
	ML		0.062	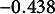	0.028		0.035	0.006		0.000	0.135
	Sample cor		0.052	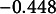	0.025		0.032	0.007		0.000	0.124
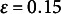	Robust		0.480	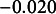	0.033		0.033	0.000		0.907	0.130
	ML		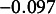	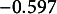	0.027		0.035	0.007		0.000	0.135
	Sample cor		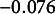	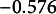	0.025		0.032	0.007		0.000	0.124
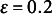	Robust		0.472	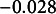	0.036		0.036	0.001		0.882	0.142
	ML		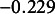	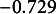	0.026		0.033	0.008		0.000	0.131
	Sample cor		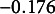	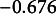	0.025		0.031	0.007		0.000	0.122
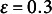	Robust		0.450	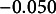	0.043		0.045	0.002		0.789	0.176
	ML		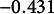	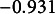	0.022		0.030	0.008		0.000	0.118
	Sample cor		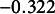	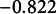	0.024		0.030	0.007		0.000	0.117
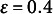	Robust		0.413	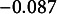	0.053		0.067	0.014		0.595	0.261
	ML		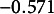	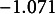	0.019		0.026	0.008		0.000	0.103
	Sample cor		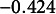	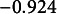	0.024		0.029	0.005		0.000	0.112
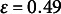	Robust		0.357	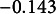	0.062		0.071	0.009		0.247	0.278
	ML		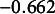	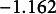	0.016		0.024	0.008		0.000	0.095
	Sample cor		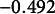	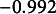	0.023		0.028	0.004		0.000	0.108

*Note*: The true polychoric correlation coefficient is 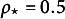 . We compute the average of point estimates 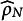 of the polychoric correlation coefficient, the average bias ( 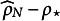 ), the standard deviation of the 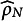 (SE; an approximation of the true standard error), the average standard error estimate 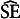 , the corresponding average bias ( 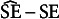 ), confidence interval coverage with respect to the true 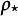 at nominal level 95%, and the average length of the respective confidence intervals.

It is worth noting that the confidence intervals of the robust estimator grow wider with increasing contamination fraction 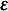 . We also observe that the standard deviation of the robust estimates over the repetitions grows similarly. This indicates that the derived asymptotic distribution used to estimate standard errors matches well with the simulated distribution of the estimator across the repetitions. Indeed, the bias of standard error estimation remains at near-zero for the robust estimator, except for extremely large contamination fractions ( 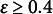 ). We investigate this in more detail in Section D.1 of the Supplementary Material.

### 6.2 Polychoric correlation matrix

The goal of this simulation study is to robustly estimate a polychoric correlation matrix, that is, a matrix comprising of pairwise polychoric correlation coefficients. The simulation design is based on Foldnes & Grønneberg (2020).

Let there be *r* observed ordinal random variables and assume that a latent variable underlies each ordinal variable. In accordance with the multivariate polychoric model (e.g., Muthén, 1984), the latent variables are assumed to jointly follow an *r*-dimensional normal distribution with mean zero and a covariance matrix with unit diagonal elements so that the covariance matrix is a correlation matrix. Each individual latent variable is discretized to its corresponding observed ordinal variable akin to the discretization process (3.1).

Following the five-dimensional simulation design in Foldnes & Grønneberg (2020), there are 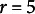 ordinal variables with a polychoric correlation matrix as in Table 2 such that the pairwise correlations vary from a low 0.2 to a moderate 0.56.^12^ For all latent variables, the discretization thresholds are set to, in ascending order, 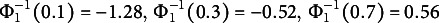 , and 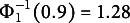 , such that each ordinal variable can take five possible values. A visualization of the implied distribution of each ordinal variable can be found in Figure 5 in Foldnes & Grønneberg (2020).

**Table 2:** Correlation matrix of 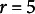 latent variables as in Foldnes & Grønneberg (2020)

Variable	1	2	3	4	5
1	1.00				
2	0.56	1.00			
3	0.48	0.42	1.00		
4	0.40	0.35	0.30	1.00	
5	0.32	0.28	0.24	0.20	1.00

*Note*: In line with the multivariate polychoric correlation model (e.g., Muthén, 1984), the latent variables are jointly normally distributed with mean zero and this correlation matrix as covariance matrix.

**Figure 5:**
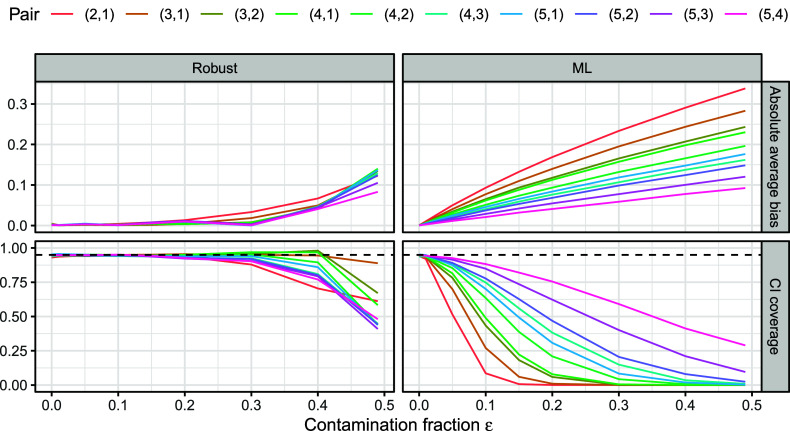
Absolute average bias (top) and confidence interval coverage (bottom) at nominal level 95% (dashed horizontal lines) of the robust estimator with 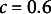 (left) and the MLE (right) for each unique pairwise polychoric correlation coefficient in the true correlation matrix (Table 2), expressed as a function of the contamination fraction 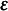 (*x*-axis). *Note*: Results are aggregated over 5,000 repetitions.

As contamination distribution, we choose an *r*-dimensional Gumbel distribution comprising of mutually independent Gumbel marginal distributions, each with location and scale parameters equal to 0 and 3, respectively. To obtain ordinal observations, the unobserved realizations from this distribution are discretized via the same threshold values as realizations from the model (normal) distribution. As such, the uninformative ordinal observations generated by this contaminated distribution emulate the erratic behavior of a careless respondent. Unlike in the previous simulation design (Section 6.1), the uninformative responses are not concentrated around a few response options, but may occur in every response option.

For contamination fraction 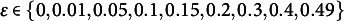 , we sample 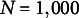 ordinal five-dimensional responses from this data-generating process and use them to estimate the polychoric correlation matrix in Table 2 via our robust estimator (again with tuning constant 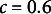 ) as well as the MLE.

Figure 5 visualizes the absolute average bias as well as confidence interval coverage at 95% nominal level (calculated over the 5,000 repetitions) of the robust estimator and the MLE for each pairwise polychoric correlation coefficient. As expected, when the model is correctly specified ( 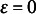 ), both estimators coincide with accurate estimates. However, in the presence of contamination ( 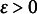 ), the two estimators deviate. The MLE exhibits a notable bias for all correlation coefficients, which increases gradually with increasing contamination fraction. The magnitude of the bias tends to be larger for pairs with larger true correlation, such as 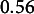 for pair 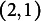 , than for pairs with weaker true correlation, such as 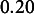 for pair 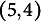 . In addition, coverage of the MLE drops quickly for many pairs and gradually for the remaining ones. Conversely, the robust estimator remains nearly unaffected for a broad range of contamination fractions for each correlation coefficient ( 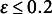 or 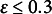 depending on the variable pair), with bias only somewhat increasing afterward. Furthermore, its coverage remains close to 0.9 or higher even at the high contamination fraction of 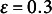 . This reflects the excellent performance of the proposed estimator with respect to robustness to uninformative responses. Section D.2 of the Supplementary Material contains additional evaluations. In essence, the robust estimator yields accurate standard errors, but its confidence intervals tend to be wider than those of the MLE in the presence of contamination.

We stress that polychoric correlation matrices need not be positive definite (e.g., Mair, 2018, p. 22), although all estimated polychoric correlation matrices in this simulation turned out positive definite. If an estimated polychoric correlation matrix is not positive definite, one may opt to apply a smoothing procedure like in Yuan et al. (2011) or Bock et al. (1988).

### 6.3 Discussion and additional simulation experiments

The two simulation studies above demonstrate that already a small degree of partial misspecification due to uninformative responses, such as careless responses, can render the commonly employed MLE unreliable, while the proposed robust estimator retains good accuracy and coverage even in the presence of a considerable number of uninformative responses. On the other hand, when the polychoric model is correctly specified, the MLE and the robust estimator produce equivalent estimates.

To further evaluate our robust estimator and investigate its limitations, we conduct additional simulation experiments in Section E of the Supplementary Material.

The first experiment, described in Section E.2 of the Supplementary Material, is a generalization of the design in Section 6.1 with different *mean shifts* in the contamination distribution *H*. For small mean shifts, the proposed estimator does not improve upon the MLE, but the bias of both estimators remains reasonable. The larger the mean shift, the larger the detrimental effect on the MLE and the higher the robustness gain of our proposed estimator.

The second experiment, described in Section E.3 of the Supplementary Material, focuses on *correlation shifts* in the contamination distribution *H*. Specifically, the contamination distribution is the same as the true model distribution except for a sign-flipped correlation coefficient 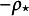 . For moderate correlation 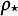 , the proposed estimator does not improve upon the MLE due to substantial overlap between the true model distribution and the contamination. However, the gain in robustness increases substantially for higher correlation coefficients 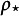 . We expect the gain in robustness to increase for a higher number of response options and decrease for fewer response options. In the most extreme case of two dichotomous rating variables, no improvement can be expected.

## 7 Empirical application

We now demonstrate our proposed method on empirical data by using a subset of the 100 unipolar markers of the Big Five personality traits (Goldberg, 1992).

### 7.1 Background and study design

Each marker is an item comprising a single English adjective (such as “bold” or “timid”) asking respondents to indicate how accurately the adjective describes their personality using a 5-point Likert-type rating scale (*very inaccurate, moderately inaccurate, neither accurate nor inaccurate, moderately accurate*, and *very accurate*). Here, each Big Five personality trait is measured with six pairs of adjectives that are polar opposites to one another (such as “talkative” vs. “silent”), that is, 12 items in total for each trait. It seems implausible that an attentive respondent would choose to agree (or disagree) to *both* items in a pair of polar opposite adjectives. Consequently, one would expect a strongly negative correlation between polar adjectives if all respondents respond attentively (Arias et al., 2020).

Arias et al. (2020) collect measurements of three Big Five traits in this way, namely, *extroversion, neuroticism*, and *conscientiousness*.^13^ The sample that we shall use, Sample 1 in Arias et al. (2020), consists of 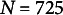 online respondents who are all U.S. citizens, native English speakers, and tend to have relatively high levels of reported education (about 90% report to hold an undergraduate or higher degree). Concerned about respondent inattention in their data, Arias et al. (2020) construct a factor mixture model for detecting inattentive/careless participants. Their model crucially relies on response inconsistencies to polar opposite adjectives and is designed to primarily detect careless straightlining responding. They find that careless responding is a sizable problem in their data. Their model finds evidence of straightliners, and the authors conclude that if unaccounted for, they can substantially deteriorate the fit of theoretical models, produce spurious variance, and overall jeopardize the validity of research results.

Due to the suspected presence of careless respondents, we apply our proposed method to estimate the polychoric correlation coefficients between all 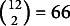 unique item pairs in the *neuroticism* scale to obtain an estimate of the scale’s (polychoric) correlation matrix. The results of the remaining two scales are qualitatively similar and are reported in Section F of the Supplementary Material. We estimate the polychoric correlation matrix via the MLE and via our proposed robust alternative with tuning parameter 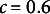 . As a robustness check, we further investigate the effect of the choice of *c*.

### 7.2 Results

**Figure 6:**
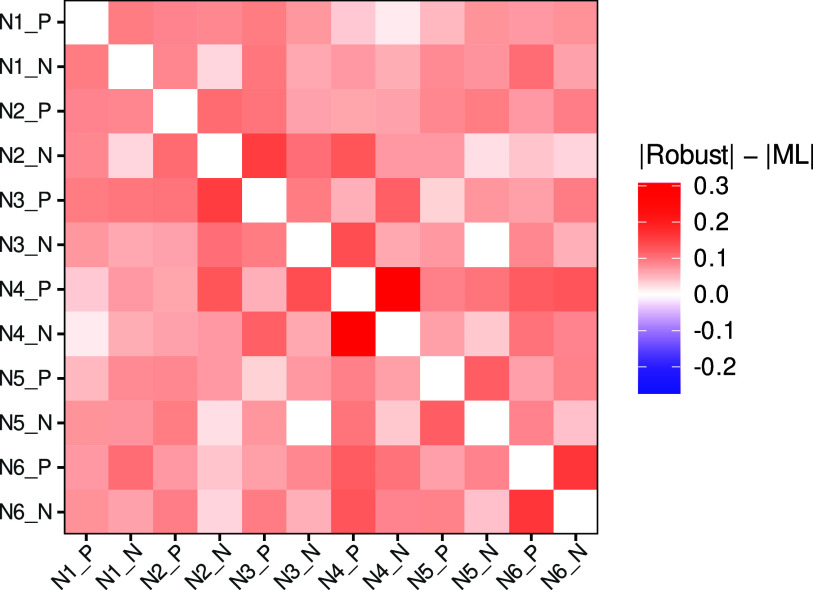
Difference between absolute estimates for the polychoric correlation coefficient of the robust estimator with 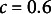 and the MLE for each item pair in the *neuroticism* scale, using the data of Arias et al. (2020). *Note*: The items are “calm” (N1_P), “angry” (N1_N), “relaxed” (N2_P), “tense” (N2_N), “at ease” (N3_P), “nervous” (N3_N), “not envious” (N4_P), “envious” (N4_N), “stable” (N5_P), “unstable” (N5_N), “contented” (N6_P), and “discontented” (N6_N). For the item naming given in parentheses, items with identical identifier (the integer after the first “N”) are polar opposites, where a last character “P” refers to the positive opposite and “N” to the negative opposite. The individual estimates of each method are provided in Table F.2 in the Supplementary Material.

Figure 6 visualizes the difference in absolute estimates for the polychoric correlation coefficient between all 66 unique item pairs in the *neuroticism* scale. For all unique pairs, our method estimates a stronger correlation coefficient than the MLE. The differences in absolute estimates on average amount to 0.083, ranging from only marginally larger than zero to a substantive 0.314. For correlations between polar opposite adjectives, the average absolute difference between our robust method and the MLE is 0.151. The fact that a robust method consistently yields stronger correlation estimates than the MLE, particularly between polar opposite adjectives, is indicative of the presence of leverage points, which drag negative correlation estimates toward zero, that is, they attenuate the estimated strength of correlation. Here, such leverage points could be the responses of careless respondents who report agreement or disagreement to *both* items in item pairs that are designed to be negatively correlated. For instance, recall that it is implausible that an attentive respondent would choose to agree (or disagree) to *both* adjectives in the pair “envious” and “not envious” (cf. Arias et al., 2020). If sufficiently many such respondents are present, then the presumably strongly negative correlation between these two opposite adjectives will be estimated to be weaker than it actually is.

To further investigate the presence of careless respondents who attenuate correlational estimates, we study in detail the adjective pair “not envious” and “envious,” which featured the largest discrepancy between the ML estimate and the robust estimate in Figure 6, with an absolute difference of 0.314. The results of the two estimators and, for completeness, the sample correlation, are summarized in Table 3. The ML estimate of 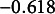 and sample correlation estimate of 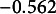 for the (polychoric) correlation coefficient seem remarkably weak considering that the two adjectives in question are polar opposites. In contrast, the robust correlation estimate is given by 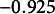 , which seems much more in line with what one would expect if all participants responded accurately and attentively (cf. Arias et al., 2020).

**Table 3:** Parameter estimates and standard error estimates ( 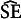 ) for the correlation between the *neuroticism* adjective pair “not envious” and “envious” in the data of Arias et al. (2020), using the robust estimator with 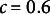 , the MLE, and the Pearson sample correlation

		Robust			ML			Sample cor	
Parameter		Estimate	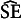		Estimate	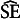		Estimate	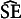
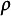		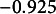	0.062		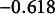	0.025		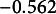	0.031
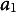		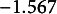	0.276		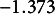	0.061			
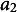		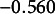	0.203		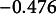	0.043			
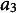		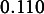	0.187		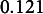	0.042			
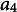		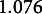	0.105		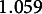	0.054			
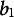		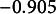	0.073		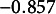	0.049			
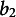		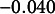	0.091		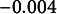	0.041			
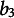		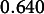	0.364		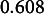	0.044			
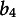		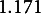	0.811		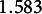	0.071			

*Note*: Each adjective item has five answer categories. Note that the Pearson sample correlation does not model thresholds.

To study the potential presence of careless responses in each contingency table cell 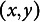 for item pair “not envious” and “envious,” Table 4 lists the PRs at the robust estimate, alongside the associated model probabilities and empirical relative frequencies.^14^ A total of six cells have extremely large PR values of higher than 1,000, and, in addition, five cells have a PR of higher than 9, and one cell has a PR of higher than 4. Such PR values are far away from the ideal value 0 at which the model would fit perfectly, thereby indicating a poor fit of the polychoric model for such responses. It stands out that all such poorly fitted cells are those whose responses might be viewed as inconsistent. Indeed, response cells 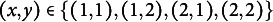 indicate that a participant reports that *neither* “not envious” nor “envious” characterizes them accurately, which are mutually contradicting responses, while for response cells 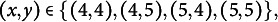 *both* adjectives characterize them accurately, which is again contradicting. As discussed previously, such responses might be due to careless responding. The robust estimator suggests that such responses cannot be fitted well by the polychoric model and subsequently downweighs their influence in the estimation procedure by mapping their PR with the linear part of the discrepancy function 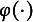 in (5.3). Notably, also cells 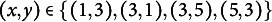 are poorly fitted. These responses report (dis)agreement to one opposite adjective, while being neutral about the other opposite. It is beyond the scope of this article to assess whether such response patterns are also indicative of careless responding, but the robust estimator suggests that such responses at least cannot be fitted well by the polychoric model with the data of Arias et al. (2020).

**Table 4:** Empirical relative frequency (top), estimated response probability (center), and Pearson residual (PR) (bottom) of each response 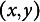 for the item pair “not envious” (*X*) and “envious” (*Y*) in the measurements of Arias et al. (2020) of the *neuroticism* scale

*X*∖*Y*	1	2	3	4	5
1	0.019	0.007	0.003	0.028	0.022
2	0.007	0.040	0.050	0.138	0.014
3	0.006	0.047	0.143	0.030	0.003
4	0.054	0.189	0.029	0.019	0.007
5	0.108	0.018	0.006	0.008	0.007
(a) Empirical relative frequencies 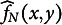					
*X*∖*Y*	1	2	3	4	5
1	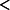 0.001	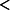 0.001	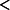 0.001	0.024	0.034
2	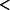 0.001	0.004	0.062	0.153	0.010
3	0.001	0.072	0.145	0.038	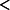 0.001
4	0.061	0.205	0.047	0.002	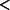 0.001
5	0.120	0.020	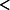 0.001	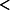 0.001	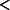 0.001
(b) Estimated response probabilities 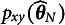					
*X*∖*Y*	1	2	3	4	5
1	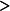 1,000	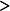 1,000	10.81	0.14	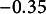
2	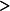 1,000	9.06	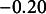	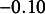	0.42
3	4.48	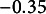	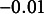	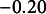	76.11
4	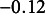	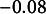	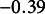	11.66	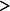 1,000
5	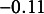	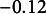	34.98	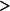 1,000	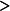 1,000
(c) Pearson residuals 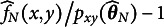					

*Note*: Estimate 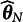 was computed via the robust estimator with tuning constant 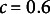 . The complete PR values are provided in Table F.5 in the Supplementary Material.

**Figure 7:**
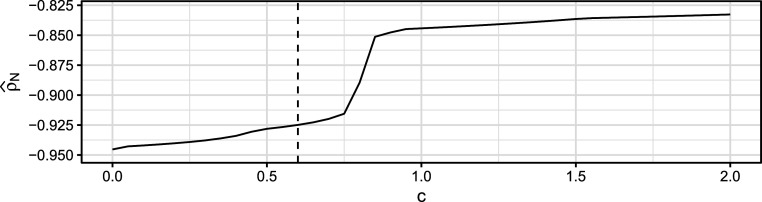
Estimates of the polychoric correlation between the items “not envious” and “envious” in the data of Arias et al. (2020) for various choices of the tuning constant *c* (*x*-axis). *Note*: The dashed vertical line marks the default value of 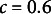 .

As a robustness check on the role of tuning constant *c*, Figure 7 visualizes the point estimate 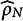 of the polychoric correlation between the item pair “not envious” and “envious” for various values of *c*. The point estimate stays relatively constant for *c* between 0 and 0.75, with 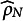 ranging between 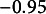 and 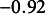 . Just after 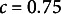 , 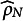 abruptly jumps to about 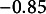 , before it stabilizes again and slowly transitions to the ML estimate of 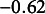 (see Table 3) for 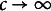 . Since 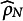 very slowly approaches the value of the ML estimate, we only visualize choices of *c* up to 2 in Figure 7. The instability of the estimate around 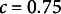 suggests that *c* should be chosen below this value to avoid disproportionate influence of poorly fitting cells. For the broad range of 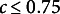 , we obtain a robust finding of “not envious” and “envious” having a very strong negative correlation after accounting for likely careless responding.

Overall, leveraging our robust estimator, we find evidence for the presence of careless respondents in the data of Arias et al. (2020). While they substantially affect the correlation estimate of the MLE, amounting to about 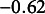 , which is much weaker than one would expect for polar opposite items, our robust estimator can withstand their influence with an estimate of about 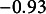 and also help identify them through unreasonably large PR values. On a final note, our findings from the empirical application align remarkably well with those of the simulation from Section E.3 of the Supplementary Material, therefore strengthening their validity. We provide a detailed discussion of this similarity in Section E.3.3 of the Supplementary Material.

## 8 Estimation under distributional misspecification

The simulation studies in Section 6 have been concerned with a situation in which the polychoric model is misspecified for a subset of a sample (partial misspecification). However, as outlined in Section 4.3, another misspecification framework of interest is that of *distributional misspecification* where the model is misspecified for the entire sample. Suppose that instead of a bivariate standard normal distribution, the latent variables 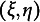 jointly follow an unknown and unspecified distribution *G*. In this framework, the object of interest is the population correlation between 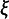 and 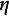 under distribution *G*, that is, 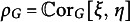 , rather than a polychoric correlation coefficient. Estimators for such situations where the population distribution *G* is nonnormal have been proposed by Lyhagen & Ornstein (2023), Jin & Yang-Wallentin (2017), and Roscino & Pollice (2006).

### 8.1 Distributional vs. partial misspecification

Huber and Ronchetti (2009, p. 4) note that, although conceptually distinct, robustness to distributional misspecification and partial misspecification are *“practically synonymous notions.”* Hence, despite distributional misspecification not being covered by the partial misspecification framework under which we study the theoretical properties of our proposed estimator, our estimator could still offer a gain in robustness compared to the MLE of the polychoric model under distributional misspecification. Specifically, the robust estimator may still be useful if the central part of the nonnormal distribution *G* is not too different from a standard bivariate normal distribution. Intuitively, if the difference between *G* and a standard normal distribution is mainly in the tails, *G* can be approximated by a contaminated distribution as in (4.1), with the standard normal distribution covering the central part and some contamination distribution *H* covering the tails. The polychoric MLE tries to treat influential observations from the tails—which cannot be fitted well by the polychoric model—as if they were normally distributed, resulting in a possibly large estimation bias. In contrast, the robust estimator uses the normal distribution only for observations from the central part—which may fit the polychoric model well enough—and downweights observations from the tails. Thus, as long as such a contaminated normal distribution is a decent approximation of the nonnormal distribution *G*, the robust estimator should perform reasonably well. However, if *G* cannot be approximated by such a contaminated normal distribution, neither the polychoric MLE nor our estimator can be expected to perform well. Overall, though, our estimator could offer an improvement in terms of robustness to distributional misspecification.

In the following, we perform a simulation study to investigate the performance of our estimator when the polychoric model is distributionally misspecified.

### 8.2 Simulation study

To simulate ordinal variables that were generated by a nonnormal latent distribution *G*, we employ the VITA simulation method of Grønneberg & Foldnes (2017). For a pre-specified value of the population correlation 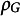 , the VITA method models the latent random vector 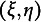 such that the individual variables 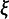 and 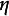 both possess standard normal marginal distributions with population correlation set equal to 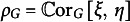 , but are *not* jointly normally distributed. Instead, their joint distribution *G* is equal to a pre-specified nonnormal copula distribution, such as the Clayton or Gumbel copula. Grønneberg & Foldnes (2017) show that discretizing such VITA latent variables yields ordinal observations that could not have been generated by a standard bivariate normal distribution, thereby ensuring proper violation of the latent normality assumption.

**Figure 8:**
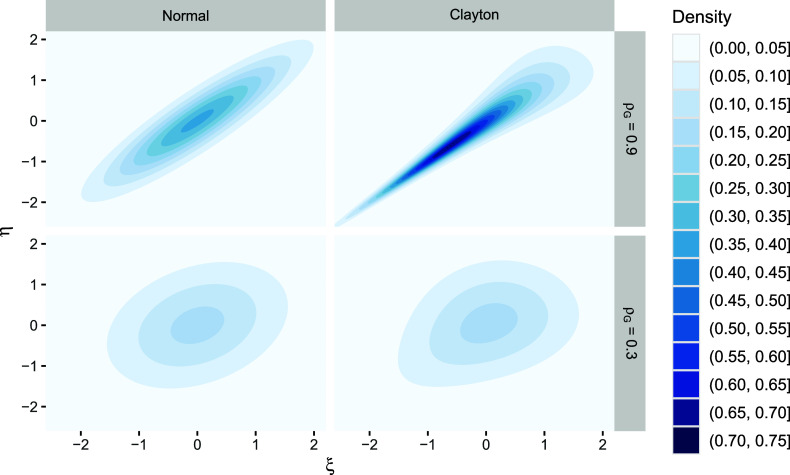
Bivariate density plots of the standard normal distribution (left) and Clayton copula with standard normal marginals (right), for population correlations 0.9 (top) and 0.3 (bottom).

To investigate the robustness of our estimator to distributional misspecification, we use the VITA implementation in package covsim (Grønneberg et al., 2022) to generate draws of the latent variables 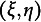 such that the latent variables are jointly distributed according to a Clayton copula *G* with population correlation 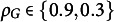 (see Figure 8 for visualizations). Following the discretization process (3.1), we discretize both latent variables via discretization thresholds 

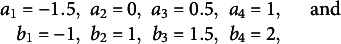

such that both resulting ordinal variables have five response options each. We generate 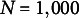 ordinal responses according to this data generation process and compute across 5,000 repetitions the same estimators and performance measures as in the simulations in Section 6.

**Figure 9:**
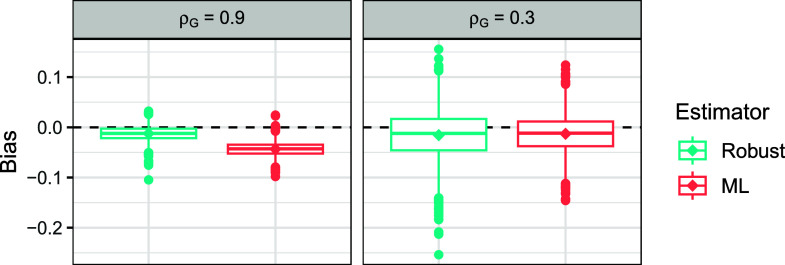
Boxplot visualization of the bias of the robust estimator and the polychoric MLE, 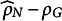 , under distributional misspecification via a Clayton copula with correlation 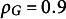 (left) and 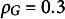 (right), across 5,000 repetitions. *Note*: Diamonds represent the respective average bias. The tuning constant of the robust estimator is set to 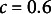 .

Figure 9 visualizes the bias of the robust estimator and the polychoric MLE under both Clayton copulas across the repetitions.^15^ For correlation 0.9, the polychoric MLE exhibits a noteworthy bias, whereas the robust estimator remains accurate, albeit with a larger estimation variance as compared to most simulation configurations of partial misspecification (cf. Section 6). Conversely, for the weaker correlation 0.3, both estimators are fairly accurate with average biases of about 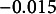 . Table 5 contains additional performance measures regarding inference. For 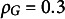 , the average standard error estimate of the robust method is accurate, but for 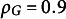 , it notably overestimates. We therefore also computed the median of the standard error estimates in the latter case: at 0.017, it is fairly close to the true standard error of 0.014. It turns out that there is a small number of simulated datasets with a large majority of empty cells in the contingency table, resulting in numerical instability of the standard error estimates and inflating their average. As discussed in Section 4.3, distributional misspecification is not covered by our partial misspecification framework, so it is not surprising that standard errors derived under partial misspecification are not always valid. Bootstrap inference may therefore be an attractive alternative. Nevertheless, unlike the polychoric MLE with coverage of only about 13% for 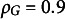 , the robust estimator maintains high coverage of over 90%.

**Table 5:** Results for the robust estimator with 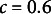 and the polychoric MLE across 5,000 simulated datasets under distributional misspecification via a Clayton copula with true population correlation 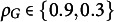

			Point estimate				Standard error			Confidence interval	
Correlation	Estimator		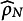	Bias	SE		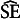	Bias		Coverage	Length
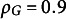	Robust		0.888	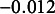	0.014		0.037	0.023		0.915	0.146
	ML		0.857	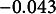	0.013		0.016	0.002		0.132	0.061
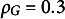	Robust		0.284	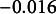	0.047		0.052	0.006		0.938	0.205
	ML		0.287	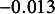	0.036		0.036	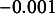		0.932	0.141

*Note*: See Table 1 for explanatory notes on the performance measures.

These results suggest that at least for point estimation, the Clayton copula with correlation 0.9 might be reasonably approximable by a contaminated normal distribution where the normal distribution covers the center of the probability mass and some contamination distribution covers the tails (cf. Section 8.1). Indeed, Figure 8 indicates that the normal distribution and Clayton copula at correlation 0.9 seem to behave similarly in the center, but deviate from one another toward the tails. On the other hand, at correlation 0.3, the two densities do not appear to be drastically different from one another, which may explain why *both* the polychoric MLE and the robust estimator work reasonably well for such distributional misspecification. If the latent distribution is not too different from a normal distribution, then the polychoric model may offer a satisfactory fit despite being technically misspecified.

Overall, this simulation study demonstrates that in some cases of distributional misspecification, robustness can be gained with our estimator, compared to the polychoric MLE. However, it also demonstrates that there are cases of distributional misspecification for which the polychoric MLE still works quite well such that the robust estimator offers little gain. Nevertheless, the fact that in some situations robustness can be gained under distributional misspecification represents an overall gain in robustness.

## 9 Discussion and conclusion

We consider a situation where the polychoric correlation model is potentially misspecified for a subset of responses in a sample, that is, a set of uninformative observations not generated by a latent standard normal distribution. This model misspecification framework, called *partial misspecification* here, stems from the robust statistics literature, and a relevant special case is that of careless respondents in questionnaire studies. We demonstrate that ML estimation is highly susceptible to the presence of such uninformative responses, resulting in possibly large estimation biases and low coverage of confidence intervals.

As a remedy, we propose an estimator based on the *C*-estimation framework of Welz et al. (2024) that is designed to be robust to partial model misspecification. Our estimator generalizes ML estimation, does not make any assumption on the magnitude or type of potential misspecification, comes at no additional computational cost, and possesses attractive statistical guarantees, such as asymptotic normality. It furthermore allows to pinpoint the sources of potential model misspecification through the notion of PRs. Each possible response option is assigned a PR, where values substantially larger than the ideal value 0 imply that the response in question cannot be fitted well by the polychoric correlation model. In addition, the methodology proposed in this article is implemented in the free open-source package robcat (Welz et al., 2025) for the statistical programming environment R and is publicly available at https://CRAN.R-project.org/package=robcat.

Although not covered by our partial misspecification framework, we also discuss how and when our estimator can offer a robustness gain (compared to the polychoric MLE) when the polychoric model is misspecified for *all* observations, which has been a subject of interest in recent literature. In essence, there can be a robustness gain if the latent nonnormal distribution that generated the data can be reasonably well approximated by a contaminated normal distribution where the normal distribution reflects the central part and some unspecified contamination distribution reflects the tails.

We verify the enhanced robustness and theoretical properties of our robust estimator in simulation studies. Furthermore, we demonstrate the estimator’s practical usefulness in an empirical application on a Big Five administration, where we find compelling evidence for the presence of careless respondents as a source of partial model misspecification.

However, our estimator depends on a user-specified choice of a tuning constant *c*, which governs a tradeoff between robustness and efficiency in case of misspecification. While simulation experiments suggest that the choice 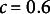 provides a good tradeoff and estimates do not change considerably for a broad range of finite choices of *c*, a detailed investigation on this tuning constant needs to be carried out in future work. As an alternative, one could consider other discrepancy functions that do not depend on tuning constants, like the ones discussed in Welz et al. (2024). As practical guidelines, we recommend always comparing robust estimates to that of ML and running the robust estimator for various choices of *c*, like in Figure 7. Doing so not only helps assess the estimates’ stability, but also the severity of (partial) model misspecification. If the ML and robust estimates strongly differ, one may want to opt for choices of 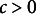 not too far from 0 to achieve larger robustness gains.

A practical consideration of polychoric correlation is computing time. Our estimator simultaneously estimates all model parameters (i.e., correlation coefficient and thresholds) for robustness reasons, hence, it is computationally more intensive than the non-robust two-stage approach of Olsson (1979). To alleviate the computational burden, our implementation in package robcat is written in fast and efficient C++ code. Furthermore, its default behavior first tries a fast unconstrained numerical optimization routine, which in our experience almost always suffices and executes in about half a second for five-point rating variables on a regular laptop. For estimating polychoric correlation matrices, package robcat supports parallel computing to keep computation time low. Since our method generalizes ML and has the same time complexity, these functionalities also provide a fast implementation of the MLE.

The methodology proposed in this article suggests a number of extensions. For instance, one could use a robustly estimated polychoric correlation matrix in SEMs to robustify such models and their fit indices against misspecification. Similar robustification could be achieved in, for instance, but not limited to, principal component analyses, multidimensional scaling, or clustering. In addition, the theory of Welz et al. (2024) may allow to pinpoint possible sources of model misspecification on the individual response level. That is, it may enable the derivation of statistically sound cutoff values for PRs in order to detect whether a given response can be fitted well by the polychoric correlation model. We leave these avenues to further research.

Overall, our novel robust estimator could open the door for a new line of research that is concerned with making the correlation-based analysis of rating data more reliable by reducing dependence on modeling assumptions.

## Supporting information

10.1017/psy.2025.10066.sm001 Welz et al. supplementary material Welz et al. supplementary material

## Data Availability

Replication files are publicly available on GitHub at https://github.com/mwelz/robust-polycor-replication.

## Footnotes

1 A key reason for this finding is that the VM method produces a latent vector whose distribution corresponds either exactly or near-exactly to a Gaussian copula (Foldnes & Grønneberg, 2015) (except in regions where certain polynomials used in the VM transformation are non-monotonous). It follows that the VM transformation *“inherits a Gaussian-like property, indicating that simulation studies based on the VM approach might give overly optimistic impressions of finite-sample properties of estimators with non-Gaussian data”* (Foldnes & Grønneberg, 2015, p. 1078). To simulate non-normality, one should instead strive for a copula with distinctively non-Gaussian features, such as tail dependence and asymmetry. This is exactly what an alternative transformation method proposed by Grønneberg & Foldnes (2017) does. For instance, generating data with the tail-dependent and tail-asymmetric Clayton copula leads to markedly different behavior of normality-based estimators than with the VM method (e.g., Foldnes & Grønneberg, 2022).

2 Another slightly more subtle difference is that Itaya & Hayashi (2025) robustify *marginal* maximum ML, whereas we robustify *joint* ML estimation. Marginal ML is often used to estimate IRT models because joint ML is known to yield inconsistent parameter estimates in such models (e.g., Lindsay et al., 1991). The polychoric correlation model does not suffer from such problems and can therefore be estimated via joint ML. Furthermore, marginal ML in IRT is a *full information estimation* concept, whereas multivariate models that are being fitted to a given polychoric correlation matrix require *limited information estimation*. Hence, the work of Itaya & Hayashi (2025) demonstrates that model misspecification is also a relevant issue in full information estimation.

3 An ordinal sample provides  statistics, namely, the observed frequencies for each response category  . Since the sum of the individual response frequencies must equal the sample size, one loses one degree of freedom so that only  of the frequency statistics are independent. Subsequently, the ordinal sample can identify at most  parameters. In particular, when *X* and *Y* are both dichotomous, only three parameters can be identified. In this case, the polychoric model (which reduces to the tetrachoric model here) depends on three parameters, namely, a correlation parameter and two threshold parameters.

4 Alternatives to the commonly used MLE of Olsson (1979) have been proposed in the literature (see, for instance, Zhang et al. (2024), Jöreskog (1994), and Lau (1985)).

5 In the robust statistics literature, a distribution like (4.1) is known as *Huber contamination model*. For continuous random variables, this model is primarily used to model outliers and study the properties of outlier-robust estimators.

6 Let  be an arbitrary contamination distribution of the latent variables. Let the thresholds that discretize these latent variables be given by arbitrary values  , for  . Since one makes no assumption on the contamination distribution in (4.1), we can find another contamination distribution  that, when discretized with the true thresholds  , yields the same discretization as  with thresholds  . Formally,  .

7 Actually, if said convergence fails, it does so for at least two responses. Due to the relative frequencies summing up to 1, if the convergence fails for one response, it must also fail for at least one more.

8 White (1982) is concerned with (quasi-)ML estimation, but the same reasoning applies to our estimator. In the context of White (1982), the ML estimand of a misspecified model corresponds to the parameter value minimizing the Kullback–Leibler divergence between the sampling density and the model density. For  (the ML case), the population analog of our loss function (5.1) corresponds to the Kullback–Leibler divergence between the sampling density  and the model density  , that is,  . For finite choices of *c*, our loss function is designed to yield an estimand that is closer to the true  than that of the ML loss by downweighting responses that cannot be sufficiently well modeled.

9 It is worth mentioning that for the MLE (  ), our sandwich-type covariance matrix  reduces to that of White (1982) and Huber (1967), who studied the limit distribution of ML in misspecified models.

10 This property does not contradict standard theory on the Cramér–Rao lower bound (CRLB) for the variance of unbiased estimators (e.g., Theorem 7.3.9 in Casella & Berger, 2002). In brief, if a statistical model is correctly specified, the variance of the MLE asymptotically attains this lower bound such that no unbiased estimator can be asymptotically more efficient than the MLE. Nevertheless, this does not imply that no other estimators can (asymptotically) attain the CRLB. When the polychoric model is correctly specified (i.e., no contamination), our estimator has the same asymptotic variance as the MLE, which satisfies the CRLB with equality.

11 Conceptually, contamination in the cell  would correspond to a straightlining careless respondent in  or  if one of the items is negatively keyed. Thus, after recoding, cell  will be inflated.

12 The correlation matrix in Table 2 has the additional interpretation of being the covariance matrix of a factor model for a single factor with loadings vector  .

13 Arias et al. (2020) synonymously refer to *neuroticism* as *emotional stability*. Furthermore, in addition to the three listed traits, Arias et al. (2020) collect measurements of the trait *dispositional optimism* by using a different instrument, and another scale that is designed not to measure any construct. We do not consider these scales in this empirical demonstration. Furthermore, Arias et al. (2020) have made their data publicly available at https://osf.io/n6krb.

14 A visualization of Table 4 is provided in Figure F.4 in the Supplementary Material.

15 In 32 of the 5,000 repetitions for the Clayton copula, the robust estimator experienced numerical instability and subsequently did not converge to a solution. The polychoric MLE failed to converge 56 times. Consequently, these unconverged estimates are omitted from Figure 9. We explain this numerical issue in detail in Section B of the Supplementary Material. Note that due to the shape of the Clayton copula with high correlation, stability issues with the robust estimator are in our experience amplified if the true thresholds are not well distributed over the domain of the distribution.

## Supplementary material

The supplementary material for this article can be found at https://doi.org/10.1017/psy.2025.10066.
