# Supplementary material for: Robust Estimation of Polychoric Correlation
Source: Psychometrika. 2025 Dec 17;91(1):247–78. doi: 10.1017/psy.2025.10066 (PMC13121829; doi:10.1017/psy.2025.10066)
Supplement: Welz et al. supplementary material [file S0033312325100665sup001.pdf]

Psychometrika (2025), **xx**:xx 1–34

CAMBRIDGE
UNIVERSITY PRESS

ONLINE SUPPLEMENT

Online Supplementary Materials to "Robust Estimation of Polychoric Correlation"

Max Welz[1,3], Patrick Mair[2] and Andreas Alfons[1]

[1]Department of Econometrics, Erasmus University Rotterdam, Rotterdam, 3062 PA, South Holland, The Netherlands. E-mail: alfons@ese.eur.nl.
[2]Department of Psychology, Harvard University, Cambridge, MA 02138, Massachusetts, USA. E-mail: mair@fas.harvard.edu.
[3]Department of Psychology, University of Zurich, Zurich, CH-8050, Zurich, Switzerland. E-mail: max.welz@uzh.ch.

Abstract

This online supplement to the paper "Robust Estimation of Polychoric Correlation" (Welz et al., 2025) contains six sections. Section A provides details on the asymptotic properties of the proposed estimator. Section B is a remark on numerical optimization. Section C discusses the tuning constant c. Section D contains additional results of simulations from the main text. Section E provides additional simulation studies. Section F contains additional results from the empirical application. Unless stated otherwise, all references to labels that start with an Arabic numeral, such as (1.1), refer to labels in the main paper. Conversely, all references to labels starting with a Roman letter, such as (A.1), refer to labels in this online supplement.

A. Asymptotic properties of the robust estimator

This section presents the limit theory of the proposed robust estimator $\widehat{\boldsymbol{\theta}}_N$ in (5.2). Throughout this section, we assume that the number of response categories, K_X and K_Y, are fixed and known, and that the sample $\{(X_i, Y_i)\}_{i=1}^N$ used to compute an estimate $\widehat{\boldsymbol{\theta}}_N$ has been generated by the process in (3.1) where the latent $\{(\xi_i, \eta_i)\}_{i=1}^N$ are draws from distribution G_ε in (4.1) with unobserved contamination fraction $\varepsilon \in [0, 0.5)$ and unknown contamination distribution H.

The following three subsections first state the main theorem, then discuss its assumptions, and then provide closed-form expressions of quantities used in the asymptotic analysis.

A.1. Main theorem

We start by introducing additional notation. Denote the likelihood score function, i.e., the d-dimensional gradient vector of $\log\left(p_{xy}(\boldsymbol{\theta})\right)$ for response $(x, y) \in \mathcal{X} \times \mathcal{Y}$ at parameter $\boldsymbol{\theta} \in \boldsymbol{\Theta}$, by

$$\boldsymbol{s}_{xy}(\boldsymbol{\theta}) = \frac{\partial}{\partial \boldsymbol{\theta}} \log\left(p_{xy}(\boldsymbol{\theta})\right) = \frac{1}{p_{xy}(\boldsymbol{\theta})}\left(\frac{\partial}{\partial \boldsymbol{\theta}}\, p_{xy}(\boldsymbol{\theta})\right).$$

Further, given the tuning constant $c \in [0, \infty]$, define the $d \times d$ matrix

$$\boldsymbol{Q}_{xy}(\boldsymbol{\theta}) = \left(\frac{f_\varepsilon(x,y)}{p_{xy}(\boldsymbol{\theta})} \mathbb{1}\left\{\frac{f_\varepsilon(x,y)}{p_{xy}(\boldsymbol{\theta})} - 1 \in [-1, c]\right\} + (c+1)\mathbb{1}\left\{\frac{f_\varepsilon(x,y)}{p_{xy}(\boldsymbol{\theta})} - 1 \ > c\right\}\right) \frac{\partial^2}{\partial \boldsymbol{\theta} \partial \boldsymbol{\theta}^\top} p_{xy}(\boldsymbol{\theta})$$

for $(x, y) \in \mathcal{X} \times \mathcal{Y}$ and $\boldsymbol{\theta} \in \boldsymbol{\Theta}$. We derive closed-form expressions of the gradient and Hessian matrix of $p_{xy}(\boldsymbol{\theta})$ in Subsection A.2. In addition, for $K = K_X \cdot K_Y$ the total number of contingency table cells and d-dimensional vectors

$$\boldsymbol{w}_{xy}(\boldsymbol{\theta}) = \boldsymbol{s}_{xy}(\boldsymbol{\theta}) \mathbb{1}\left\{\frac{f_\varepsilon(x,y)}{p_{xy}(\boldsymbol{\theta})} - 1 \in [-1, c]\right\},$$

define the $d \times K$ matrix

$$\boldsymbol{W}(\boldsymbol{\theta}) = \left(\boldsymbol{w}_{11}(\boldsymbol{\theta}), \cdots, \boldsymbol{w}_{1,K_Y}(\boldsymbol{\theta}), \boldsymbol{w}_{21}(\boldsymbol{\theta}), \cdots, \boldsymbol{w}_{2,K_Y}(\boldsymbol{\theta}), \cdots, \boldsymbol{w}_{K_X,1}(\boldsymbol{\theta}), \boldsymbol{w}_{K_X,2}(\boldsymbol{\theta}), \cdots, \boldsymbol{w}_{K_X,K_Y}(\boldsymbol{\theta})\right)$$

that row-binds all K vectors $\boldsymbol{s}_{xy}(\boldsymbol{\theta})$ multiplied by an indicator that takes value 1 when associated population Pearson residual is in the MLE-part of the function $\varphi(\cdot)$ in (5.3) and 0 otherwise. In similar fashion, define the K-dimensional vector

$$\boldsymbol{f}_\varepsilon = \left(f_\varepsilon(1,1), \ldots, f_\varepsilon(1,K_Y), f_\varepsilon(2,1), \ldots, f_\varepsilon(2,K_Y), \ldots, f_\varepsilon(K_X,1), f_\varepsilon(K_X,2), \ldots, f_\varepsilon(K_X,K_Y)\right)^\top$$

that holds all K evaluations of the function f_ε, and put

$$\boldsymbol{\Omega} = \operatorname{diag}(\boldsymbol{f}_\varepsilon) - \boldsymbol{f}_\varepsilon \boldsymbol{f}_\varepsilon^\top,$$

where $\operatorname{diag}(\boldsymbol{f}_\varepsilon)$ is a $K \times K$ diagonal matrix that holds the coordinates of $\boldsymbol{f}_\varepsilon$ on its principal diagonal.

The following theorem establishes consistency and asymptotic normality of the estimator. This theorem follows immediately from Theorems 1 and 2 in Welz (2024).

Theorem A.1. *For $c \in [0, \infty]$ the tuning constant in the discrepancy function, assume that $\frac{f_\varepsilon(x,y)}{p_{xy}(\boldsymbol{\theta}_0)} - 1 \neq c$ for all $(x, y) \in \mathcal{X} \times \mathcal{Y}$. Then, under certain regularity conditions that do not restrict the degree or type of possible misspecification of the polychoric model beyond $\varepsilon \in [0, 0.5)$, when $N \longrightarrow \infty$ it holds true that*

$$\widehat{\boldsymbol{\theta}}_N \xrightarrow{\mathbb{P}} \boldsymbol{\theta}_0,$$

as well as

$$\sqrt{N}\left(\widehat{\boldsymbol{\theta}}_N - \boldsymbol{\theta}_0\right) \xrightarrow{\mathrm{d}} \mathrm{N}_d\left(\mathbf{0}, \boldsymbol{\Sigma}\left(\boldsymbol{\theta}_0\right)\right),$$

where, as a function of $\boldsymbol{\theta} \in \boldsymbol{\Theta}$, the estimator's invertible asymptotic covariance matrix is given by

$$\boldsymbol{\Sigma}\left(\boldsymbol{\theta}\right) = \boldsymbol{M}\left(\boldsymbol{\theta}\right)^{-1} \boldsymbol{U}\left(\boldsymbol{\theta}\right) \boldsymbol{M}\left(\boldsymbol{\theta}\right)^{-1},$$

where

$$\begin{aligned} \boldsymbol{U}\left(\boldsymbol{\theta}\right) &= \mathbb{V}\mathrm{ar}_{f_\varepsilon}\left[\boldsymbol{w}_{XY}(\boldsymbol{\theta})\right] = \boldsymbol{W}\left(\boldsymbol{\theta}\right) \boldsymbol{\Omega} \boldsymbol{W}\left(\boldsymbol{\theta}\right)^\top \qquad \textit{and} \\ \boldsymbol{M}\left(\boldsymbol{\theta}\right) &= \frac{\partial^2}{\partial \boldsymbol{\theta} \partial \boldsymbol{\theta}^\top} L\left(\boldsymbol{\theta}, f_\varepsilon\right) \\ &= \sum_{x \in \mathcal{X}} \sum_{y \in \mathcal{Y}} \left[\mathbb{1}\left\{ \frac{f_\varepsilon\left(x, y\right)}{p_{xy}(\boldsymbol{\theta})} - 1 \in [-1, c] \right\} f_\varepsilon\left(x, y\right) \boldsymbol{s}_{xy}(\boldsymbol{\theta}) \boldsymbol{s}_{xy}(\boldsymbol{\theta})^\top - \boldsymbol{Q}_{xy}(\boldsymbol{\theta}) \right] \end{aligned}$$

are $d \times d$ symmetric and invertible matrices.

The regularity conditions are presented and discussed in Welz (2024). We stress again that no assumption is made that would restrict the degree or type of potential misspecification of the polychoric model.

It can be shown that in the absence of misspecification (such that $\boldsymbol{\theta}_0 = \boldsymbol{\theta}_*$), the asymptotic covariance matrix $\boldsymbol{\Sigma}\left(\boldsymbol{\theta}_*\right)$ is equal to the inverted Fisher information matrix of the polychoric model (Lemma B.5 in Welz, 2024), which is well-known to be the asymptotic covariance matrix of the MLE. It follows that under correct specification of the polychoric model, the proposed robust estimator is indeed asymptotically first and second order equivalent to the consistent and efficient MLE of Olsson (1979).

It can furthermore be shown that Theorem A.1 generalizes well-known results on the behavior of ML estimation in misspecified models. Indeed, for the MLE ($c = +\infty$ in the discrepancy function), the sandwich-type asymptotic covariance matrix $\boldsymbol{\Sigma}\left(\boldsymbol{\theta}_0\right)$ reduces to that of White (1982, Theorem 3.2) and Huber (1967, Corollary on p. 231). However, our asymptotic results are more general because they encompass a broad class of estimators, including and beyond ML.

A consistent estimator of the unobserved asymptotic covariance matrix $\boldsymbol{\Sigma}\left(\boldsymbol{\theta}_0\right)$ can be constructed as follows. Replace all population class probabilities $f_\varepsilon\left(x, y\right)$ by their corresponding empirical counterparts $\widehat{f}_N(x, y)$ in matrices $\boldsymbol{W}\left(\boldsymbol{\theta}\right), \boldsymbol{M}\left(\boldsymbol{\theta}\right)$, and $\boldsymbol{\Omega}$, and denote the resulting observable matrices by $\widehat{\boldsymbol{W}}_N\left(\boldsymbol{\theta}\right), \widehat{\boldsymbol{M}}_N\left(\boldsymbol{\theta}\right)$, and $\widehat{\boldsymbol{\Omega}}_N$, respectively. Each of these observable matrices is a (pointwise) consistent estimator of its corresponding population counterpart by the continuous mapping theorem. Then apply the formulas in Theorem A.1 to construct

$$\widehat{\boldsymbol{\Sigma}}_N\left(\boldsymbol{\theta}\right) = \widehat{\boldsymbol{M}}_N\left(\boldsymbol{\theta}\right)^{-1} \widehat{\boldsymbol{U}}_N\left(\boldsymbol{\theta}\right) \widehat{\boldsymbol{M}}_N\left(\boldsymbol{\theta}\right)^{-1},$$

where $\widehat{\boldsymbol{U}}_N(\boldsymbol{\theta}) = \widehat{\boldsymbol{W}}_N(\boldsymbol{\theta})\widehat{\boldsymbol{\Omega}}_N\widehat{\boldsymbol{W}}_N(\boldsymbol{\theta})^\top$. The observable matrix $\widehat{\boldsymbol{\Sigma}}_N(\boldsymbol{\theta})$ is a pointwise consistent estimator of $\boldsymbol{\Sigma}(\boldsymbol{\theta})$ for a given parameter value $\boldsymbol{\theta} \in \boldsymbol{\Theta}$. It now follows from the fact that $\widehat{\boldsymbol{\theta}}_N \xrightarrow{\mathbb{P}} \boldsymbol{\theta}_0$ (Theorem A.1) and the continuous mapping theorem that $\widehat{\boldsymbol{\Sigma}}_N\left(\widehat{\boldsymbol{\theta}}_N\right)$ is consistent for the population asymptotic covariance matrix $\boldsymbol{\Sigma}(\boldsymbol{\theta}_0)$.

For the choice $c = 0$, note that the condition $\frac{f_\varepsilon(x,y)}{p_{xy}(\boldsymbol{\theta}_0)} - 1 \neq c$ in Theorem A.1 rules out the zero-misspecification case ($\varepsilon = 0$). In fact, in this case, the condition is not met for any $(x, y) \in \mathcal{X} \times \mathcal{Y}$. Indeed, Welz (2024) shows that for $c = 0$, the estimator is *not* asymptotically Gaussian if the model is correctly specified. We therefore recommend to choose $c > 0$ (see Section 5.3 of the main text for a discussion). The reason why we require $\frac{f_\varepsilon(x,y)}{p_{xy}(\boldsymbol{\theta}_0)} - 1 \neq c$ for all $(x, y) \in \mathcal{X} \times \mathcal{Y}$ is because the value c is the threshold at which the discrepancy function (5.3) transitions from superlinear growth to linear growth. The discrepancy function is not twice differentiable at this point, which causes a particular Taylor expansion in the theorem's proof to fail to exist. This Taylor expansion plays a crucial role in establishing asymptotic normality. If it cannot be performed for at least one response $(x, y) \in \mathcal{X} \times \mathcal{Y}$, the estimator is not asymptotically normal. We refer to Welz (2024) for a detailed discussion.

A.2. Expressions of first and second order derivatives

This section presents closed-form expressions of all components of the gradient and Hessian matrix of the probability mass function of the polychoric model, $p_{xy}(\boldsymbol{\theta})$.

A.2.1. First order terms

For response $(x, y) \in \mathcal{X} \times \mathcal{Y}$ and $\boldsymbol{\theta} \in \boldsymbol{\Theta}$, the gradient of $p_{xy}(\boldsymbol{\theta})$ can be expressed as

$$\frac{\partial p_{xy}(\boldsymbol{\theta})}{\partial \boldsymbol{\theta}} = \frac{\partial}{\partial \boldsymbol{\theta}}\Phi_2\left(a_x, b_y; \rho\right) - \frac{\partial}{\partial \boldsymbol{\theta}}\Phi_2\left(a_{x-1}, b_y; \rho\right) - \frac{\partial}{\partial \boldsymbol{\theta}}\Phi_2\left(a_x, b_{y-1}; \rho\right) + \frac{\partial}{\partial \boldsymbol{\theta}}\Phi_2\left(a_{x-1}, b_{y-1}; \rho\right), \tag{A.1}$$

see, e.g., Olsson (1979, Equation 4). To characterize this gradient, we provide expressions for individual partial derivatives of $p_{xy}(\boldsymbol{\theta})$, that is,

$$\frac{\partial p_{xy}(\boldsymbol{\theta})}{\partial \boldsymbol{\theta}} = \left(\frac{\partial p_{xy}(\boldsymbol{\theta})}{\partial \rho}, \frac{\partial p_{xy}(\boldsymbol{\theta})}{\partial a_1}, \dots, \frac{\partial p_{xy}(\boldsymbol{\theta})}{\partial a_{K_X-1}}, \frac{\partial p_{xy}(\boldsymbol{\theta})}{\partial b_1}, \dots, \frac{\partial p_{xy}(\boldsymbol{\theta})}{\partial b_{K_Y-1}}\right)^\top.$$

First, for any $u, v \in \mathbb{R}$, it can be shown (e.g., Drezner & Wesolowsky, 1990) that

$$\frac{\partial}{\partial \rho}\Phi_2(u, v; \rho) = \phi_2(u, v; \rho),$$

as well as (e.g., Tallis, 1962)

$$\frac{\partial}{\partial u}\Phi_2(u, v; \rho) = \phi_1(u)\,\Phi_1\left(\frac{v - \rho u}{\sqrt{1-\rho^2}}\right),$$

where $\phi_1(\cdot)$ and $\Phi_1(\cdot)$ denote the density and distribution function, respectively, of the *univariate* standard normal distribution. The complementary partial derivative with respect to v follows analogously by symmetry.

It now follows immediately from (A.1) that the partial derivative of $p_{xy}(\boldsymbol{\theta})$ with respect to ρ is given by

$$\frac{\partial p_{xy}(\boldsymbol{\theta})}{\partial \rho} = \phi_2\left(a_x, b_y; \rho\right) - \phi_2\left(a_{x-1}, b_y; \rho\right) - \phi_2\left(a_x, b_{y-1}; \rho\right) + \phi_2\left(a_{x-1}, b_{y-1}; \rho\right),$$

whereas the partial derivatives with respect to the individual thresholds are characterized by

$$\frac{\partial p_{xy}(\boldsymbol{\theta})}{\partial a_k} = \begin{cases} \frac{\partial}{\partial a_x}\Phi_2\left(a_x, b_y; \rho\right) - \frac{\partial}{\partial a_x}\Phi_2\left(a_x, b_{y-1}; \rho\right) & \text{if } k = x, \\ -\frac{\partial}{\partial a_{x-1}}\Phi_2\left(a_{x-1}, b_y; \rho\right) + \frac{\partial}{\partial a_{x-1}}\Phi_2\left(a_{x-1}, b_{y-1}; \rho\right) & \text{if } k = x-1, \\ 0 & \text{otherwise,} \end{cases}$$

for $k = 1, \ldots, K_X - 1$. An expression for $\frac{\partial p_{xy}(\boldsymbol{\theta})}{\partial b_k}$ can be derived analogously.

A.2.2. Second order terms

Here we provide expressions for the individual coordinates of the $d \times d$ symmetric Hessian matrix of $p_{xy}(\boldsymbol{\theta})$, that is,

$$\frac{\partial^2 p_{xy}(\boldsymbol{\theta})}{\partial \boldsymbol{\theta} \partial \boldsymbol{\theta}^\top} = \begin{pmatrix} \frac{\partial^2 p_{xy}(\boldsymbol{\theta})}{\partial \rho^2} & \frac{\partial^2 p_{xy}(\boldsymbol{\theta})}{\partial \rho \partial a_1} & \cdots & \frac{\partial^2 p_{xy}(\boldsymbol{\theta})}{\partial \rho \partial a_{K_X-1}} & \frac{\partial^2 p_{xy}(\boldsymbol{\theta})}{\partial \rho \partial b_1} & \cdots & \frac{\partial^2 p_{xy}(\boldsymbol{\theta})}{\partial \rho \partial b_{K_Y-1}} \\ \frac{\partial^2 p_{xy}(\boldsymbol{\theta})}{\partial a_1 \partial \rho} & \frac{\partial^2 p_{xy}(\boldsymbol{\theta})}{\partial a_1{}^2} & \cdots & \frac{\partial^2 p_{xy}(\boldsymbol{\theta})}{\partial a_1 \partial a_{K_X-1}} & \frac{\partial^2 p_{xy}(\boldsymbol{\theta})}{\partial a_1 \partial b_1} & \cdots & \frac{\partial^2 p_{xy}(\boldsymbol{\theta})}{\partial a_1 \partial b_{K_Y-1}} \\ \vdots & \vdots & \ddots & \vdots & \vdots & \ddots & \vdots \\ \frac{\partial^2 p_{xy}(\boldsymbol{\theta})}{\partial a_{K_X-1} \partial \rho} & \frac{\partial^2 p_{xy}(\boldsymbol{\theta})}{\partial a_{K_X-1} \partial a_1} & \cdots & \frac{\partial^2 p_{xy}(\boldsymbol{\theta})}{\partial a_{K_X-1}{}^2} & \frac{\partial^2 p_{xy}(\boldsymbol{\theta})}{\partial a_{K_X-1} \partial b_1} & \cdots & \frac{\partial^2 p_{xy}(\boldsymbol{\theta})}{\partial a_{K_X-1} \partial b_{K_Y-1}} \\ \frac{\partial^2 p_{xy}(\boldsymbol{\theta})}{\partial b_1 \partial \rho} & \frac{\partial^2 p_{xy}(\boldsymbol{\theta})}{\partial b_1 \partial a_1} & \cdots & \frac{\partial^2 p_{xy}(\boldsymbol{\theta})}{\partial b_1 \partial a_{K_X-1}} & \frac{\partial^2 p_{xy}(\boldsymbol{\theta})}{\partial b_1{}^2} & \cdots & \frac{\partial^2 p_{xy}(\boldsymbol{\theta})}{\partial b_1 \partial b_{K_Y-1}} \\ \vdots & \vdots & \ddots & \vdots & \vdots & \ddots & \vdots \\ \frac{\partial^2 p_{xy}(\boldsymbol{\theta})}{\partial b_{K_Y-1} \partial \rho} & \frac{\partial^2 p_{xy}(\boldsymbol{\theta})}{\partial b_{K_Y-1} \partial a_1} & \cdots & \frac{\partial^2 p_{xy}(\boldsymbol{\theta})}{\partial b_{K_Y-1} \partial a_{K_X-1}} & \frac{\partial^2 p_{xy}(\boldsymbol{\theta})}{\partial b_{K_Y-1} \partial b_1} & \cdots & \frac{\partial^2 p_{xy}(\boldsymbol{\theta})}{\partial b_{K_Y-1}{}^2} \end{pmatrix}.$$

This Hessian matrix can alternatively be expressed as follows, which follows by (A.1):

$$\frac{\partial^2 p_{xy}(\boldsymbol{\theta})}{\partial \boldsymbol{\theta} \partial \boldsymbol{\theta}^\top} = \frac{\partial^2}{\partial \boldsymbol{\theta} \partial \boldsymbol{\theta}^\top}\Phi_2\left(a_x, b_y; \rho\right) - \frac{\partial^2}{\partial \boldsymbol{\theta} \partial \boldsymbol{\theta}^\top}\Phi_2\left(a_{x-1}, b_y; \rho\right) - \frac{\partial^2}{\partial \boldsymbol{\theta} \partial \boldsymbol{\theta}^\top}\Phi_2\left(a_x, b_{y-1}; \rho\right) + \frac{\partial^2}{\partial \boldsymbol{\theta} \partial \boldsymbol{\theta}^\top}\Phi_2\left(a_{x-1}, b_{y-1}; \rho\right). \tag{A.2}$$

First, by means of repeated applications of the product rule and chain rule it can be shown that for any $u, v \in \mathbb{R}$,

$$\frac{\partial^2}{\partial \rho^2}\Phi_2\left(u, v; \rho\right) = \frac{\partial}{\partial \rho}\phi_2\left(u, v; \rho\right) = \frac{\phi_2\left(u, v; \rho\right)}{(1-\rho^2)^2}\Big((1-\rho^2)(\rho + uv) - \rho\big(u^2 - 2\rho uv + v^2\big)\Big),$$

as well as

$$\frac{\partial^2}{\partial u^2}\Phi_2\left(u,v;\rho\right)=\phi_1'(u)\Phi_1\left(\frac{v-\rho u}{\sqrt{1-\rho^2}}\right)-\frac{\rho}{\sqrt{1-\rho^2}}\phi_1\left(u\right)\phi_1\left(\frac{v-\rho u}{\sqrt{1-\rho^2}}\right),$$

where

$$\phi_1'(u)=-\frac{u}{\sqrt{2\pi}}\exp\left(-u^2/2\right),$$

which follows immediately by the chain rule.

Next, for the second order cross-derivatives, it can be shown that

$$\frac{\partial^2}{\partial u\partial\rho}\Phi_2\left(u,v;\rho\right)=\phi_1\left(u\right)\phi_1\left(\frac{v-\rho u}{\sqrt{1-\rho^2}}\right)\frac{\rho v-u}{(1-\rho^2)^{3/2}}$$

and

$$\frac{\partial^2}{\partial u\partial v}\Phi_2\left(u,v;\rho\right)=\phi_1\left(u\right)\phi_1\left(\frac{v-\rho u}{\sqrt{1-\rho^2}}\right)\frac{1}{\sqrt{1-\rho^2}},$$

both by applications of the chain rule and product rule.

It now follows by (A.2) combined with these second order cross-derivatives that

$$\frac{\partial^2 p_{xy}(\boldsymbol{\theta})}{\partial a_k\partial\rho}=\begin{cases}\frac{\partial^2}{\partial a_x\partial\rho}\Phi_2\left(a_x,b_y;\rho\right)-\frac{\partial^2}{\partial a_x\partial\rho}\Phi_2\left(a_x,b_{y-1};\rho\right) & \text{if } k=x,\\ -\frac{\partial^2}{\partial a_{x-1}\partial\rho}\Phi_2\left(a_{x-1},b_y;\rho\right)+\frac{\partial^2}{\partial a_{x-1}\partial\rho}\Phi_2\left(a_{x-1},b_{y-1};\rho\right) & \text{if } k=x-1,\\ 0 & \text{otherwise,}\end{cases}$$

and

$$\frac{\partial^2 p_{xy}(\boldsymbol{\theta})}{\partial a_k\partial b_l}=\begin{cases}\frac{\partial^2}{\partial a_k\partial b_l}\Phi_2\left(a_x,b_y;\rho\right) & \text{if } (k,l)\in\left\{(x,y),(x-1,y-1)\right\},\\ -\frac{\partial^2}{\partial a_k\partial b_l}\Phi_2\left(a_x,b_y;\rho\right) & \text{if } (k,l)\in\left\{(x-1,y),(x,y-1)\right\},\\ 0 & \text{otherwise,}\end{cases}$$

and

$$\frac{\partial^2 p_{xy}(\boldsymbol{\theta})}{\partial a_k\partial a_l}=\begin{cases}\frac{\partial^2 p_{xy}(\boldsymbol{\theta})}{\partial {a_k}^2} & \text{if } k=l,\\ 0 & \text{otherwise,}\end{cases}$$

and

$$\frac{\partial^2 p_{xy}(\boldsymbol{\theta})}{\partial {a_k}^2}=\begin{cases}\frac{\partial^2}{\partial {a_x}^2}\Phi_2\left(a_x,b_y;\rho\right)-\frac{\partial^2}{\partial {a_x}^2}\Phi_2\left(a_x,b_{y-1};\rho\right) & \text{if } k=x,\\ -\frac{\partial^2}{\partial {a_{x-1}}^2}\Phi_2\left(a_{x-1},b_y;\rho\right)+\frac{\partial^2}{\partial {a_{x-1}}^2}\Phi_2\left(a_{x-1},b_{y-1};\rho\right) & \text{if } k=x-1,\\ 0 & \text{otherwise,}\end{cases}$$

and, finally,

$$\frac{\partial^2 p_{xy}(\boldsymbol{\theta})}{\partial \rho^2} = \frac{\partial^2}{\partial \rho^2}\Phi_2\left(a_x, b_y; \rho\right) - \frac{\partial^2}{\partial \rho^2}\Phi_2\left(a_{x-1}, b_y; \rho\right) - \frac{\partial^2}{\partial \rho^2}\Phi_2\left(a_x, b_{y-1}; \rho\right) + \frac{\partial^2}{\partial \rho^2}\Phi_2\left(a_{x-1}, b_{y-1}; \rho\right).$$

B. Remark on numerical optimization

In the simulation study on distributional misspecification from Section 8 of the main text, the robust estimator did not converge to a solution in 32 out of 5,000 repetitions. This issue occurred 27 times for the Clayton copula with correlation 0.9, and five times with correlation 0.3. The polychoric MLE failed to converge 56 times, all at the Clayton copula with correlation 0.9. For the robust estimator, this situation tends to occur in the rare event that single rows or columns in a contingency table contain only one non-zero cell. It turns out that for such data, the estimator may attempt to effectively eliminate a threshold by either pushing the outermost finite thresholds to $\pm\infty$ or pulling adjacent thresholds to be as close to each other as numerically feasible, thereby practically merging these thresholds into one. Such behavior often leads to degeneracy of the final Nelder-Mead simplex (if used). Thus, our package `robcat` identifies numerical instability either by degeneracy of Nelder-Mead simplexes and/or adjacent thresholds being unreasonably far away from each other, and subsequently throws a warning. We decided to set the cutoff for two adjacent thresholds being unreasonably far away to a minimum distance of 3.92. Under the polychoric model, this distance can cover as much as 95% of all probability mass of the corresponding standard normal marginal distribution. We believe that having adjacent threshold values separated by this much probability mass may be an indication of poor model fit and/or severe measurement issues. Hence, being able to detect such numerical instability may in fact be viewed as a useful feature of our robust estimator: it alerts the user to potential broader issues where the use of the model may not be recommended for the data at hand.

C. The tuning constant c

C.1. Background

Our proposed discrepancy function $\varphi(\cdot)$ in (5.3) depends on the choice of a tuning constant $c \geq 0$. By construction of the discrepancy function, the larger the choice of c, the more similar the robust estimate will become to the ML estimate (which occurs for $c = +\infty$): as one gradually increases c from 0 to larger values, the corresponding estimate gradually approaches the ML estimate (see, e.g., Figures 3 and 7 in the main text). This behavior begs the question which value of c one should choose. This section motivates and explains the reasoning behind the choice of $c = 0.6$, which we use throughout this paper.

In theory, the closer c is chosen to 0, the more robust the estimator becomes against contamination because cells whose Pearson residuals exceed the ideal value 0 will be downweighted more stringently. On the other hand, if contamination is absent, Theorem A.1 reveals that any choice of c that is *strictly* larger than 0 will result in an asymptotically fully efficient estimator. Therefore, from a purely theoretical perspective, one should choose a c that infinitesimally exceeds 0.

Needless to say, asymptotic theory and empirical practice are two very different animals: In practice, one only has access to a finite number of observations, N, so a number of finite sample issues might arise that could affect practical recommendations for the choice of c. Therefore, this section carries out simulation studies to explore the effects of different choices of c.

C.2. Simulation design

To explore the effects of various choices of c, we use the same data generating process as in the simulation design in Section 6.1 of the main text, in which we are interested in the polychoric correlation between two ordinal variables (X, Y) with $K_X = K_Y = 5$ response categories. The true value of the polychoric correlation coefficient is set to $\rho_* \in \{0, 0.5\}$, which by construction corresponds to the population correlation between the latent (ξ, η) under the polychoric model. The latent variables are then discretized according to the true thresholds

$$a_{*,1} = b_{*,1} = -1.5, \quad a_{*,2} = b_{*,2} = -0.5, \quad a_{*,3} = b_{*,3} = 0.5, \quad a_{*,4} = b_{*,4} = 1.5.$$

To simulate contamination, we let a fraction $\varepsilon \in \{0, 0.1, 0.2\}$ of the data be generated by a contamination distribution H, which here is a bivariate normal distribution with population mean $(2.5, -2.5)^\top$, variances $(0.25, 0.25)^\top$, and zero correlation between the two latent variables. We then discretize the latent realizations from this contamination distribution according to the thresholds $(a_{*,j}, b_{*,j}, j = 1, \dots, 4)$. We sample $N = 1,000$ ordinal observations $(X_i, Y_i)_{i=1}^N$ from this data generating process. We then use this sample to estimate the polychoric correlation between the two ordinal variables with our proposed robust estimator at various choices of tuning parameter c. Specifically, the set of considered tuning parameters c is given by the granular grid $\{0, 0.1, 0.2, \dots, 14.8, 14.9, 15, +\infty\}$, where $c = +\infty$ is understood as the maximum likelihood estimator (MLE). We repeat this procedure for $T = 5,000$ simulation runs.

C.3. Performance measures

Let $\widehat{\rho}_N^{(t)}$ denote the point estimate of the t-th simulation repetition, $t = 1, \dots, T$, where $T = 5,000$. For performance evaluation, we compute the following three statistics.

1. Sample mean of the 5,000 point estimates:

$$\widehat{\rho}_N^{\text{ave}} = \frac{1}{T}\sum_{t=1}^{T}\widehat{\rho}_N^{(t)},$$

being a performance measure for accuracy in the estimation of the true ρ_*. When contamination is present ($\varepsilon > 0$), we expect that the further c is away from 0, the larger the estimation error becomes. Conversely, in the absence of contamination ($\varepsilon = 0$), the estimates should be accurate no matter the choice of c.

2. Sample mean of the 5,000 standard error estimates associated with each point estimate:

$$\widehat{\text{SE}}^{\text{ave}}\left(\widehat{\rho}_N\right) = \frac{1}{T}\sum_{t=1}^{T}\widehat{\text{SE}}\left(\widehat{\rho}_N^{(t)}\right),$$

capturing dispersion estimation. Every individual standard error estimate $\widehat{\text{SE}}\left(\widehat{\rho}_N^{(t)}\right)$ estimates the true standard error $\text{SE}\left(\widehat{\rho}_N\right)$, hence also $\widehat{\text{SE}}^{\text{ave}}\left(\widehat{\rho}_N\right)$ estimates $\text{SE}\left(\widehat{\rho}_N\right)$. To approximate the estimand $\text{SE}\left(\widehat{\rho}_N\right)$ we calculate the standard deviation of the simulated distribution of point estimates as the third statistic.

3. Sample standard deviation of the 5,000 point estimates:

$$\text{SE}^{\text{approx}}\left(\widehat{\rho}_N\right) = \sqrt{\frac{1}{T-1}\sum_{t=1}^{T}\left(\widehat{\rho}_N^{(t)} - \widehat{\rho}_N^{\text{ave}}\right)^2}.$$

We call this statistic $\text{SE}^{\text{approx}}\left(\widehat{\rho}_N\right)$ because it approximates the finite-sample standard error $\text{SE}\left(\widehat{\rho}_N\right)$. Although an asymptotic expression for the standard error is known from Theorem A.1 (the square root of first diagonal element of $\boldsymbol{\Sigma}\left(\boldsymbol{\theta}_0\right)$), the true standard error $\text{SE}\left(\widehat{\rho}_N\right)$ in finite samples is unknown. We therefore use the standard deviation of the simulated sampling distribution as an approximation.

Comparing $\widehat{\text{SE}}^{\text{ave}}\left(\widehat{\rho}_N\right)$ with $\text{SE}^{\text{approx}}\left(\widehat{\rho}_N\right)$ can be seen as a sanity check for the correctness of the estimator's limit theory in Theorem A.1. Hence, if the limit theory is correct, we expect for a sufficiently large sample size that $\widehat{\text{SE}}^{\text{ave}}\left(\widehat{\rho}_N\right)$ will be close to $\text{SE}^{\text{approx}}\left(\widehat{\rho}_N\right)$. However, recall from Theorem A.1 that the estimator is *not* asymptotically Gaussian for the choice $c = 0$ when contamination is absent ($\varepsilon = 0$). Thus, in this case ($c = \varepsilon = 0$), we expect $\widehat{\text{SE}}^{\text{ave}}\left(\widehat{\rho}_N\right)$ to differ from $\text{SE}^{\text{approx}}\left(\widehat{\rho}_N\right)$ because the former is derived under the then-invalid asymptotic normality of estimator $\widehat{\boldsymbol{\theta}}_N$.

C.4. Simulation results

Figure C.1 visualizes the results as a function of the tuning constant c for the considered contamination fractions ε (columns) and true correlation coefficients ρ_* (rows). The average point estimate $\widehat{\rho}_N^{\text{ave}}$ is represented by a solid black line, while a dashed blue line indicates the true ρ_*. In addition, the average standard error estimate $\widehat{\text{SE}}^{\text{ave}}\left(\widehat{\rho}_N\right)$ and the approximate true standard error $\text{SE}^{\text{approx}}\left(\widehat{\rho}_N\right)$ are visualized by confidence bands: a shaded gray area covers $\widehat{\rho}_N \pm q_{1-\alpha/2}\cdot\widehat{\text{SE}}^{\text{ave}}\left(\widehat{\rho}_N\right)$ and dotted blue lines indicate $\widehat{\rho}_N \pm q_{1-\alpha/2}\cdot\text{SE}^{\text{approx}}\left(\widehat{\rho}_N\right)$, where $q_{1-\alpha/2}$ denotes the $(1-\alpha/2)$ quantile of the standard normal distribution for $\alpha = 0.05$.

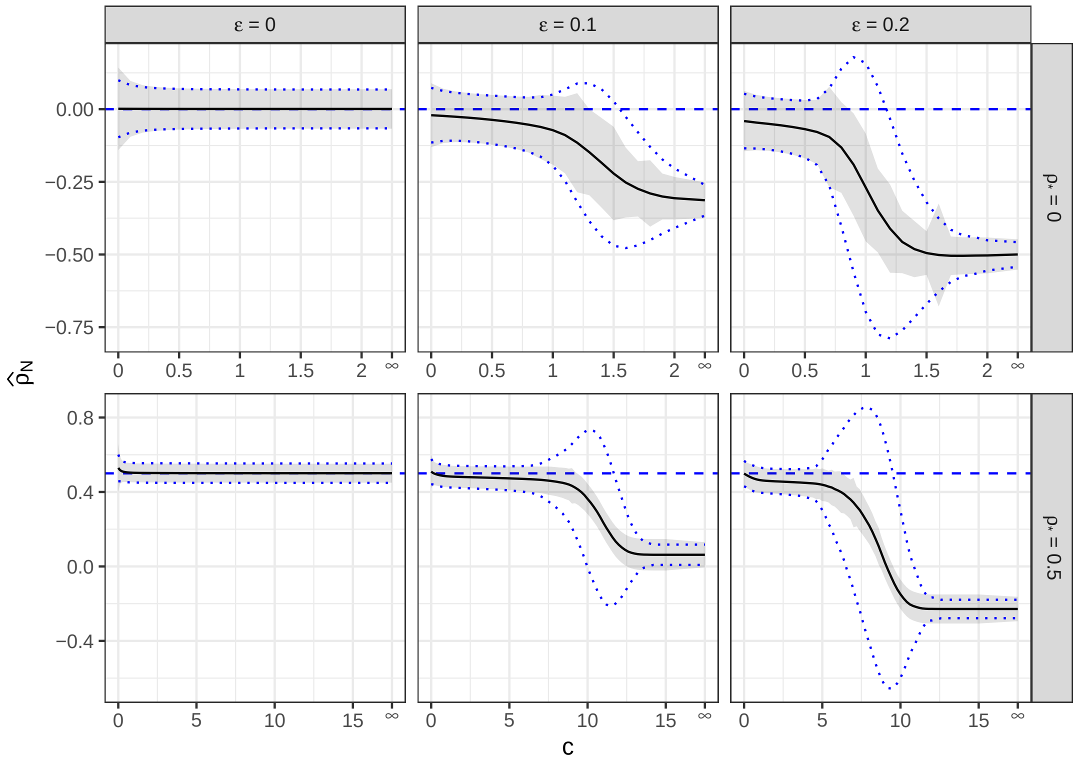

Figure C.1: Results across 5,000 simulated datasets for various choices of the tuning constant c (x-axis), contamination fractions ε (columns), and true polychoric correlation coefficients ρ_* (rows). Solid black lines indicate the average point estimate $\widehat{\rho}_N^{\text{ave}}$ and dashed blue lines the true ρ_*. Shaded gray areas visualize confidence bands using the average standard error estimate $\widehat{\text{SE}}^{\text{ave}}(\widehat{\rho}_N)$, while dotted blue lines represent confidence bands using the approximate true standard error $\text{SE}^{\text{approx}}(\widehat{\rho}_N)$.

We first focus on the top row, which corresponds to the zero-correlation setting ($\rho_* = 0$). In the absence of contamination ($\varepsilon = 0$), the point estimates are accurate for all choices of c. For strictly positive c, the standard error estimates are also accurate in the sense that they closely follow the approximate true standard errors. On the other hand, if $c = 0$, the standard error estimate deviates from the approximate true standard error. This is expected, because we estimate the standard errors based on Theorem A.1, which explicitly excludes $c = 0$ since the estimator $\widehat{\boldsymbol{\theta}}_N$ in that case is *not* asymptotically normal in the absence of contamination (see Appendix A). Introducing contamination ($\varepsilon > 0$), the point estimates stay relatively stable and accurate for c up to 1 (for $\varepsilon = 0.1$) or 0.7 (for $\varepsilon = 0.2$). For choices of c beyond these values, the estimates deteriorate fairly quickly and approach the ML estimates ($c = \infty$), which are strongly biased for either contamination fraction (absolute biases of 0.3 and 0.5, respectively). Furthermore, the standard error estimates are accurate for the same range of the tuning constant c where the point estimates are stable and close to the true value. Once c is large enough so that the point estimates abruptly move away from the true value, also the standard error estimates deteriorate and only stabilize again when the point estimates get close to the ML estimate with sufficiently large c. It follows that if the choice of c is suitable for the point estimate, it also seems to be suitable for the

standard error estimate. For such a suitable choice of c, we further observe that the standard errors of robust estimates (that is, sufficiently small values of c) tend to be larger than those of the ML estimate when contamination is present.

We now shift our attention to the bottom row in Figure C.1, corresponding to a true polychoric correlation coefficient of $\rho_* = 0.5$. For nonzero contamination ($\varepsilon = 0.1$ or $\varepsilon = 0.2$), the point estimates and standard error estimates stay fairly stable and accurate for choices of c up to about 8 and 5, respectively. For values beyond that, the point estimates again abruptly drop—and standard error estimates deteriorate—before they stabilize again around the strongly biased ML estimates. For the zero-contamination case ($\varepsilon = 0$), the standard error estimates are accurate for all choices of c, and the same applies to the point estimates except for c very close to 0. Indeed, for $c = 0$, there seems to be a small upward bias. This might be surprising because the estimator $\widehat{\boldsymbol{\theta}}_N$ is Fisher consistent for $\boldsymbol{\theta}_*$ in the zero-contamination case for all $c \geq 0$ (see Section 5.2 of the main text). It turns out that this empirical bias is a finite-sample issue that vanishes in larger sample sizes. Specifically, it is due to a finite-sample approximation error of the nonparametric estimator $\widehat{f}_N$ of the population probability mass function $f_0(x, y) = p_{xy}(\boldsymbol{\theta}_*)$, as we explain in the following.

Recall from Section 5.1 of the main text that the empirical Pearson residual at the true parameter value $\boldsymbol{\theta}_*$ of a cell $(x, y) \in \mathcal{X} \times \mathcal{Y}$ is defined as

$$\frac{\widehat{f}_N(x, y)}{p_{xy}(\boldsymbol{\theta}_*)} - 1.$$

In the absence of contamination, $\widehat{f}_N(x, y) \xrightarrow{\mathbb{P}} f_0(x, y) = p_{xy}(\boldsymbol{\theta}_*)$ as $N \to \infty$, so all Pearson residuals are asymptotically zero whenever $\varepsilon = 0$. Consequently, no cells are downweighted by the discrepancy function φ in (5.3), no matter the choice of $c \geq 0$. However, note that if the true ρ_* is moderate to large in magnitude, some of the $p_{xy}(\boldsymbol{\theta}_*)$ may be extremely small. Then, in finite sample sizes N, a nonzero $\widehat{f}_N(x, y)$ due to natural variation may be relatively large in comparison such that

$$\frac{\widehat{f}_N(x, y)}{p_{xy}(\boldsymbol{\theta}_*)} - 1 > c = 0,$$

meaning that the corresponding cell is downweighted by the discrepancy function in (5.3) despite contamination being absent. It follows from this incorrect downweighting that the ensuing estimate $\widehat{\boldsymbol{\theta}}_N$ may suffer from a finite-sample bias for the true $\boldsymbol{\theta}_*$ that is due to the finite-sample approximation error of $\widehat{f}_N$. Similar reasoning applies for positive choices of c that are close to 0. To avoid this issue, it may be preferable to choose a c that is large enough to give the Pearson residual sufficient leeway to avoid incorrect downweighting from finite-sample approximation errors when contamination is absent, while simultaneously being sufficiently small to retain robustness against contamination if present.

We stress that finite-sample approximation errors in the absence of contamination tend to be negligibly small when the true correlation is zero, i.e., $\rho_* = 0$ (see the top row of Figure C.1). Consequently, in sufficiently sized samples, we believe the risk of a false positive (significantly nonzero correlation estimate when the true correlation is zero) is small for c sufficiently away from 0.

C.5. Discussion and implications

The simulation results suggest that for a broad range of sufficiently small values of c, point estimates are relatively constant and robust to contamination, and the respective standard error estimates are accurate.

However, if the true correlation is nonzero and contamination is absent, values of c very close to 0 may result in bias due to finite-sample approximation error. Hence, in practice, one should choose c sufficiently small to retain robustness and stability, but sufficiently large to avoid finite-sample bias in certain situations. It turns out that the choice $c = 0.6$ seems to be a reasonable compromise. We therefore use this choice in all other simulations in this paper. Nevertheless, we stress that clear practical recommendations for the choice of c, preferably grounded in statistical theory, are an important avenue for future research.

D. Additional results of simulations from the main text

Sections D.1 and D.2 present additional results for the simulations from Sections 6.1 and 6.2 of the main text, respectively.

D.1. *Individual polychoric correlation coefficient*

Figure D.1 visualizes the mean squared error of the estimated parameter vector in the polychoric model. We do not include the Pearson sample correlation coefficient because it does not estimate threshold parameters. Clearly, the robust estimator remains accurate across all contamination fractions, whereas the MLE becomes increasingly biased.

In order to verify the correctness of the robust estimator's asymptotic behavior established in Theorem A.1, we further compare the correlation estimator's theoretical density with its empirical density in the simulation. Specifically, we compare these two densities for the bias with respect to the estimand, scaled by the square root of the sample size, that is, $\sqrt{N}\,(\widehat{\rho}_N - \rho_0)$. Theorem A.1 tells us that the asymptotic distribution of this term is given by $\mathrm{N}\big(0, \mathrm{SE}\,(\rho_0)^2\big)$, where $\mathrm{SE}\,(\rho_0)$ is square root of first diagonal element of the asymptotic covariance matrix $\mathbf{\Sigma}\,(\boldsymbol{\theta}_0)$, i.e., the asymptotic standard error of the correlation estimator $\widehat{\rho}_N$. The density of this distribution constitutes the theoretical density. For the empirical density, we apply a kernel density estimator to the estimates $\sqrt{N}\left(\widehat{\rho}_N^{(t)} - \rho_0\right)$, $t = 1, \ldots, T$, from the $T = 5,000$ simulation repetitions. If the asymptotic theory of Theorem A.1 is correct, the empirical and theoretical densities should be close to each other, save for some finite-sample approximation error. We repeat this procedure for the MLE. However, in the presence of contamination, the two densities are expected to be quite different from each other because the MLE's theoretical density

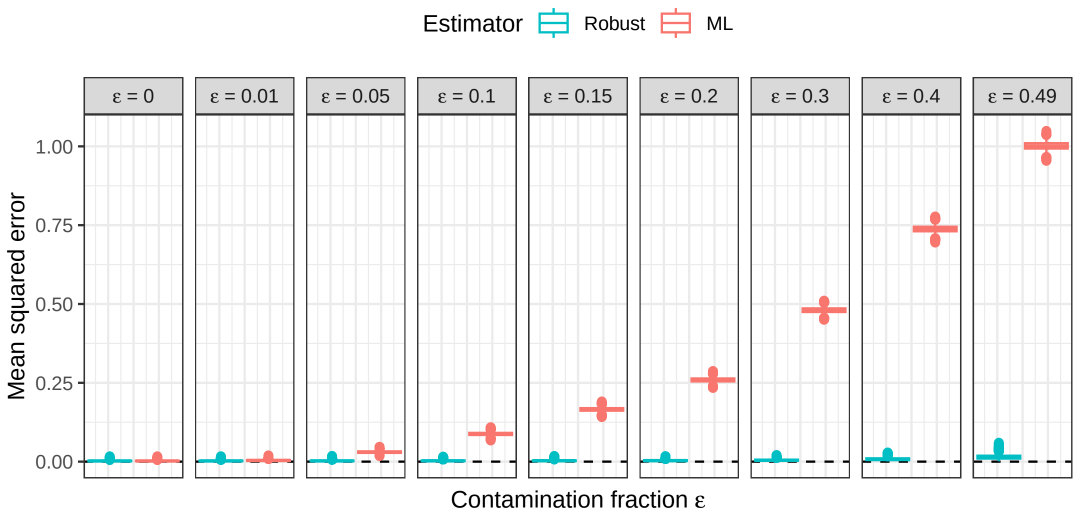

Figure D.1: Boxplot visualization of the mean squared error of the estimated parameter vector $\widehat{\boldsymbol{\theta}}_N$ with respect to the true $\boldsymbol{\theta}_*$, for various contamination fractions in the misspecified polychoric model across 5,000 repetitions. The estimators are the proposed robust estimator with $c = 0.6$ (left) and the MLE (right).

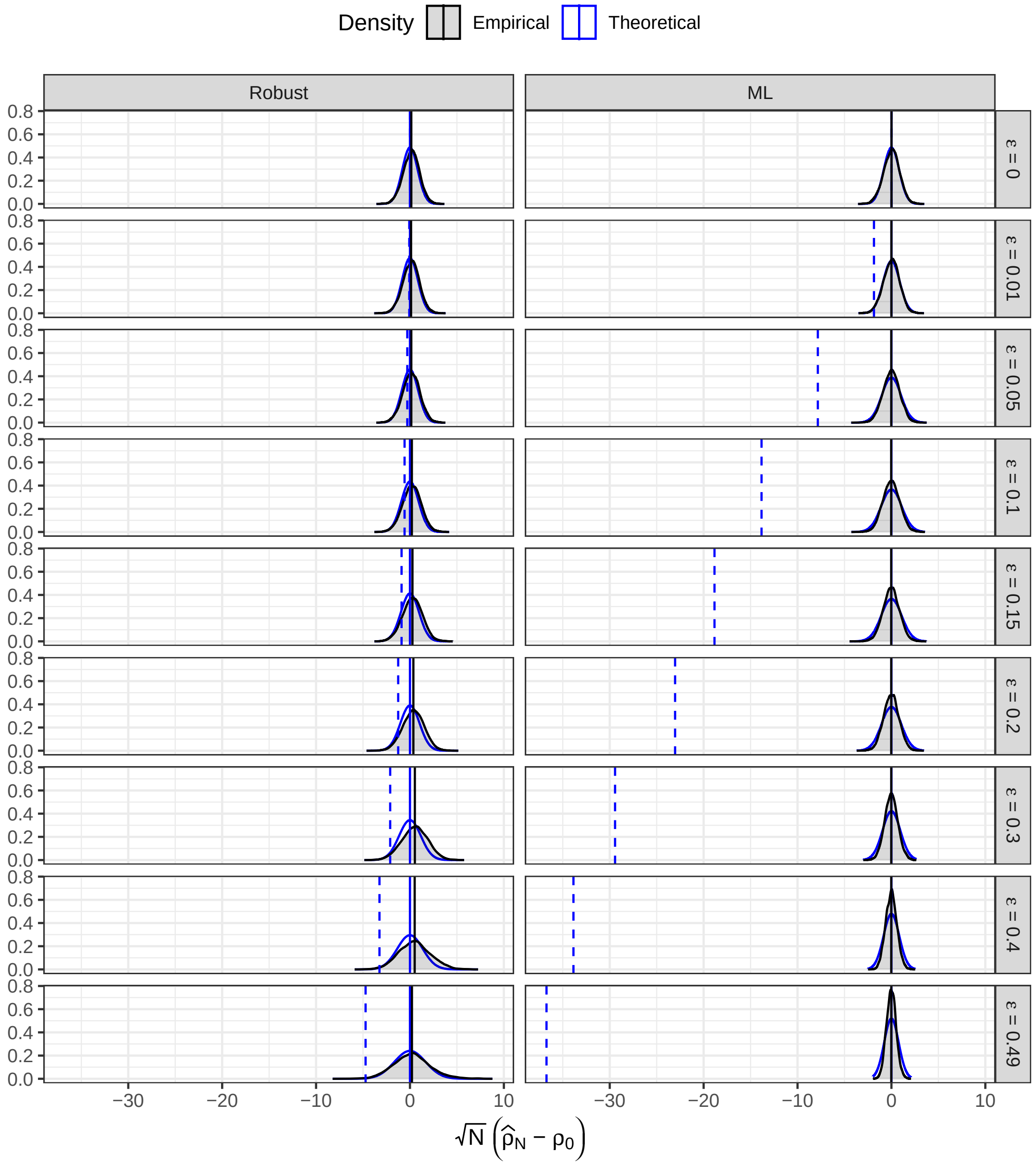

Figure D.2: Visualization of the empirical density across 5,000 simulated datasets and the theoretical density of $\sqrt{N}\,(\widehat{\rho}_N - \rho_0)$ for the robust estimator (left) and the MLE (right), for various contamination fractions (rows). The solid vertical lines correspond to the mean of a density. The blue dashed vertical lines correspond to the (scaled) population bias with respect to the true parameter, $\sqrt{N}\,(\rho_0 - \rho_*)$.

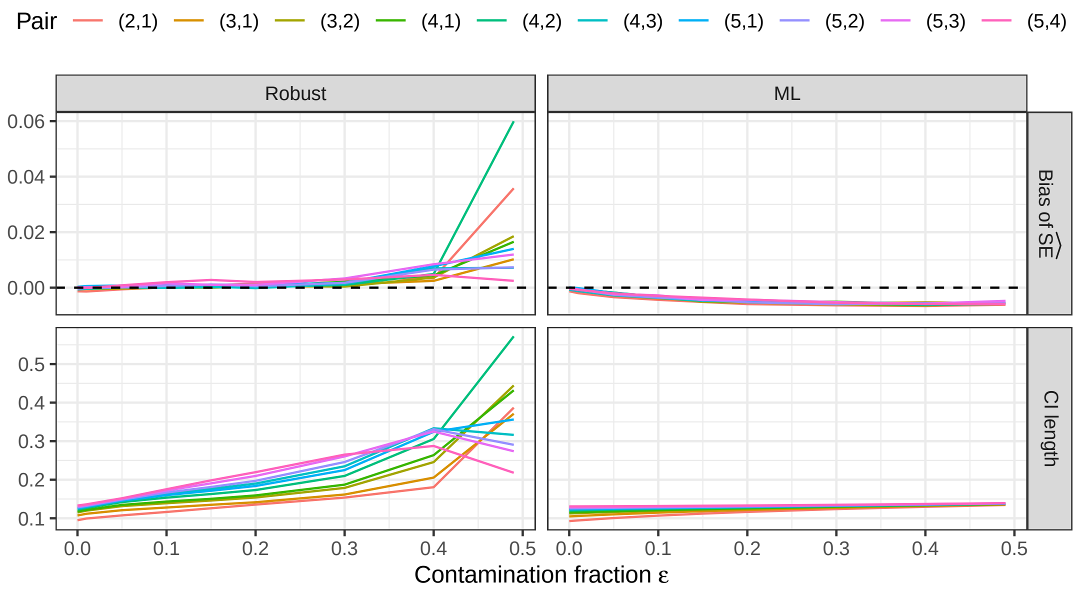

Figure D.3: Average bias of the standard error estimates (top) and average length of 95% confidence intervals (bottom) of the robust estimator with $c = 0.6$ (left) and the MLE (right) for each unique pairwise polychoric correlation coefficient (see Table 2), expressed as a function of the contamination fraction ε (x-axis). Averages are taken over 5,000 repetitions.

(based on the inverse Fisher information matrix) is derived under the then-violated assumption of latent normality.

Figure D.2 visualizes the empirical and theoretical densities of the robust estimator and the MLE (columns) for the considered contamination fractions (rows). To get a sense of bias with respect to the true correlation $\rho_* = 0.5$, the black dotted vertical line is the (appropriately scaled) difference between estimand ρ_0 and true value, that is, $\sqrt{N}(\rho_0 - \rho_*)$. The figure reveals that the for the robust estimator, the empirical and theoretical density are indeed (relatively) close throughout all contamination fractions. In contrast, the MLE's empirical density increasingly differs from the theoretical density as the contamination fraction increases, with the former having larger and larger kurtosis. In addition, the MLE's bias with respect to the true parameter is much larger than that of the robust estimator. We conclude that this exercise confirms the correctness of our asymptotic theory and further demonstrates the enhanced robustness of our proposed estimator.

D.2. Polychoric correlation matrix

Figure D.3 visualizes additional performance measures regarding inference, namely the (approximate) average bias of the standard error estimates, $\widehat{\mathrm{SE}}^{\mathrm{ave}}(\widehat{\rho}_N) - \mathrm{SE}^{\mathrm{approx}}(\widehat{\rho}_N)$, defined in Appendix C.3, and the average length of 95% confidence intervals. We observe that the standard error estimates of the robust estimator are accurate except for extremely large contamination fractions ($\epsilon \geq 0.4$), whereas those of the MLE are underestimated in the presence of contamination. Furthermore, the confidence intervals of the robust estimator tend to be wider when contamination is present compared to those of the MLE.

E. Additional simulations with distributional overlap

This appendix contains two additional simulation designs. The first generalizes the design from Section 6.1 of the main text by considering different mean shifts in the contamination distribution. The second uses contamination that manifests through correlation shifts while keeping the same mean as the model distribution. Before introducing specifics, we first discuss the role of distributional overlap, as it plays a prominent role in both designs.

E.1. The role of distributional overlap

Both simulation designs explore, among other things, the behavior of the robust estimator when the contamination distribution H substantially overlaps with the latent model distribution $\Phi_2(\cdot, \cdot; \rho_*)$ in the contaminated distribution G_ε from (4.2). In the first design, we achieve such distributional overlap by moving the mean of H towards the origin, while in the second design, overlap is induced by letting H be a bivariate standard normal as well, but with a flipped correlation coefficient compared to the latent model distribution, i.e., $H = \Phi_2(\cdot, \cdot; -\rho_*)$. It follows that contamination in the first design manifests as mean shifts in H, while in the second design it manifests as a correlation shift in H.

In general and beyond polychoric correlation, substantial overlap between the the model distribution and the contamination distribution in the contamination model of Huber (1964), a particular identification issue may arise. For ease of exposition, even though similar arguments hold for estimating polychoric correlation, we explain this identification issue within the broader context of covariance matrix estimation. In this setting, said identification issue affects any affine equivariant robust estimator of the covariance matrix. Consider first a contamination fraction $\varepsilon = 0.5$ and a contamination distribution H so that we obtain two perfectly separated point clouds of exactly the same size. An affine equivariant robust estimator would have no way of knowing whether it should return the covariance parameters of the first group or the second group (cf. Section 6.17.1 in Maronna et al., 2018), which is the reason why the contamination fraction ε is typically restricted to the interval $[0, 0.5)$ in the Huber contamination model. If the point clouds are no longer separated but overlap, fewer points from the groups are separated such that the identification issue already occurs for contamination fractions ε of less than 0.5. This is because an affine equivariant covariance matrix estimator may successfully identify some points as outliers and downweight them, but it would have no way of identifying whether observations from the overlapping part come from the model distribution of interest or the contamination distribution, causing bias in the estimates. Riani et al. (2014) have formalized and studied this issue for various robust estimators in a regression setting.

Importantly, this is a general identification issue for all robust estimators with the relevant equivariance/invariance properties. Although such properties may not be meaningful in case of ordinal data, which need not admit a numeric interpretation, we expect this issue to also arise for our robust estimator of the polychoric correlation model due to the use of a latent continuous space. This issue could only be overcome by placing additional assumptions on the contamination distribution H in (4.2). However, since such assumptions may also be violated in practice, we believe that it is undesirable to introduce new assumptions beyond those needed for ML estimation of the polychoric model, and we refrain from doing so.

E.2. Robustness against mean shifts

In the simulations from Section 6.1 of the main text, the true polychoric correlation coefficient is given by $\rho_* = 0.5$ and the contamination distribution H in the contaminated distribution G_ε from (4.2) is set to

$$H = \mathrm{N}_2\left(\boldsymbol{\mu}_H, \begin{pmatrix} 0.25 & 0 \\ 0 & 0.25 \end{pmatrix}\right)$$

with mean vector $\boldsymbol{\mu}_H = (2.5, -2.5)^\top$. Compared to the polychoric model where the latent distribution is standard bivariate normal with correlation ρ_*, this contamination distribution is mean-shifted and has a different covariance structure.

E.2.1. Simulation design

Here, we extend the simulation design from Section 6.1 of the main text with different mean shifts. That is, we set all parameters as in Section 6.1, but we consider the mean vectors

$$\boldsymbol{\mu}_H \in \left\{ \begin{pmatrix} 0 \\ 0 \end{pmatrix}, \begin{pmatrix} 0.5 \\ -0.5 \end{pmatrix}, \begin{pmatrix} 1 \\ -1 \end{pmatrix}, \begin{pmatrix} 1.5 \\ -1.5 \end{pmatrix}, \begin{pmatrix} 2 \\ -2 \end{pmatrix}, \begin{pmatrix} 2.5 \\ -2.5 \end{pmatrix} \right\}.$$

Figure E.1 visualizes an example dataset, which illustrates that the considered mean shifts result in negative leverage points with increasing leverage. For instance, the choice $\boldsymbol{\mu}_H = (0, 0)^\top$ primarily inflates the center cell $(x, y) = (3, 3)$ after discretization, $\boldsymbol{\mu}_H = (1, -1)^\top$ the cell $(x, y) = (4, 2)$, and $\boldsymbol{\mu}_H = (2.5, -2.5)^\top$ the cell $(x, y) = (5, 1)$. As such, these choices of mean vectors gradually move the contaminated data points (orange point clouds in Figure E.1 away from the origin. We expect that the stronger the leverage, the more bias the non-robust estimators will incur (cf. Welz et al., 2024). Hence, the added value of our proposed estimator should gradually increase as $\boldsymbol{\mu}_H$ moves away from the origin.

Intuitively, one may think of the considered mean-shifted contamination as careless respondents who consider only certain response patterns irrespective of the item content. Some may be straighliners after recoding of negatively keyed items: an inflated cell $(5, 1)$ corresponds to recoded responses of straightliners who exclusively chose the 1st or 5th response category, $(4, 2)$ to straightlining at the 2nd or 4th category, and $(3, 3)$ to straightlining at the central response category.

For evaluation, we compute the same performance measures as in Section 6.1 of the main text. In addition, we compute the average empirical loss function at the point estimate, $L\left(\widehat{\boldsymbol{\theta}}_N, \widehat{f}_N\right)$. With increasing contamination fraction (i.e., the polychoric model becoming more and more misspecified), we expect the empirical loss to gradually increase (indicating deteriorating fit of the polychoric model).

E.2.2. Results

Figure E.2 visualizes the simulation results with different performance measures in the rows and different contamination mean vectors $\boldsymbol{\mu}_H$ in the columns. The results for $\boldsymbol{\mu}_H = (2.5, -2, 5)^\top$ in the rightmost column have already been described in Section 6.1 and demonstrate a substantial improvement of the proposed estimator over the existing methods, even at high contamination fractions. Generally speaking, the robustness benefit of our estimator increases as the contamination mean $\boldsymbol{\mu}_H$ moves away from the origin.

For small mean shifts (up to $\boldsymbol{\mu}_H = (1, -1)^\top$), the latent data points from the polychoric model and the contamination completely or mostly overlap to form one coherent point cloud (see Figure E.1). In these settings, the three estimators perform similarly: while standard errors are estimated accurately, bias increases with higher contamination fractions and a larger mean shift (due to higher leverage). Yet,

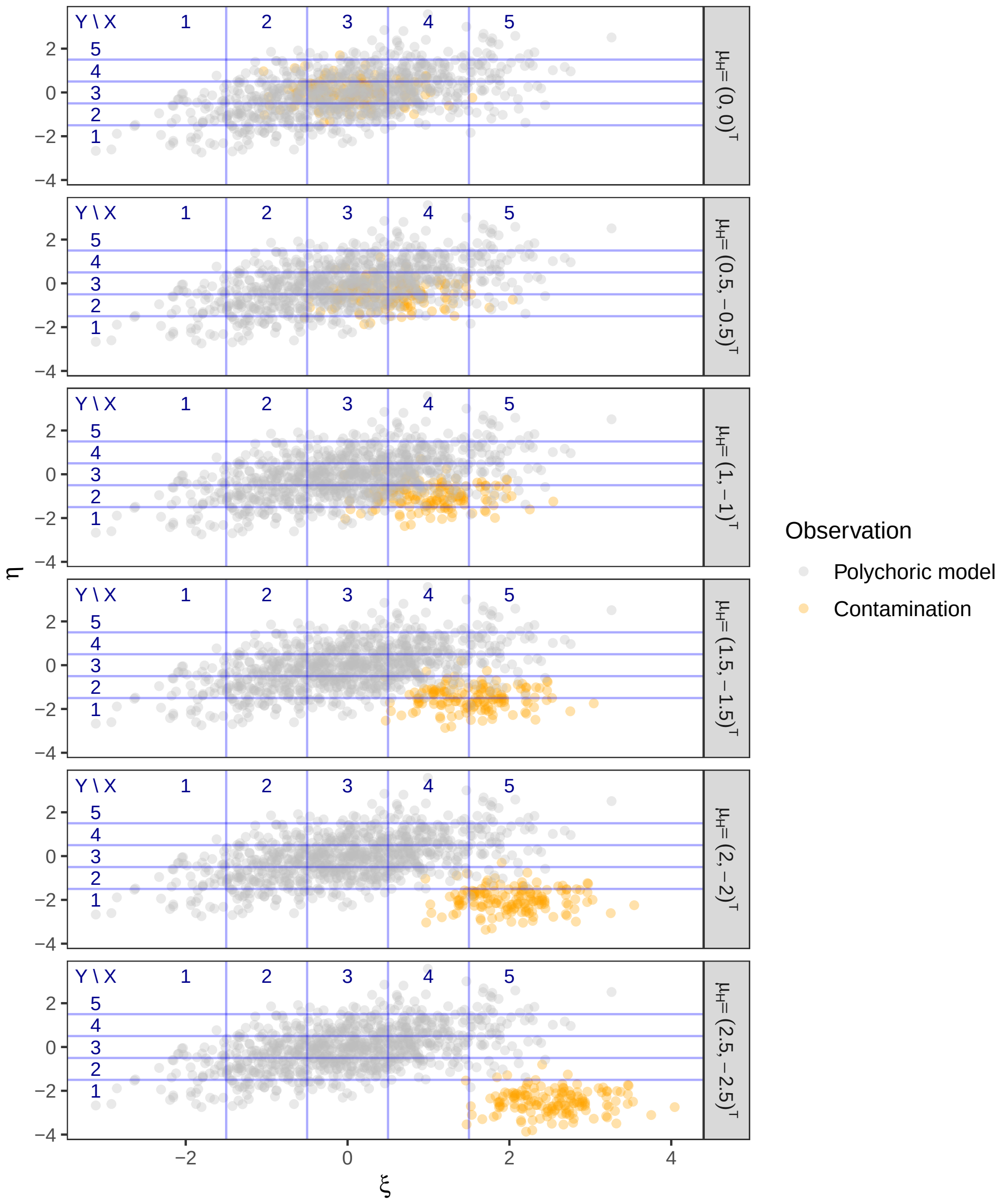

Figure E.1: Simulated example dataset with different mean vectors $\boldsymbol{\mu}_H$ of the contamination distribution (rows). The gray dots represent random draws of (ξ, η) from the polychoric model with $\rho_* = 0.5$, whereas orange dots represent draws from the contamination distribution with mean $\boldsymbol{\mu}_H$, variances $(0.25, 0.25)^\top$, and zero correlation. The contamination fraction is $\varepsilon = 0.15$ here, and we simulate $N = 1,000$ data points. The blue lines indicate the locations of the thresholds.

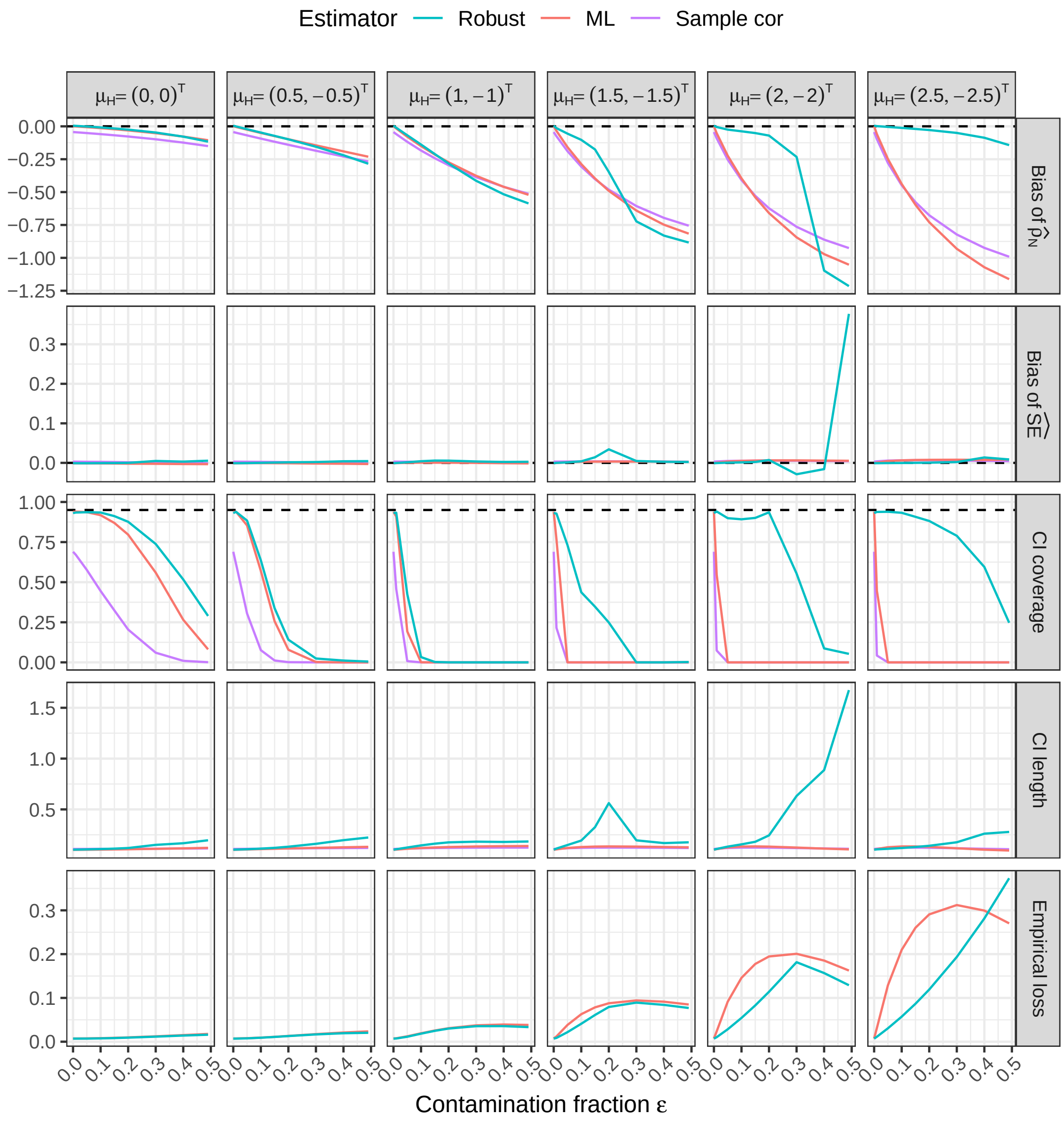

Figure E.2: Average bias of the point estimates and standard error estimates, coverage and average length of 95% confidence intervals, and average empirical loss (rows), for different mean vectors $\boldsymbol{\mu}_H$ of the contamination distribution (columns), expressed as a function of the contamination fraction ε (x-axis). Results are aggregated over 5,000 repetitions. The estimators are the robust estimator with $c = 0.6$, the MLE, and the Pearson sample correlation. Note that we include the empirical loss only for estimators of the polychoric model.

at least for $\boldsymbol{\mu}_H = (0, 0)^\top$, the robust estimator yields better confidence interval coverage close to 90% or higher for contamination fraction $\varepsilon \leq 0.2$. Looking at the empirical loss, we find that the loss of the robust estimator is nearly identical to that of the MLE for these three mean shifts, meaning that the Pearson residuals are rarely large enough to to fall within the linear part of the discrepancy function of the robust estimator. Since the latent data form one coherent point cloud, the polychoric model still fits (reasonably) well but with different parameter values.

Then at $\boldsymbol{\mu}_H = (1.5, -1.5)^\top$, the latent point clouds start to separate to some extent, and even though the robust estimator is still biased, it clearly improves upon the nonrobust estimators for contamination fractions $\varepsilon \leq 0.15$, where its standard errors are also estimated accurately. At $\boldsymbol{\mu}_H = (2, -2)^\top$, the robust estimator exhibits very little bias, accurate standard error estimates, and confidence interval coverage close to the nominal level for contamination fractions $\varepsilon \leq 0.2$. The nonrobust estimators, on the other hand, are severely influenced by the contamination even for small ε. At the high contamination fraction $\varepsilon = 0.3$, the bias of the robust estimator is quite pronounced, but much less than that of the nonrobust estimators. However, the robust estimator then sharply deteriorates such that for extreme contamination fractions $\varepsilon \geq 0.4$, its bias is even worse than that of the nonrobust estimators. To understand this phenomenon, we again look at model fit as indicated by the empirical loss. For the robust estimator, the model fit at first deteriorates as the contamination fraction increases, but it *improves* again for extreme contamination fractions. What we observe here is the identification issue discussed in Section E.1. Although the latent data points from the polychoric model and the contamination are nearly separated, there is still some overlap between the point clouds (see Figure E.1). Hence, for extreme contamination fractions, the robust estimator can achieve a better fit by modeling observations from the contamination and the overlapping part of the model distribution—while downweighting other observations that are generated by the true polychoric model. The same phenomenon, albeit less pronounced, occurs for the shifted mean $\boldsymbol{\mu}_H = (1.5, -1.5)^\top$.[1] Nevertheless, we stress that such an issue is unlikely in practice, unless there are severe problems with data quality. Contamination fractions beyond 30% are extreme, and it is questionable whether modeling such severely compromised data is meaningful to begin with.

In summary, the robust estimator requires at least partial separation between the model distribution and the contamination to offer an improvement over the nonrobust estimators. Yet, the larger the overlap between the distributions, the smaller the bias for all estimators due to the lower leverage of the contamination. Moreover, the robust estimator yields substantial gains in robustness where it matters most: in settings with high leverage, where the nonrobust estimators are severely influenced even by small amounts of contamination, whereas the robust estimator remains stable across realistic contamination fractions.

E.3. Robustness against correlation shifts

Another intuitive manifestation of contamination lies in the correlation structure of the latent variables (ξ, η). Consider, for instance, a (perhaps poorly designed) negatively-worded item. Some participants may be attentive enough to catch the general context of the item, but they may miss crucial details such as a negation. When correlating this item to another, responses of such participants would therefore invert the correlation pattern of fully attentive respondents. In general, contamination due to correlation

[1] Another consequence of this identification issue is that the robust estimator's estimated asymptotic covariance matrix $\widehat{\boldsymbol{\Sigma}}_N\left(\widehat{\boldsymbol{\theta}}_N\right)$ may not be invertible. In our simulation results with 5,000 repetitions, for mean vector $\boldsymbol{\mu}_H = (2, -2)^\top$, this occurred 9 times at $\varepsilon = 0.4$ and once at $\varepsilon = 0.49$. For $\boldsymbol{\mu}_H = (1.5, -1.5)^\top$, it occurred 13 times at $\varepsilon = 0.4$ and once at $\varepsilon = 0.49$. Since standard errors cannot be computed in such cases, they were excluded from the aggregation of the inference performance measures in Figure E.2.

shifts is difficult to detect and robustify against when the mean remains equal, as it usually induces substantial overlap with the model distribution (cf. Section E.1).

E.3.1. Simulation design

We again follow the same basic simulation design as in Section 6.1, with the model distribution of interest being the bivariate standard normal distribution $\Phi_2(\cdot, \cdot; \rho_*)$. But now the contamination distribution H is also a bivariate standard normal distribution with sign-flipped correlation, i.e., $H = \Phi_2(\cdot, \cdot; -\rho_*)$. Note that the contamination distribution H has the same zero mean as the model distribution. All other parameters are kept as in Section 6.1 of the main text, except that we extend the values of the true correlation to $\rho_* \in \{0.5, 0.7, 0.9\}$.

Figure E.3 provides an illustrative visualization of the data generating process. While we generate $N = 1,000$ observations in the simulations, the plot shows 10,000 latent data points for a clearer visualization. Clearly, the model distribution (gray points) and the contamination (orange points) largely overlap for $\rho_* = 0.5$, but they become more dissimilar as $\rho_* = 0.5$ increases except for some remaining overlap around the shared mean. We therefore expect the robust estimator to struggle to improve over the nonrobust estimators for $\rho_* = 0.5$, but it should yield robustness gains as ρ_* increases.

E.3.2. Results

Figure E.4 visualizes the performance measures and the empirical loss in the rows and the different true correlation coefficients ρ_* in the columns. At correlation $\rho_* = 0.5$, the robust estimator yields perhaps a minor improvement over the nonrobust estimators, at least in terms of confidence interval coverage, but all three estimators are very similar and become gradually more biased with increasing contamination fraction ε. Looking at the empirical loss confirms that the loss of the robust estimator is only marginally smaller than that of the MLE, indicating only a minor downweighting of Pearson residuals.

Increasing the true correlation to $\rho_* = 0.7$, the robust estimator performs notably better than the nonrobust estimators. Although it still exhibits considerable and gradually increasing bias, this bias is roughly half that of the nonrobust estimators for contamination fractions $\varepsilon \leq 0.2$, and its standard error estimates are accurate for $\varepsilon \leq 0.15$.

For true correlation $\rho_* = 0.9$, the model distribution and the contamination distribution are sufficiently different (see Figure E.3) such that our robust estimator yields substantial robustness gains. While the nonrobust estimators gradually become more biased, the robust estimator displays minimal bias and accurate standard error estimates up to high contamination fractions of $\varepsilon \leq 0.3$. Furthermore, confidence interval coverage remains near the nominal level of 95% for $\varepsilon \leq 0.15$ and still close to 85% for $\varepsilon = 0.2$.

Finally, it is no coincidence that for all three choices of the correlation coefficient ρ_*, the three estimators arrive at a bias of about $-\rho_*$ at contamination level $\varepsilon = 0.49$, which indicates that a near-zero correlation is estimated. Indeed, we have two groups of observations of about equal size that are symmetric to each other about the origin, one with correlation ρ_* and one with correlation $-\rho_*$. It is not surprising——due to the overlap between the groups even for the robust estimator—that the best fit can be attained at a correlation estimate around 0.

To summarize, we observe again that if the model distribution and the contamination distribution have large overlap, it is difficult for the robust estimator to identify the contamination (cf. the discussion in Appendix E.1) and consequently, to improve upon the nonrobust estimators. Nevertheless, we observe a clear benefit of the robust estimator as correlation increases and overlap decreases, even for (relatively) high contamination fractions.

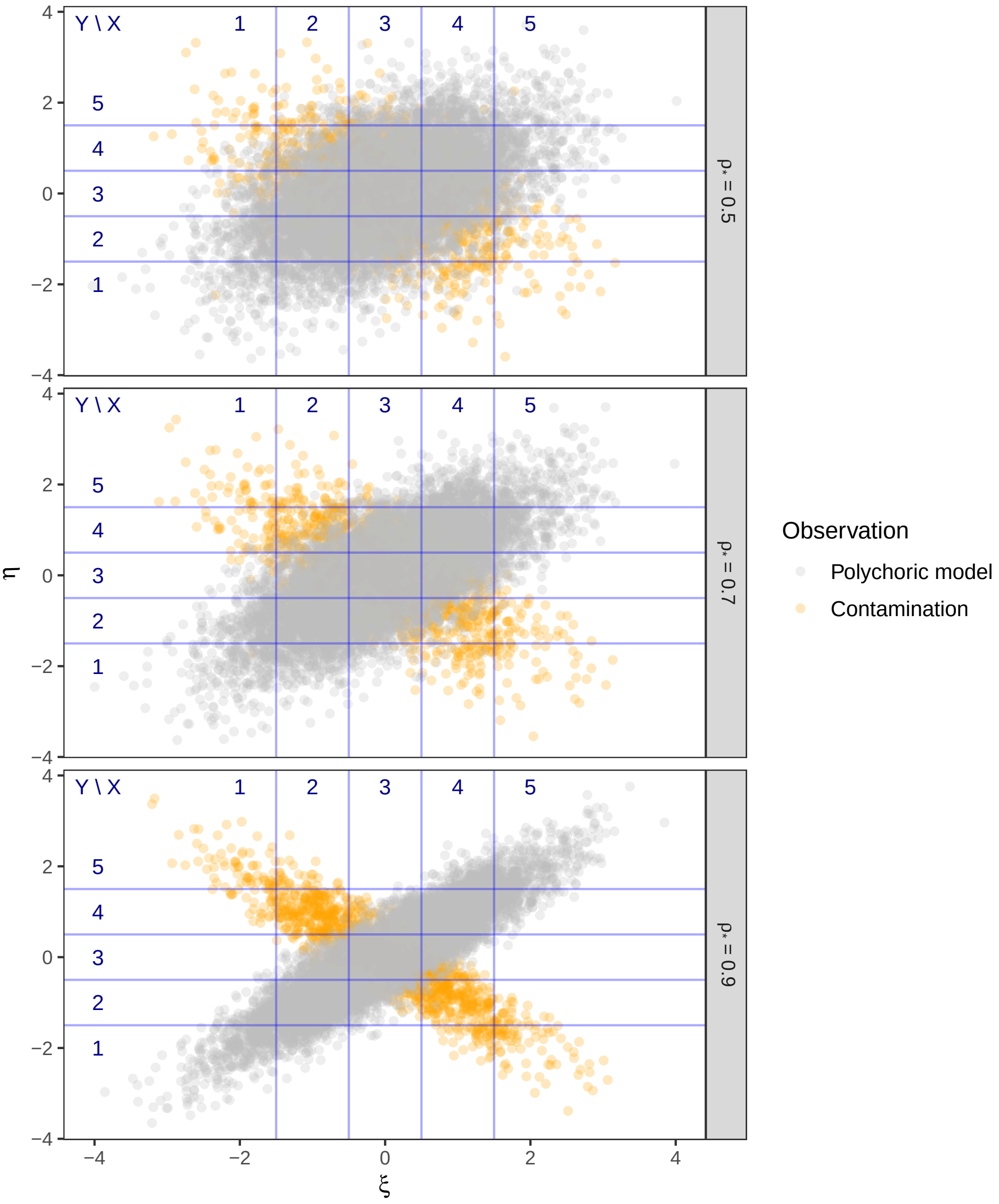

Figure E.3: Illustrative examples of the data generating process with different correlation coefficients ρ_* (rows). The gray dots represent random draws of (ξ, η) from the polychoric model with correlation ρ_*, whereas orange dots represent draws from the contamination distribution with correlation $-\rho_*$. The contamination fraction is $\varepsilon = 0.15$ here, and we generate $N = 10,000$ data points for a clearer visualization. The blue lines indicate the locations of the thresholds.

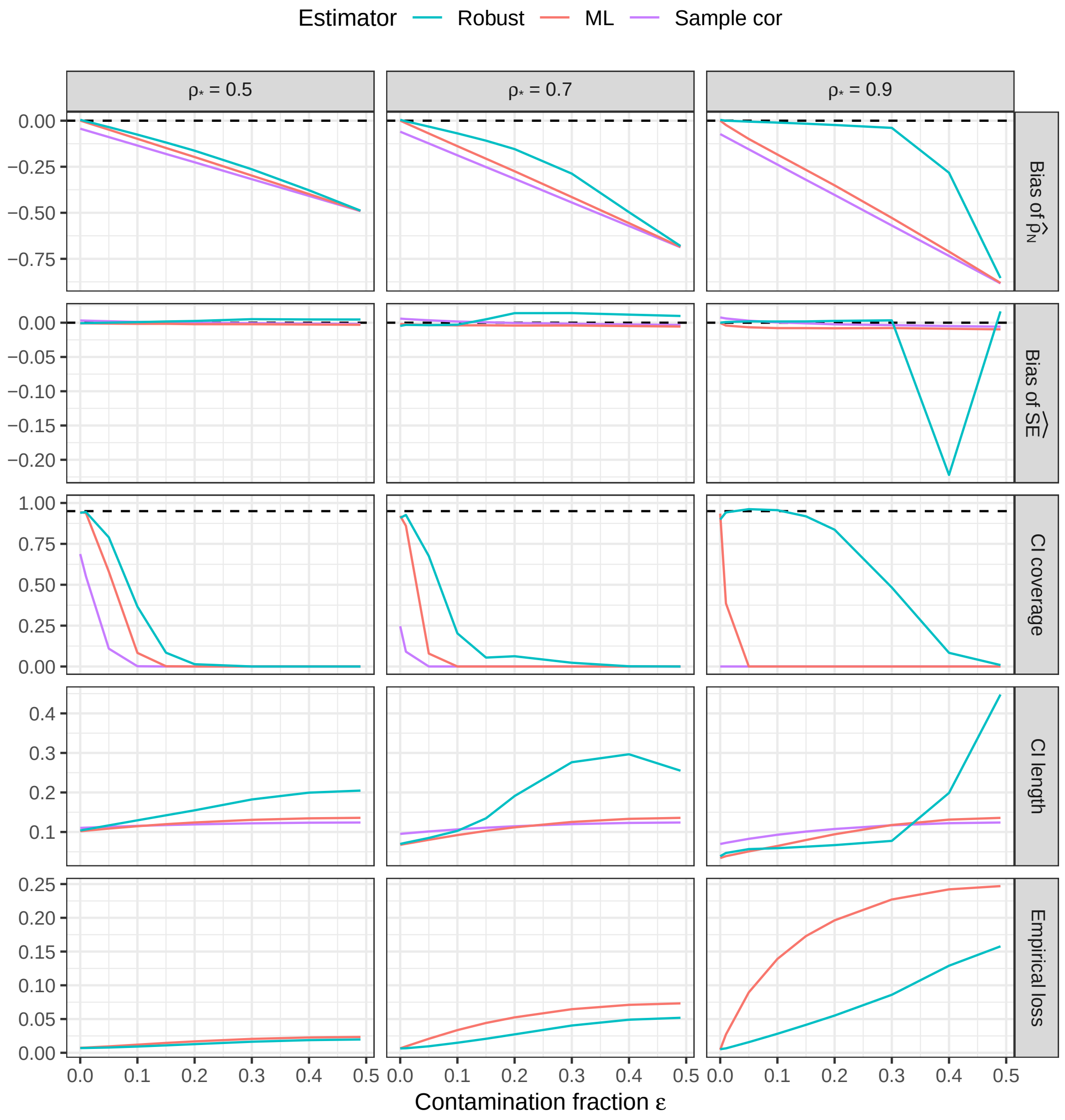

Figure E.4: Average bias of the point estimates and standard error estimates, coverage and average length of 95% confidence intervals, and average empirical loss (rows), for different correlation coefficients ρ_* (columns), expressed as a function of the contamination fraction ε (x-axis). Results are aggregated over 5,000 repetitions. The estimators are the robust estimator with $c = 0.6$, the MLE, and the Pearson sample correlation. Note that we include the empirical loss only for estimators of the polychoric model.

E.3.3. Implications for the empirical application

It turns out that our findings for $\rho_* = 0.9$ are remarkably similar to that of the empirical application from Section 7 with the item pair corresponding to the polar opposite adjectives “not envious” and “envious”. The main difference is that in the simulations, the true correlation coefficient is strongly positive (with the correlation coefficient of the contamination being sign-flipped), whereas we expect a strongly negative correlation between the aforementioned items in the empirical application.

In our simulation with true correlation $\rho_* = 0.9$ and contamination fraction $\varepsilon = 0.15$, we obtain average correlation estimates of just below 0.9 for the robust estimator, just above 0.6 for the MLE, and just below 0.6 for the sample correlation (see Figure E.4). In the empirical application, on the other hand, we obtain estimates of −0.925 with the robust estimator, −0.618 with the MLE, and −0.562 with the Pearson sample correlation (see Table 3). The robust estimator thereby assigns high Pearson residuals to the response cells $(x, y) \in \{(1, 1), (1, 2), (2, 1), (2, 2), (4, 4), (4, 5), (5, 4), (5, 5)\}$, and the empirical frequencies of these cells sum up to 11.4% (see Table 4). As discussed in Section 7.2, these cells likely correspond to careless respondents, as it is contradictory that both adjectives or neither adjective describe their personality accurately (cf. Arias et al., 2020). It seems plausible that these respondents simply overlooked the negation and responded to the item “envious” twice, in which case the correlation within this group is sign-flipped compared to the correlation within attentive respondents. Assuming that similar careless respondents also choose the middle response categories on both items, an overall prevalence of careless respondents around 15% seems plausible, too.

In short, the findings from the empirical application seem to match both the simulation design and the corresponding results. Not only does this strengthen the validity of our findings in the empirical application, it also demonstrates that our simulation design with such a strong correlation is of practical relevance.

F. Additional results for the empirical application

This section contains additional results of the empirical application from Section 7. Table F.1 lists the unipolar markers of the three Big Five scales used by Arias et al. (2020), namely *extroversion, conscientiousness,* and *neuroticism*. Tables F.2–F.4 contain the (polychoric) correlation matrices of the items in each scale, estimated by maximum likelihood and our robust estimator, while Figures F.1–F.3 visualize the absolute difference between the two estimators for each pairwise correlation. Furthermore, Table F.5 contains the cellwise Pearson residuals for the item pair "not envious" and "envious" in the *neuroticism* scale. Figure F.4 provides a corresponding visualization.

	Adjective marker pairs		
Construct	Index	Positive (P)	Negative (N)
Extroversion (E)	1	extraverted	introverted
	2	energetic	unenergetic
	3	talkative	silent
	4	bold	timid
	5	assertive	unassertive
	6	adventurous	unadventurous
Conscientiousness (C)	1	organized	disorganized
	2	responsible	irresponsible
	3	conscientious	negligent
	4	practical	impractical
	5	thorough	careless
	6	hardworking	lazy
Neuroticism (N)	1	calm	angry
	2	relaxed	tense
	3	at ease	nervous
	4	not envious	envious
	5	stable	unstable
	6	contented	discontented

Table F.1: Unipolar markers of three Big Five personality traits (Goldberg, 1992). Each trait is measured by six pairs of items, where each item is a single English adjective. Each item pair consists of a positive and negative item. We explain item identifiers by means of the following example. Item "C3_P" refers to the positive (P) item in the 3rd pair of the conscientiousness (C) scale, that is, adjective "conscientious", whereas "N1_N" would refer to "angry".

	N1_P	N1_N	N2_P	N2_N	N3_P	N3_N	N4_P	N4_N	N5_P	N5_N	N6_P	N6_N
N1_P	1.00	-0.47	0.80	-0.58	0.79	-0.56	0.30	-0.26	0.63	-0.54	0.49	-0.39
N1_N	-0.47	1.00	-0.48	0.58	-0.49	0.54	-0.26	0.45	-0.47	0.68	-0.43	0.63
N2_P	0.80	-0.48	1.00	-0.66	0.85	-0.60	0.32	-0.32	0.64	-0.50	0.60	-0.56
N2_N	-0.58	0.58	-0.66	1.00	-0.70	0.76	-0.37	0.49	-0.48	0.60	-0.35	0.55
N3_P	0.79	-0.49	0.85	-0.70	1.00	-0.62	0.35	-0.39	0.66	-0.52	0.59	-0.57
N3_N	-0.56	0.54	-0.60	0.76	-0.62	1.00	-0.42	0.49	-0.52	0.58	-0.37	0.53
N4_P	0.30	-0.26	0.32	-0.37	0.35	-0.42	1.00	-0.92	0.35	-0.30	0.30	-0.33
N4_N	-0.26	0.45	-0.32	0.49	-0.39	0.49	-0.92	1.00	-0.39	0.50	-0.33	0.53
N5_P	0.63	-0.47	0.64	-0.48	0.66	-0.52	0.35	-0.39	1.00	-0.82	0.59	-0.55
N5_N	-0.54	0.68	-0.50	0.60	-0.52	0.58	-0.30	0.50	-0.82	1.00	-0.44	0.61
N6_P	0.49	-0.43	0.60	-0.35	0.59	-0.37	0.30	-0.33	0.59	-0.44	1.00	-0.75
N6_N	-0.39	0.63	-0.56	0.55	-0.57	0.53	-0.33	0.53	-0.55	0.61	-0.75	1.00

(a) Robust estimates

	N1_P	N1_N	N2_P	N2_N	N3_P	N3_N	N4_P	N4_N	N5_P	N5_N	N6_P	N6_N
N1_P	1.00	-0.37	0.71	-0.50	0.69	-0.49	0.27	-0.24	0.58	-0.47	0.42	-0.32
N1_N	-0.37	1.00	-0.40	0.55	-0.39	0.48	-0.19	0.40	-0.39	0.60	-0.32	0.57
N2_P	0.71	-0.40	1.00	-0.55	0.75	-0.54	0.26	-0.26	0.55	-0.41	0.53	-0.47
N2_N	-0.50	0.55	-0.55	1.00	-0.54	0.65	-0.24	0.42	-0.41	0.57	-0.31	0.52
N3_P	0.69	-0.39	0.75	-0.54	1.00	-0.53	0.29	-0.28	0.63	-0.44	0.52	-0.48
N3_N	-0.49	0.48	-0.54	0.65	-0.53	1.00	-0.29	0.43	-0.45	0.58	-0.29	0.47
N4_P	0.27	-0.19	0.26	-0.24	0.29	-0.29	1.00	-0.62	0.26	-0.20	0.18	-0.20
N4_N	-0.24	0.40	-0.26	0.42	-0.28	0.43	-0.62	1.00	-0.33	0.46	-0.22	0.44
N5_P	0.58	-0.39	0.55	-0.41	0.63	-0.45	0.26	-0.33	1.00	-0.70	0.53	-0.46
N5_N	-0.47	0.60	-0.41	0.57	-0.44	0.58	-0.20	0.46	-0.70	1.00	-0.35	0.57
N6_P	0.42	-0.32	0.53	-0.31	0.52	-0.29	0.18	-0.22	0.53	-0.35	1.00	-0.58
N6_N	-0.32	0.57	-0.47	0.52	-0.48	0.47	-0.20	0.44	-0.46	0.57	-0.58	1.00

(b) Maximum likelihood estimates

Table F.2: Estimated correlation matrices of the items in the *neuroticism* scale from the data in Arias et al. (2020, Sample 1, $N = 725$) using the robust estimator with $c = 0.6$ (top) and the MLE (bottom). The items are "calm" (N1_P), "angry" (N1_N), "relaxed" (N2_P), "tense" (N2_N), "at ease" (N3_P), "nervous" (N3_N), "not envious" (N4_P), "envious" (N4_N), "stable" (N5_P), "unstable" (N5_N), "contented" (N6_P), and "discontented" (N6_N). For the item naming given in parentheses, items with identical identifier (the integer after the first "N") are polar opposites, where a last character "P" refers to the positive opposite and "N" to the negative opposite.

	E1_P	E1_N	E2_P	E2_N	E3_P	E3_N	E4_P	E4_N	E5_P	E5_N	E6_P	E6_N
E1_P	1.00	-0.87	0.55	-0.34	0.75	-0.62	0.58	-0.58	0.54	-0.45	0.55	-0.39
E1_N	-0.87	1.00	-0.40	0.36	-0.67	0.63	-0.52	0.62	-0.52	0.51	-0.36	0.37
E2_P	0.55	-0.40	1.00	-0.84	0.50	-0.32	0.56	-0.38	0.55	-0.43	0.57	-0.44
E2_N	-0.34	0.36	-0.84	1.00	-0.30	0.35	-0.40	0.43	-0.41	0.54	-0.45	0.53
E3_P	0.75	-0.67	0.50	-0.30	1.00	-0.71	0.50	-0.51	0.52	-0.50	0.42	-0.28
E3_N	-0.62	0.63	-0.32	0.35	-0.71	1.00	-0.38	0.62	-0.47	0.47	-0.30	0.37
E4_P	0.58	-0.52	0.56	-0.40	0.50	-0.38	1.00	-0.55	0.72	-0.64	0.61	-0.48
E4_N	-0.58	0.62	-0.38	0.43	-0.51	0.62	-0.55	1.00	-0.61	0.66	-0.33	0.44
E5_P	0.54	-0.52	0.55	-0.41	0.52	-0.47	0.72	-0.61	1.00	-0.85	0.44	-0.29
E5_N	-0.45	0.51	-0.43	0.54	-0.50	0.47	-0.64	0.66	-0.85	1.00	-0.41	0.47
E6_P	0.55	-0.36	0.57	-0.45	0.42	-0.30	0.61	-0.33	0.44	-0.41	1.00	-0.83
E6_N	-0.39	0.37	-0.44	0.53	-0.28	0.37	-0.48	0.44	-0.29	0.47	-0.83	1.00

(a) Robust estimates

	E1_P	E1_N	E2_P	E2_N	E3_P	E3_N	E4_P	E4_N	E5_P	E5_N	E6_P	E6_N
E1_P	1.00	-0.78	0.50	-0.26	0.70	-0.50	0.56	-0.42	0.51	-0.40	0.52	-0.32
E1_N	-0.78	1.00	-0.38	0.34	-0.59	0.61	-0.45	0.54	-0.47	0.50	-0.35	0.37
E2_P	0.50	-0.38	1.00	-0.65	0.43	-0.27	0.49	-0.28	0.47	-0.38	0.55	-0.39
E2_N	-0.26	0.34	-0.65	1.00	-0.24	0.34	-0.30	0.40	-0.32	0.48	-0.38	0.50
E3_P	0.70	-0.59	0.43	-0.24	1.00	-0.59	0.44	-0.36	0.46	-0.40	0.41	-0.25
E3_N	-0.50	0.61	-0.27	0.34	-0.59	1.00	-0.27	0.56	-0.35	0.45	-0.24	0.37
E4_P	0.56	-0.45	0.49	-0.30	0.44	-0.27	1.00	-0.41	0.64	-0.49	0.54	-0.34
E4_N	-0.42	0.54	-0.28	0.40	-0.36	0.56	-0.41	1.00	-0.49	0.60	-0.27	0.40
E5_P	0.51	-0.47	0.47	-0.32	0.46	-0.35	0.64	-0.49	1.00	-0.71	0.39	-0.23
E5_N	-0.40	0.50	-0.38	0.48	-0.40	0.45	-0.49	0.60	-0.71	1.00	-0.34	0.45
E6_P	0.52	-0.35	0.55	-0.38	0.41	-0.24	0.54	-0.27	0.39	-0.34	1.00	-0.68
E6_N	-0.32	0.37	-0.39	0.50	-0.25	0.37	-0.34	0.40	-0.23	0.45	-0.68	1.00

(b) Maximum likelihood estimates

Table F.3: Estimated correlation matrices of the items in the *extroversion* scale from the data in Arias et al. (2020, Sample 1, $N = 725$) using the robust estimator with $c = 0.6$ (top) and the MLE (bottom). The items are "extraverted" (E1_P), "introverted" (E1_N), "energetic" (E2_P), "unenergetic" (E2_N), "talkative" (E3_P), "silent" (E3_N), "bold" (E4_P), "timid" (E4_N), "assertive" (E5_P), "unassertive" (E5_N), "adventurous" (E6_P), and "unadventurous" (E6_N). For the item naming given in parentheses, items with identical identifier (the integer after the first "N") are polar opposites, where a last character "P" refers to the positive opposite and "N" to the negative opposite.

	C1_P	C1_N	C2_P	C2_N	C3_P	C3_N	C4_P	C4_N	C5_P	C5_N	C6_P	C6_N
C1_P	1.00	-0.89	0.57	-0.56	0.36	-0.46	0.43	-0.35	0.54	-0.56	0.49	-0.52
C1_N	-0.89	1.00	-0.58	0.64	-0.31	0.60	-0.38	0.47	-0.48	0.69	-0.52	0.61
C2_P	0.57	-0.58	1.00	-0.87	0.45	-0.68	0.62	-0.54	0.55	-0.65	0.69	-0.64
C2_N	-0.56	0.64	-0.87	1.00	-0.44	0.80	-0.57	0.74	-0.50	0.76	-0.61	0.66
C3_P	0.36	-0.31	0.45	-0.44	1.00	-0.43	0.42	-0.46	0.17	-0.41	0.40	-0.26
C3_N	-0.46	0.60	-0.68	0.80	-0.43	1.00	-0.48	0.70	-0.52	0.78	-0.55	0.59
C4_P	0.43	-0.38	0.62	-0.57	0.42	-0.48	1.00	-0.68	0.39	-0.47	0.44	-0.33
C4_N	-0.35	0.47	-0.54	0.74	-0.46	0.70	-0.68	1.00	-0.47	0.66	-0.42	0.45
C5_P	0.54	-0.48	0.55	-0.50	0.17	-0.52	0.39	-0.47	1.00	-0.54	0.60	-0.45
C5_N	-0.56	0.69	-0.65	0.76	-0.41	0.78	-0.47	0.66	-0.54	1.00	-0.59	0.61
C6_P	0.49	-0.52	0.69	-0.61	0.40	-0.55	0.44	-0.42	0.60	-0.59	1.00	-0.69
C6_N	-0.52	0.61	-0.64	0.66	-0.26	0.59	-0.33	0.45	-0.45	0.61	-0.69	1.00

(a) Robust estimates

	C1_P	C1_N	C2_P	C2_N	C3_P	C3_N	C4_P	C4_N	C5_P	C5_N	C6_P	C6_N
C1_P	1.00	-0.77	0.56	-0.43	0.34	-0.35	0.38	-0.26	0.51	-0.41	0.43	-0.43
C1_N	-0.77	1.00	-0.51	0.59	-0.24	0.55	-0.32	0.44	-0.43	0.61	-0.44	0.55
C2_P	0.56	-0.51	1.00	-0.70	0.42	-0.56	0.57	-0.43	0.51	-0.54	0.65	-0.55
C2_N	-0.43	0.59	-0.70	1.00	-0.40	0.75	-0.48	0.68	-0.43	0.71	-0.53	0.63
C3_P	0.34	-0.24	0.42	-0.40	1.00	-0.32	0.39	-0.34	0.44	-0.34	0.38	-0.25
C3_N	-0.35	0.55	-0.56	0.75	-0.32	1.00	-0.37	0.60	-0.38	0.72	-0.45	0.54
C4_P	0.38	-0.32	0.57	-0.48	0.39	-0.37	1.00	-0.52	0.36	-0.39	0.39	-0.31
C4_N	-0.26	0.44	-0.43	0.68	-0.34	0.60	-0.52	1.00	-0.38	0.59	-0.31	0.43
C5_P	0.51	-0.43	0.51	-0.43	0.44	-0.38	0.36	-0.38	1.00	-0.43	0.54	-0.39
C5_N	-0.41	0.61	-0.54	0.71	-0.34	0.72	-0.39	0.59	-0.43	1.00	-0.43	0.53
C6_P	0.43	-0.44	0.65	-0.53	0.38	-0.45	0.39	-0.31	0.54	-0.43	1.00	-0.61
C6_N	-0.43	0.55	-0.55	0.63	-0.25	0.54	-0.31	0.43	-0.39	0.53	-0.61	1.00

(b) Maximum likelihood estimates

Table F.4: Estimated correlation matrices of the items in the *conscientiousness* scale from the data in Arias et al. (2020, Sample 1, $N = 725$) using the robust estimator with $c = 0.6$ (top) and the MLE (bottom). The items are “calm” (C1_P), “angry” (C1_N), “relaxed” (C2_P), “tense” (C2_N), “at ease” (C3_P), “nervous” (C3_N), “not envious” (C4_P), “envious” (C4_N), “stable” (C5_P), “unstable” (C5_N), “contented” (C6_P), and “discontented” (C6_N). For the item naming given in parentheses, items with identical identifier (the integer after the first “N”) are polar opposites, where a last character “P” refers to the positive opposite and “N” to the negative opposite.

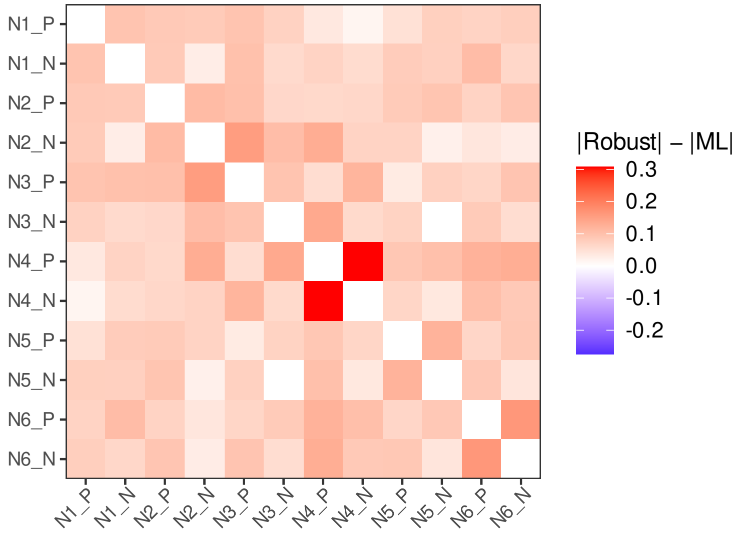

Figure F.1: Difference between absolute estimates for the polychoric correlation coefficient of the robust estimator with $c = 0.6$ and the MLE for each item pair in the *neuroticism* scale, using the data of Arias et al. (2020). The items are “calm” (N1_P), “angry” (N1_N), “relaxed” (N2_P), “tense” (N2_N), “at ease” (N3_P), “nervous” (N3_N), “not envious” (N4_P), “envious” (N4_N), “stable” (N5_P), “unstable” (N5_N), “contented” (N6_P), and “discontented” (N6_N). For the item naming given in parentheses, items with identical identifier (the integer after the first “N”) are polar opposites, where a last character “P” refers to the positive opposite and “N” to the negative opposite.

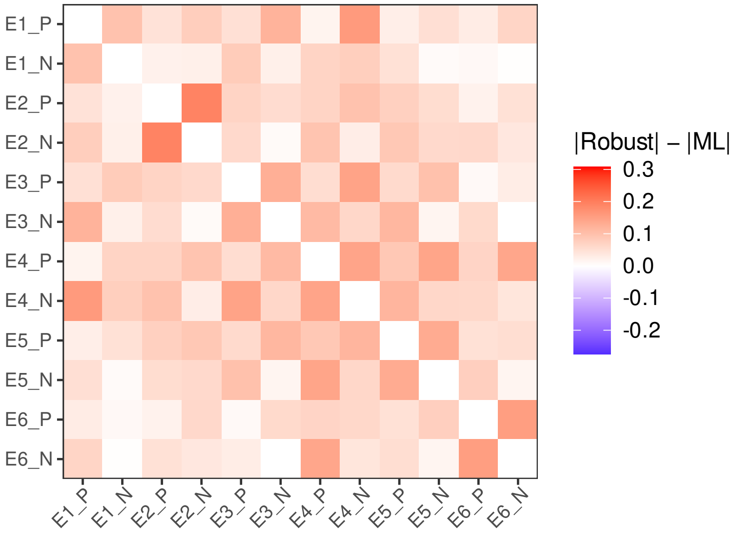

Figure F.2: Difference between absolute estimates for the polychoric correlation coefficient of the robust estimator with $c = 0.6$ and the MLE for each item pair in the *extroversion* scale, using the data of Arias et al. (2020). The items are “extraverted” (E1_P), “introverted” (E1_N), “energetic” (E2_P), “unenergetic” (E2_N), “talkative” (E3_P), “silent” (E3_N), “bold” (E4_P), “timid” (E4_N), “assertive” (E5_P), “unassertive” (E5_N), “adventurous” (E6_P), and “unadventurous” (E6_N). For the item naming given in parentheses, items with identical identifier (the integer after the first “N”) are polar opposites, where a last character “P” refers to the positive opposite and “N” to the negative opposite.

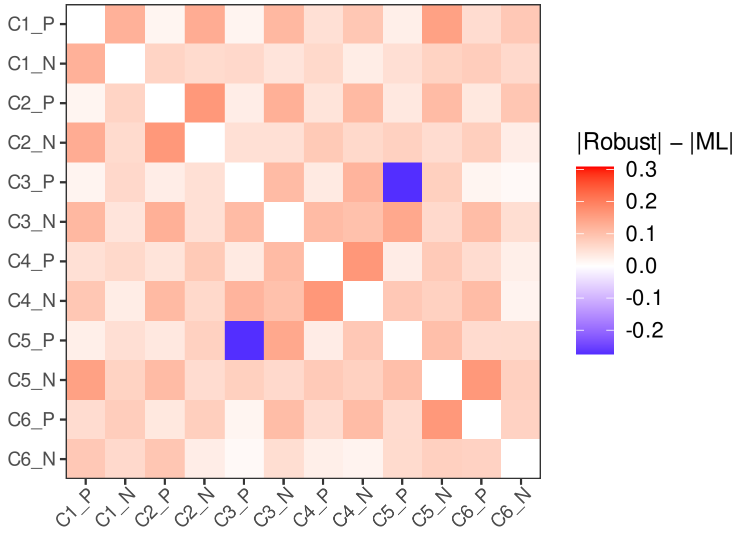

Figure F.3: Difference between absolute estimates for the polychoric correlation coefficient of the robust estimator with $c = 0.6$ and the MLE for each item pair in the *conscientiousness* scale, using the data of Arias et al. (2020). The items are “calm” (C1_P), “angry” (C1_N), “relaxed” (C2_P), “tense” (C2_N), “at ease” (C3_P), “nervous” (C3_N), “not envious” (C4_P), “envious” (C4_N), “stable” (C5_P), “unstable” (C5_N), “contented” (C6_P), and “discontented” (C6_N). For the item naming given in parentheses, items with identical identifier (the integer after the first “N”) are polar opposites, where a last character “P” refers to the positive opposite and “N” to the negative opposite.

$X\backslash Y$	1	2	3	4	5
1	9,780,473,685.31	15,982.37	10.81	0.14	−0.35
2	2,419.15	9.06	−0.20	−0.10	0.42
3	4.48	−0.35	−0.01	−0.20	76.11
4	−0.12	−0.08	−0.39	11.66	222,240.11
5	−0.11	−0.12	34.98	55,329.21	991,790,294,422.36

Table F.5: Pearson residual, $\widehat{f}_N(x, y)/p_{xy}(\widehat{\boldsymbol{\theta}}_N) - 1$, of each response (x, y) for the item pair "not envious" (X) and "envious" (Y) in the measurements of Arias et al. (2020) of the *neuroticism* scale. Estimate $\widehat{\boldsymbol{\theta}}_N$ was computed via the robust estimator with tuning constant $c = 0.6$.

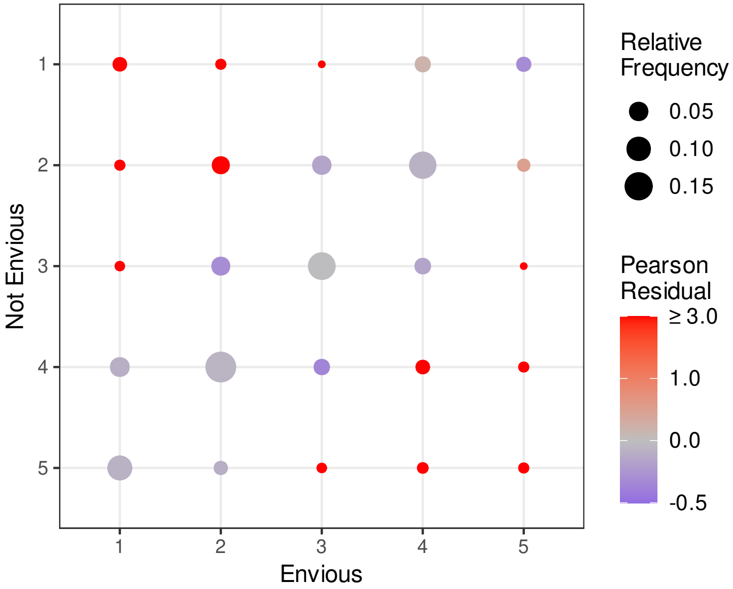

Figure F.4: Dot plot of cells for the *neuroticism* item adjective pair "not envious" and "envious" in the data of Arias et al. (2020), where each item has five Likert-type response options, anchored by "very inaccurate" (= 1) and "very accurate" (= 5). Each dot's size is proportional to the relative empirical frequency of its associated contingency table cell, $\widehat{f}_N(x, y)$, whereas its color varies by the value of the cell's PR, $\widehat{f}_N(x, y)/p_{xy}(\widehat{\boldsymbol{\theta}}_N) - 1$, at robust parameter estimate with tuning constant $c = 0.6$. Note that some cells have an extremely large PR (substantially larger than the ideal value 0; see also Table F.5), indicating a poor model fit for those cells. We therefore censored the color scale so that a fully saturated red is assigned to all cells with PR ≥ 3, i.e., the empirical frequency $\widehat{f}_N(x, y)$ being at least four times as large as the model frequency $p_{xy}(\widehat{\boldsymbol{\theta}}_N)$.

References

Arias, V. B., Garrido, L., Jenaro, C., Martinez-Molina, A., & Arias, B. (2020). A little garbage in, lots of garbage out: Assessing the impact of careless responding in personality survey data. *Behavior Research Methods*, *52*(6), 2489–2505. https://doi.org/https://doi.org/10.3758/s13428-020-01401-8

Drezner, Z., & Wesolowsky, G. O. (1990). On the computation of the bivariate normal integral. *Journal of Statistical Computation and Simulation*, *35*(1-2), 101–107. https://doi.org/10.1080/00949659008811236

Goldberg, L. R. (1992). The development of markers for the Big-Five factor structure. *Psychological Assessment*, *4*(1), 26–42. https://doi.org/https://doi.org/10.1037/1040-3590.4.1.26

Huber, P. J. (1964). Robust estimation of a location parameter. *Annals of Mathematical Statistics*, *35*(1), 73–101. https://doi.org/10.1214/aoms/1177703732

Huber, P. J. (1967). The behavior of maximum likelihood estimates under nonstandard conditions. In L. M. Le Cam & J. Neyman (Eds.), *Proceedings of the Fifth Berkeley Symposium on Mathematical Statistics and Probability* (pp. 221–234, Vol. 5.1). University of California Press.

Maronna, R. A., Martin, R. D., Yohai, V. J., & Salibián-Barrera, M. (2018). *Robust statistics: Theory and methods* (2nd). Wiley.

Olsson, U. (1979). Maximum likelihood estimation of the polychoric correlation coefficient. *Psychometrika*, *44*(4), 443–460. https://doi.org/10.1007/BF02296207

Riani, M., Atkinson, A. C., & Perrotta, D. (2014). A parametric framework for the comparison of methods of very robust regression. *Statistical Science*, *29*(1), 128–143. https://doi.org/10.1214/13-STS437

Tallis, G. M. (1962). The maximum likelihood estimation of correlation from contingency tables. *Biometrics*, *18*(3), 342–353. https://doi.org/10.2307/2527476

Welz, M. (2024). Robust estimation and inference for categorical data [arXiv:2403.11954]. https://doi.org/10.48550/arXiv.2403.11954

Welz, M., Archimbaud, A., & Alfons, A. (2024). How much carelessness is too much? quantifying the impact of careless responding [PsyArXiv:8fj6p]. https://doi.org/10.31234/osf.io/8fj6p

Welz, M., Mair, P., & Alfons, A. (2025). Robust estimation of polychoric correlation [arXiv:2407.18835]. https://doi.org/10.48550/arXiv.2407.18835

White, H. (1982). Maximum likelihood estimation of misspecified models. *Econometrica*, *50*(1), 1–26. https://doi.org/10.2307/1912526
